# A Build, Couple, Pair (B/C/P) Strategy in the Synthesis of Diindole Fused Diazamacrocycles: An Attempt to a Green Synthetic Approach

**DOI:** 10.1021/acsomega.5c08238

**Published:** 2025-09-23

**Authors:** Rofin Mangali

**Affiliations:** † School of Chemistry, 30011 Bharathidasan University, Tiruchirappalli 620 024, India; ‡ St. Francis de Sales College (Autonomous), Bengaluru 560 100, India

## Abstract

An efficient build/couple/pair (B/C/P) strategy toward the synthesis of diindole-fused diazamacrocycles was demonstrated. The water-mediated reactions of aryl aldehydes and 2-sulfonamidoindoles in the presence of a catalytic amount of tetrabutylammonium iodide (TBAI) afforded an excellent yield of *bis*(aminoindolyl)­methanes with a broad substrate scope and a good functional group tolerance. Furthermore, the bisconjugation of the free N–H groups of *bis*(aminoindolyl)­methane scaffolds with the dibromo spacers of varied chain lengths via the universal dialkylation protocol furnished diindole-fused diazamacrocycles. The obtained products were characterized by NMR, IR, and mass spectral analysis and were confirmed by single-crystal X-ray diffraction analysis. The corresponding macrocycles obtained through this protocol are new and could be potent scaffolds, and the methodology adopted in this work will be of great interest to researchers.

## Graphical abstract

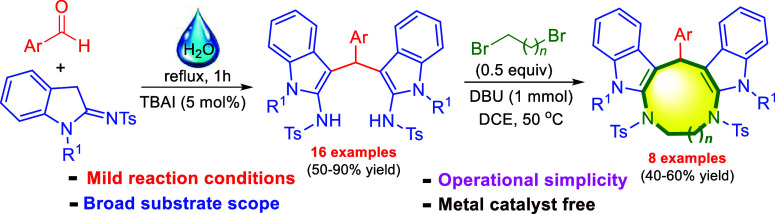

## 1 Introduction

Macrocycles^1^ are a class of large cyclic chemical entities that have gained considerable attention from synthetic chemists due to their wide-ranging impact on drug design and chemical biology, especially, medicinal, pharmaceutical, polymer, supramolecular chemistry, drug design and development, pharmaceutics, and medicinal chemistry. Macrocyclic scaffolds possess unique structural features such as conformational flexibility, good metabolic stability, good solubility, and better cell membrane permeability, which make them highly effective in pharmaceuticals.^2^ More specifically, many naturally existing *bis*-indolyl alkaloids, macrocycles,^3^ polycycles, and their synthetic analogues interconnected directly or linked with each other via one or more atoms have been reported to display in vitro cytotoxicity against several human cancer cell lines. They also determine a wide range of biological properties^4^ like antimicrobial, antituberculosis, antileishmanial, antimalarial, antioxidant, anti-Alzheimer, anti-inflammatory, antidiabetic, carbonic anhydrase II inhibition, etc. For instance, several *bis*-indole alkaloids, such as turbomycin-B^5^ **A** and caulerpin^6^ **B**, are found to exhibit antimicrobial properties. (−) Trigonoliimine^7^ **C** is known to have anticancer properties. Macrocyclic *bis*-indolylmaleimides^8^ **D** obtained through a synthetic route show good selectivity for the inhibition of protein kinase C beta (PKCβ) and anticancer properties (Figure 1). Most of the natural products bearing indole scaffolds are complex and exist in macrocyclic or heterocyclic structures.^9^

**Figure 1:**
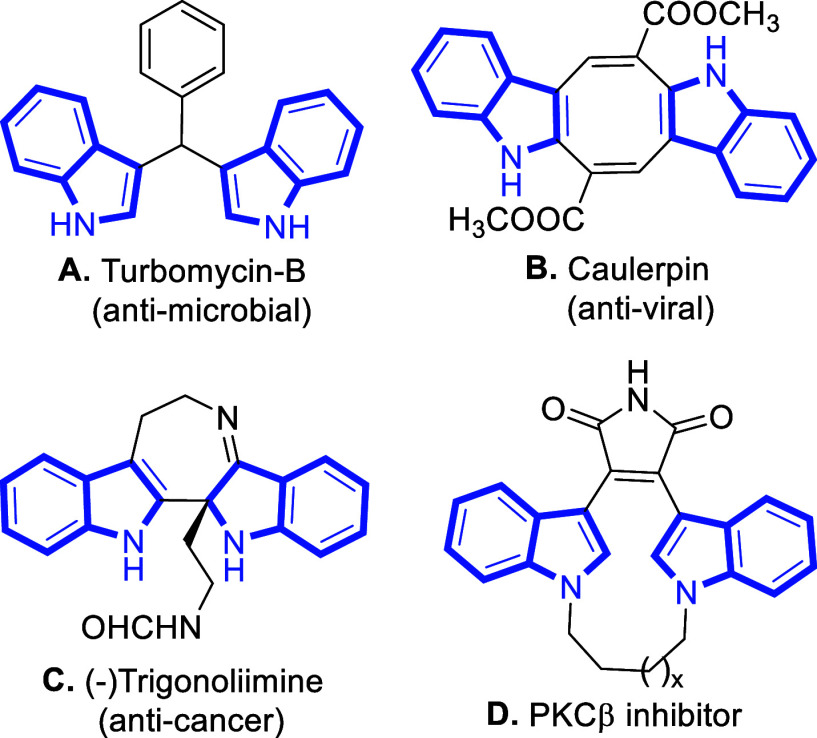
Representative examples of natural products and bioactive *bis*-indolylmethanes.

Although, macrocyclization is a time-consuming process, many research groups have geared their attention to explore highly effective and easy synthetic methods for the design of macrocycles for clinical and technological purposes.^10^ Literature reports^11^ illustrate a number of macrocyclization techniques, including dilution technique, synthetic strategies, and isolation and biological applications of such desirable structures. Scientific and technological challenges coupled with environmental considerations have prompted a search for a simple energy-efficient water-mediated synthesis for starting materials. The efficiency of macrocyclization varies on the size and structure of the macrocyclic core, as well as the structural preorganization of the linear substrates. Olefin metathesis reactions (RCM), coupling reactions catalyzed by transition metals, and IMDAR (intramolecular Diels–Alder reaction) are a few of the traditional macrocyclization techniques which involve C–C bond formation for cyclization. Diversity-Oriented Synthesis (DOS),^12^ which emerged in the early 2000s by Schreiber lab,^13^ is a powerful strategic solution for the construction of diverse macrocyclic libraries, utilizing modern synthetic methodology coupled with the essence of traditional techniques. Build/couple/pair (B/C/P) strategy^14^ is one such systematic, three-phase, and synthetic approach to generate diversity in the molecular structure. In the “build” phase, building blocks are synthesized that are then connected together intermolecularly in the “couple” phase. The final “pair” phase involves intramolecular functional group pairing. Enormous efforts have been devoted toward the synthesis of *bis*- and *tris*-indolylmethanes (BIMs and TIMs)^15^ due to their bioactive properties (Scheme 1A). However, there are no reports for analogues *bis*(aminoindolyl)­methanes, where the reaction takes place exclusively on the 3,3' position of sulfonamide without forming TIMs probably due to the competitive amino groups at the second position. Taking into consideration the previous work, we herein report an innovative build, couple, pair (B/C/P) method, metal catalyst-free protocol toward the synthesis of *bis*(aminoindolyl)­methanes from 2-sulfonamidoindoles and aromatic aldehydes. The LSF^16^ on indolic scaffolds are executed on “unprotected” N–H of *bis*(aminoindolyl)­methanes with dibromo-compounds in the presence of a catalytic amount of DBU to afford diindole-fused diazamacrocycles (Scheme 1B).

**Scheme 1:**
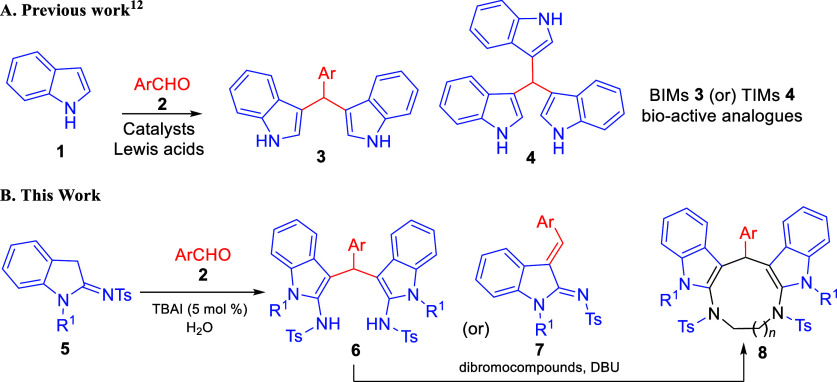


## 2 Results and Discussion

Initially, the linear precursor 2-sulfonamidoindoles **5** were built, which were coupled further with the reaction of an equimolar amount of benzaldehydes **2** in the presence of a catalytic amount of acid to study the synthesis of *bis*(indolyl)­methanes under different conditions. Toward this, the reaction of 2-sulfonamidoindole **5** and benzaldehyde **2** in the presence of a catalytic amount of *p*-TSA in dichloromethane was carried out at room temperature. A distinct new spot in TLC was observed, and product **6** was isolated using column chromatography and characterized as *bis*(indolyl)­methane based on proton and carbon NMR spectral analyses (Table 1, entry 1). The molecular weight of product **6** was also corroborated based on the HRMS. Encouraged by the above results, we began by studying the optimized parameters to determine the best reaction conditions screening solvents, equivalence of precursors and catalysts to improve the yield of product **6**. The reaction was tested under reflux conditions to provide expected *bis*(indolyl)­amine **6** in good yield. Changing the equivalence of **5** and **2** from 1:1 to 1:0.5 did not show much difference in the yield of the product (Table 1, entry 2). Screening the catalysts in the presence of strong Bronsted acids such as (±)-CSA, H_2_SO_4_, TfOH, and the metal catalyst, Cu­(OTf)_2_, did not give any product but the starting materials **5** and **2** were recovered (Table 1, entries 3,4,5 and 10). In the presence of Lewis acids, such as InCl_3_, In­(OTf)_3_, BF_3_·OEt_2_, and FeCl_3_ only 3-arylidineidolin-2-imine **7** are formed instead of *bis*(indolyl)­amine **6** (Scheme 1B). They were characterized based on the spectral data and corroborated with the reported literature.^17^ Varying the solvents to dichloroethane or chloroform, the yield decreased drastically to 10 and 30% (Table 1, entries 11 and 12). Toluene and 1,4-dioxane failed to give any product (Table 1, entries 13 and 14). The reaction was tested under solvent-free conditions by grinding reactants **5** and **2** in a mortar. It was pleasing to observe that this method also provided a good yield of the desired product (Table 1, entry 15). The fate of the reaction was tested for catalyst-free conditions in dichloromethane as well as in a H_2_O medium, and the desired product was not observed. Surprisingly, the reaction in the H_2_O medium in the presence of 5 mol % of TBAI under reflux conditions provided an excellent yield of the desired product (Table 1, entry 16). It was also observed that the TLC profile under these conditions was clean, indicating the absence of side reactions. Hence, the reaction of **5** and **2** in H_2_O in the presence of a catalytic amount of TBAI under reflux provided the green rout^18^ to obtain the desired product **6** and was taken to be the best optimized condition.

**Table 1:**
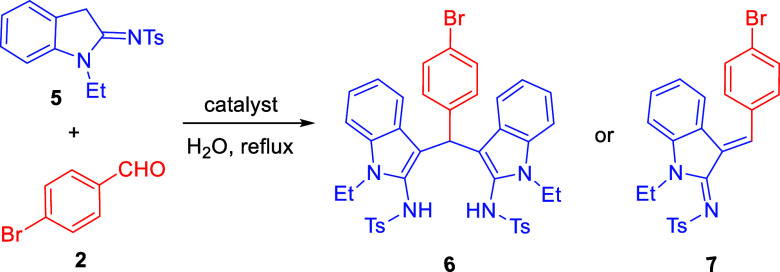
Survey of Reaction Conditions^a^

S. No	solvent	catalyst	equiv of 1:2	yield (%) **3** ^c^
1	DCM	*p*-TSA	1:1	70
2	DCM	*p*-TSA	1:0.5	65
3	DCM	(±)-CSA	1:1	nr^d^
4	DCM	H_2_SO_4_	1:1	nr^d^
5	DCM	TfOH	1:1	nr^d^
6	DCM	InCl_3_	1:1	30^e^
7	DCM	In(OTf)_3_	1:1	38^e^
8	DCM	BF_3_·OEt_2_	1:1	42^e^
9	DCM	FeCl_3_	1:1	10^e^
10	DCM	Cu(OTf)_2_	1:1	nr^d^
11	DCE	*p*-TSA	1:1	10
12	chloroform	*p*-TSA	1:1	30
13	toluene	*p*-TSA	1:1	nr^d^
14	1,4 dioxane	*p*-TSA	1:1	nr^d^
15	neat	*p*-TSA	1:1	60^f^
**16**	**H**_**2**_ **O**	**TBAI**	1:1	**72b**

a Entry 16 is the optimized reaction conditions for this transformation.

b Reaction conditions: All the reactions of **5** and **2** were carried out in 5 mol % of TBAI 1 h.

c Yield of the isolated product **6**.

d Nr = no reaction.

e Mono-substituted indole **7** formed.

f Grinding technique used.

Having optimized the standard reaction conditions and characterized the product, we moved on to survey the substrate scope of aldehydes to synthesize *bis*(indolyl)­amines (Scheme 2). The use of para- and meta- substituted benzaldehydes provided the corresponding *bis*(indolyl)­amines **6a**-**e** in good yield. Interestingly, the representative *bis*(indolyl)­amine **6c** was recrystallized using hexane/EtOAc as colorless crystals and the single-crystal X-ray analysis was performed^19^ to confirm the proposed product structure. The solid-state structure shows the presence of a C–H···π and two hydrogen bonding interactions. Heteroaromatic aldehydes, such as thiophene-3-carbaldehyde or furan-3-carbaldehyde, provided the corresponding *bis*(indolyl)­amines **6f** and **6g** in very good yield. The presence of either strong electron-donating or -withdrawing groups on aldehyde provided products **6h**–**j** in good yield.

**Scheme 2:**
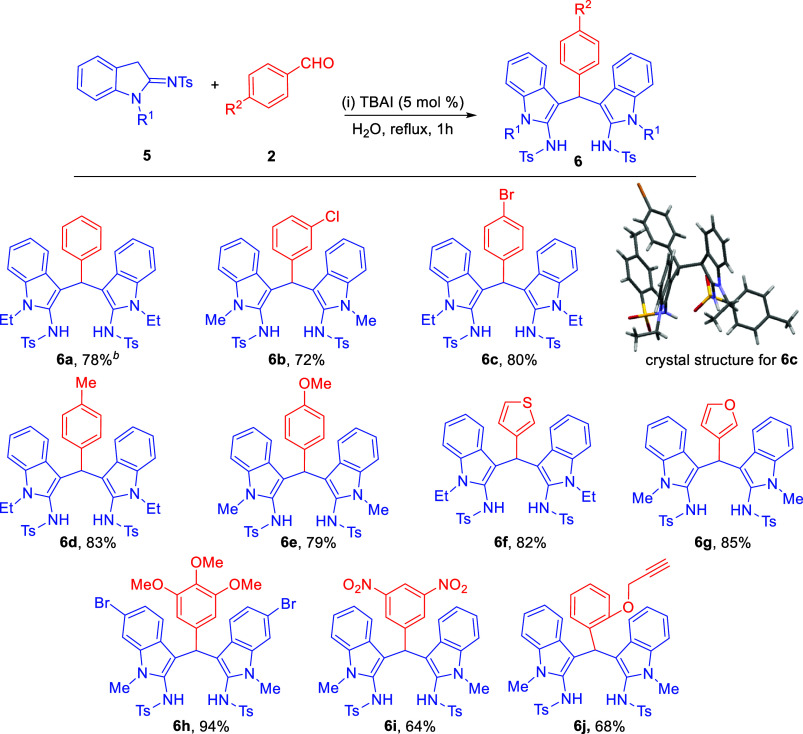
Substrate Scope for Aldehydes^a^ (**6a**–**j**)

a Reaction conditions: Reactions of 2-sulfonamidoindole **6** (1 equiv) and aromatic aldehyde **2** (1 equiv) in the presence of a catalytic amount of TBAI under reflux conditions.
b Yield of the isolated product.

Moving on to evaluate the substrate scope of 2-sulfonamidoindoles (Scheme 3), it was observed that *N*-alkylated indoles where *R* ^1^ as methyl **6k**, benzyl **6L**, or ethyl **6m** provided moderate to good yield of *bis*(indolyl)­amines **3**. 5-Methoxy, 5- or 6-bromosubstituted 2-sulfonamidoindoles provided a good yield of the corresponding products **6m**, **6n**, and **6o**. In our initial attempt to functionalize the dual NH protons of product **6**, a moderate yield of a single NH alkylated **6p** was obtained.

**Scheme 3:**
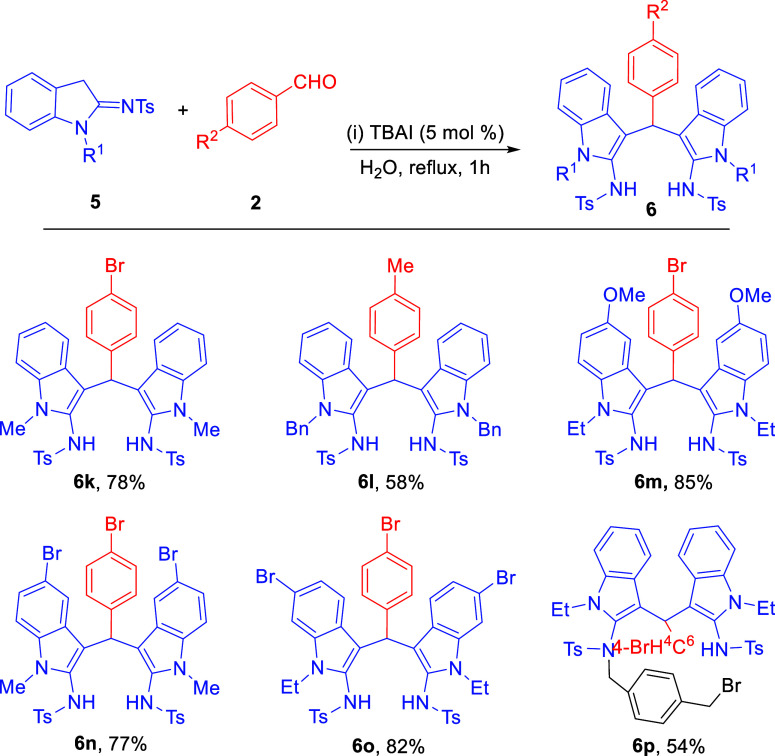
Substrate Scope for 2-Sulfonamidoindoles (**6k**–**p**)

a Reaction conditions: Reactions of 2-sulfonamidoindole **5** (1 equiv) and aldehyde **2** (1 equiv) in the presence of a catalytic amount of TBAI under reflux conditions.
b Yield of the isolated product **6**.

The use of other aldehydes, such as indole-3-carboxaldehyde, 1-naphthaldehyde, and 9-anthraldehyde (Scheme 4), under the optimized reaction conditions did not provide the expected *bis*(indolyl)­amines, instead gave a good yield of the corresponding amidines **7a**–**c** in line with the literature.^17^ This might be due to the steric hindrance caused by the aldehydes, which are comparatively bulkier in size. Unsubstituted sulfonamidoindole provided product **7d**. The reason could be due to the presence of a competitive NH which might prevent the azafulvene intermediate from reacting with the other molecule of sulfonamidoindole. The fluoro-substituted sulfonamido indoles as well as *para*-bromo benzyl alkylated sulfonamidoindoles provided amidines **7e**–**f** and **7g**, respectively, which can be attributed to the strong electron-withdrawing nature of fluorine groups present on the precursors. The use of aliphatic aldehydes did not provide expected product **6** or **7** under the optimized conditions.

**Scheme 4:**
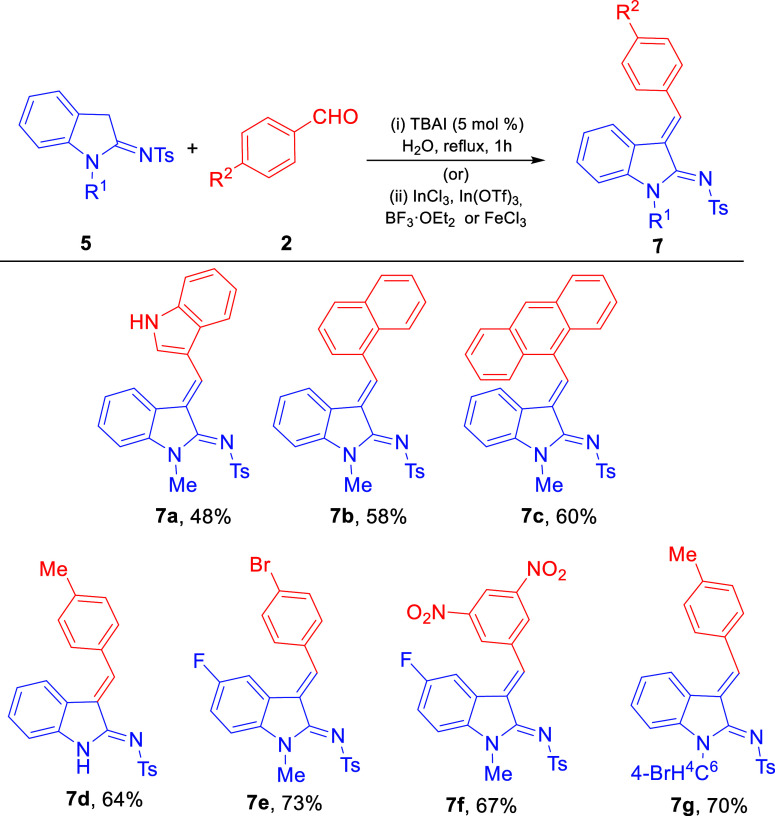
Substrate Scope for 2-Amine-3-arylindoles **(7a-g**)

a Reaction conditions: Reactions of 2-sulfonamidoindole **5** (1 equiv) and aldehyde **2** (1 equiv) in the presence of a catalytic amount of TBAI under reflux conditions.
b Yield of the isolated product **7**.

The ^1^H NMR spectra for products **6** after D_2_O exchange showed the disappearance of two NH protons, one around δ = 9.50–9.60 and the other among the aromatic peaks. Products **6** are potential molecules to functionalize further due to the presence of dual acidic NH protons. In continuation of our interest in the synthesis of macrocyclic compounds, it was planned to synthesize macrocycles by coupling the NH functionality via a *di*-alkylation method. Large-scale reactions were performed to synthesize *bis*(indolyl)­amines **6** for further applications. Encouraged by the isolation of single N–H alkylated product **6p**, the reaction was further continued in the presence of a catalytic amount of DBU in DCE at 50 °C with an appropriate dibromo-compound (Scheme 5). The reaction was stirred for about 3 to 4 h. On appearance of a new spot in TLC, the products were isolated through column chromatography and found to be the *bis*-alkylated product. Emphasis was given toward the dual–alkylation reaction of product **6** in the presence of a catalytic amount of DBU in DCE at 50 °C with an appropriate dibromo-compound to furnish the respective diindole-fused diazamacrocyclic system **8**. Dibromo-compounds of different chain lengths were used as spacers to provide the corresponding macrocycles **8a**–**e** having 10–15 numbered macrocyclic ring size.

**Scheme 5:**
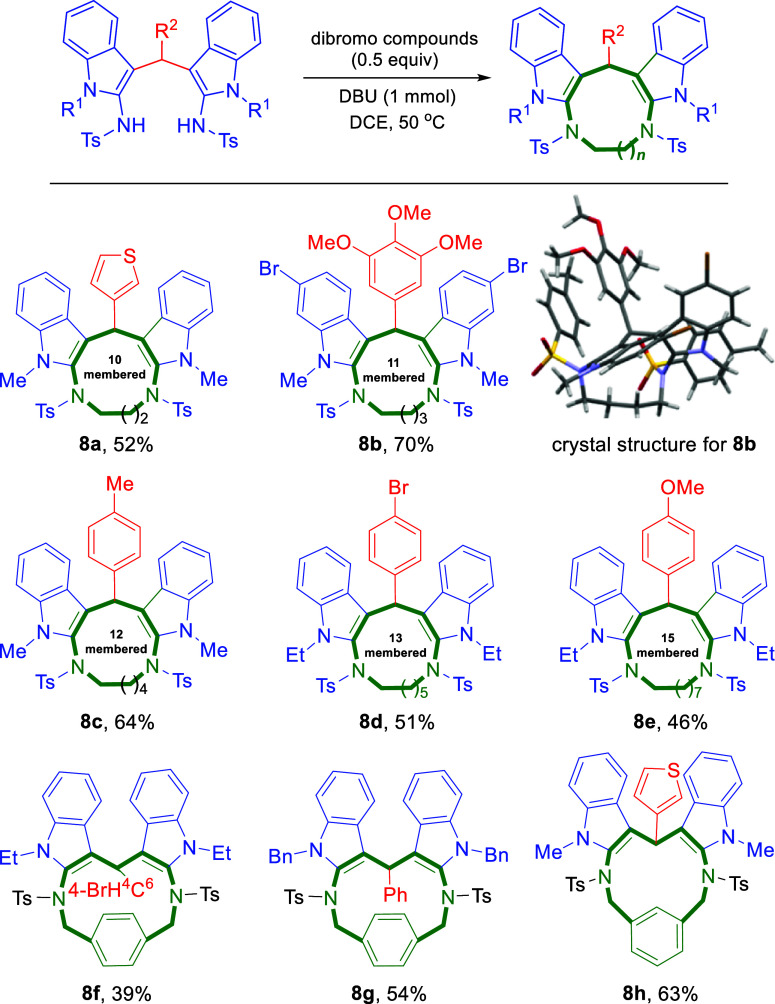
Substrate Scope for Macrocycles **(8a-h**)

a Reaction conditions: *bis*(indolyl)­amines **6** (1.0 equiv), alkyl halide/aryl halide (1.2 equiv), DBU (10 mol %) in DCE at 50 °C for 2 to 3 h.
b Yield of the isolated product **8**.

Aromatic dibromo compounds, such as 1,4-*bis*(bromomethyl)­benzene and 1,3-*bis*(bromomethyl)­benzene, also provided respective **8f**, **8g**, and **8h** macrocyclic products in a moderate yield. It was observed that the yield of product **8** decreased with an increase in the size of the macrocyclic ring. However, it was impossible to obtain the corresponding macrocyclic product **8**, as it was hard to synthesis *bis* indolyl product **6** from unsubstituted sulfonamidoindole. Although the synthesis product 6 was facile, like many other macrocyclization methods, this strategy had its limitations with regard to the substrate scope and % of yield.

The representative macrocyclic product **8b** was successfully recrystallized using hexane/EtOAc as colorless crystals and the single-crystal X-ray analysis was performed^20^ to confirm the structure of the macrocyclic product. The solid-state arrangement of product **8b** showed the presence of nine hydrogen bonding interactions.

In order to understand the above transformations and predict the possible pathway for the formation of product **6**, a few control experiments were examined. The reaction between 3-diazoindolin-2-imines **10** and aryl aldehydes **2** under optimized conditions provided 2-amino-3-arylindole **11** in line with the literature^21^ (Scheme 6, eq 1). Reaction of 2-sulfonamidoindoles 5 with ketones 13 under the optimized reaction conditions did not give desired product 6 (Scheme 6, eq 3).

**Scheme 6:**
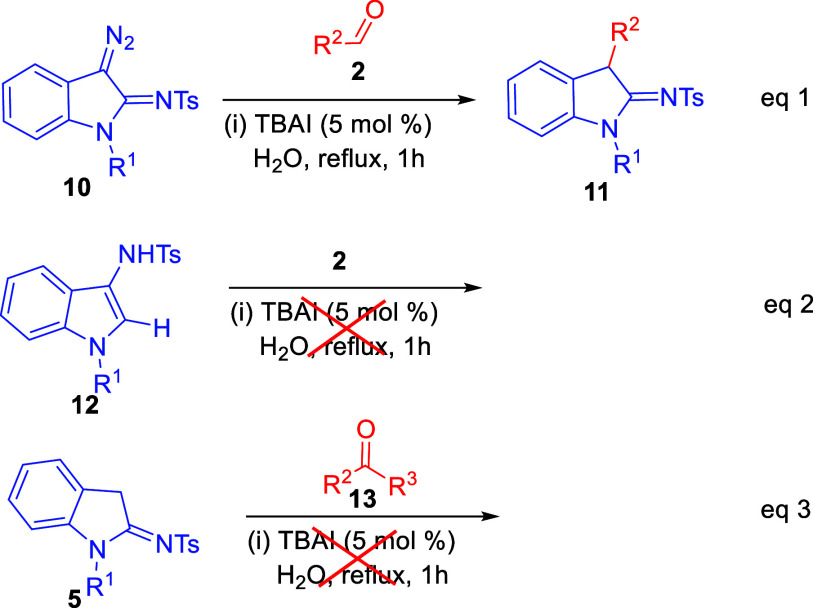
Control Experiments

Based on the experimental observations as well as from the previous reports^18,22^ a plausible mechanistic pathway for the formation of product 6 can be assumed. First, ^*n*^Bu_4_NI (TBAI) was oxidized to [^*n*^Bu_4_N]^+^[IO]^−^ in and through H_2_O.^23^ The enolized 2-sulfonamidoindole 5′ coordinated with [^*n*^Bu_4_N]^+^[IO]^−^ to give the activated 2-sulfonamidoindole intermediate **A**. When aldehydes having an electrophilic carbon center come in the proximity of intermediate **A**, it becomes even more electrophilic. Indole being an electron-rich heterocycle reacts with an electrophilic carbon center of the aldehyde through C-3 carbon resulting in the formation of 4-membered ring at the C3 position of azafulvene intermediate^24^ **C**, which is highly unstable. Due to the ring strain, the ring breaks to give enolated intermediated structure **D**, which on protonation loses a hydroxyl ion forming the enamine intermediate **E**, while [^*n*^Bu_4_N]^+^[IO]^−^ is released to be reoxidized. Finally, **E** undergoes oxidative coupling with another molecule of enolized **5′** to generate the expected (indolyl)­amine **6** via the loss of a water molecule or protonates to form amidines **7** only in few cases as shown in Scheme 4. Further, the duel acidic NH protons of compound **6** were successfully subjected to *di*-alkylation using dibromo-compounds in the presence of DBU in dichloromethane under reflux conditions to give diindole-fused diazamacrocyclic products **8** (Scheme 7).

**Scheme 7:**
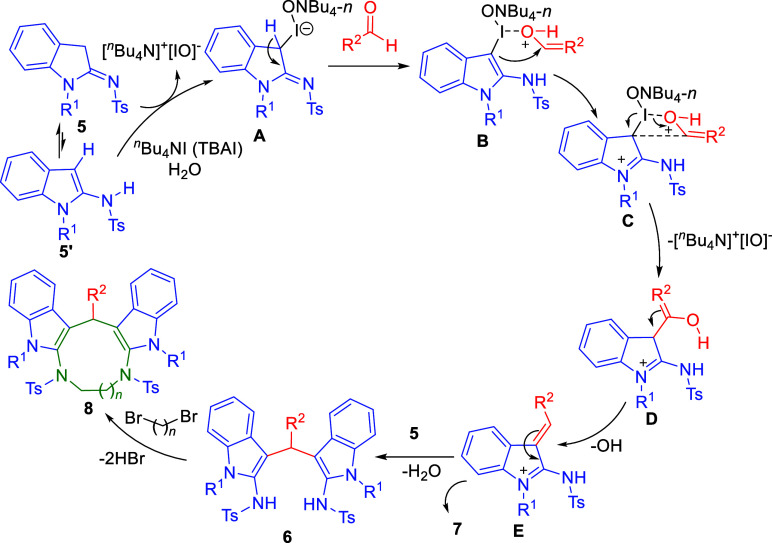
Plausible Mechanism for Products 6 and 8

## 3 Conclusion

In conclusion, we have demonstrated an environmentally benign metal-free B/C/P strategy in the synthesis of diazamacrocycles, where in the “build” phase, 2-sulfonamidoindoles building blocks are synthesized which are then connected together intermolecularly with aromatic aldehydes in the “couple” phase. The free N–H groups of *bis*(aminoindolyl)­methanes account for a significant reservoir for the final “pair” phase involving an intramolecular functional group pairing by the dibromo compounds via dialkylation. The diazamacrocycles presented in this work through a build, couple, pair (B/C/P) strategy are versatile and may find a leading platform for innovative transformations, and the methodology will find application in various fields. Further investigation and biological screening of these structures will also be of great interest.

### 3.1 General Information

Melting points were determined on a capillary melting point apparatus and were uncorrected. IR spectra were recorded using the ATR technique on a Bruker Alpha FT-IR spectrophotometer. All of the compounds were fully characterized. Proton nuclear magnetic resonance (^1^H NMR) spectra were recorded at 400 MHz using CDCl_3_ in ppm (δ) related to tetramethylsilane (δ = 0.00) as an internal standard and are reported as follows; chemical shift (ppm), multiplicity (br = broad, s = singlet, d = doublet, t = triplet, q = quartet dd = doublet of doublet, m = multiplet), coupling constant (*J*) values are given in parts per million and Hertz (Hz). Carbon-13 nuclear magnetic resonance (^13^C NMR) spectra were recorded at 100 MHz in CDCl_3_. Chemical shifts are reported in delta (δ) units and parts per million (ppm) relative to the center of the triplet at 77.7 ppm for CDCl_3_. Carbon types were determined from ^13^C NMR and DEPT experiments. The residual solvent signals were used as references, and the chemical shifts were converted to the TMS scale (CDCl_3_: δ_H_ = 7.26 ppm, δ_C_ = 77.7 ppm). High-resolution mass analyses were performed using an electrospray ionization (ESI) technique on a Bruker maXis mass spectrometer. All solvents were purified by distillation following the standard procedure. Thin-layer chromatography was performed on silica or alumina plates and components visualized by observation under iodine/UV light at 254 nm. Column chromatography was performed on silica gel (100–200 mesh). All the reactions were conducted in oven-dried glassware with magnetic stirring. Reagents, Indoles, NaN_3_, TsCl, aldehydes, dibromo spacer compounds, and other commercial chemicals were purchased from M/s Sigma-Aldrich, Alfa Aesar and used as provided.

### 3.2 General Procedure for Products 6 and 7

To an oven-dried RB flask were added aromatic aldehyde **2** (1 mmol) and 2-sulfonamidoindole **5** (1.2 mmol) were added. The mixture was stirred in the presence of 5 mol % of TBAI in H_2_O under reflux conditions for 1 h. The completion of the reaction was monitored using TLC. After the completion of the reaction, the product was extracted through dichloromethane, and the solvent was removed under reduced pressure. The organic phase was separated, and the aqueous layer was washed with ethyl acetate (10 mL). The concentration of combined organic layers under reduced pressure afforded the crude product, which was purified by column chromatography using silica gel (100–200 mesh, EtOAc/hexane 10:90) to furnish **6** and **7**. The crude solid obtained was directly recrystallized from methanol without chromatographic purification in the case of large-scale reactions to furnish products **6**.

### 3.3 Characterization Data for Products 6 and 7

#### 3.3.1 3,3′-(Phenylmethane-1,1-diyl)*bis*(*N*-tosyl-1-ethylindol-2-amine) (**6a**)

White solid; yield: 78%; *R*_*f*_ = 0.77 (Hexane/EtOAc = 9:1); mp 138–139 °C; IR (neat): ν_max_ = 3381, 3222, 2976, 1546, 1485, 1315, 1134, 986, 827 cm^–1^. ^1^H NMR (400 MHz, CDCl_3_): δ = 9.50 (s, 1H), 7.88 (d, *J* = 8.4 Hz, 2H), 7.53 (d, *J* = 8 Hz, 2H), 7.29–7.08 (m, 10H), 6.97 (t, *J* = 7.6 Hz, 1H), 6.87 (t, *J* = 7.6 Hz, 1H), 6.68 (d, *J* = 8 Hz, 2H), 6.40–7.34 (m, 2H), 6.15 (d, *J* = 7.2 Hz, 1H), 5.41–5.28 (m, 2H), 5.09–5.08 (m, 1H), 4.66–4.61 (m, 1H), 3.36–4.27 (m, 1H), 3.54–3.45 (m, 1H), 2.97–2.89 (m, 1H), 2.42 (s, 3H), 2.10 (s, 3H), 1.42 (t, *J* = 7.2 Hz, 3H), 0.07 (t, *J* = 7.2 Hz, 3H); ^13^C NMR (100 MHz, CDCl_3_): δ 170.3, 145.0, 143.4, 143.3, 139.1, 138.1, 136.5, 135.1, 131.0, 130.2, 130.1, 129.4, 129.0, 128.5, 127.4, 126.6, 126.3, 126.29, 123.6, 122.8, 122.7, 121.9, 120.5, 115.7, 113.2, 112.8, 104.9, 52.1, 45.6, 38.3, 36.2, 21.63, 21.59, 14.8, 10.6 ppm; HRMS (ESI) calcd for (C_41_H_40_N_4_O_4_S_2_) [M + H]^+^; 717.2569; found, 717.2595.

#### 3.3.2 3,3′-(3-Chlorophenylmethane-1,1-diyl)*bis*(*N*-tosyl-1-methylindol-2-amine) (**6b**)

White solid; yield: 72%; *R*_*f*_ = 0.72 (Hexane/EtOAc = 9:1); mp 142–143 °C; IR (neat): ν_max_ = 3230, 2928, 2855, 1737, 1602, 1562, 1508, 1462, 1342, 1158, 1087, 814, 749, 670 cm^–1^. ^1^H NMR (400 MHz, CDCl_3_): δ = 9.53 (s, 1H), 7.95 (d, *J* = 8.4 Hz, 2H), 7.56 (d, *J* = 8 Hz, 2H), 7.36–7.30 (m, 4H), 7.28–7.24 (m, 2H), 7.10–6.97 (m, 4H), 6.80 (d, *J* = 8.4 Hz, 2H), 6.47–6.41 (m, 2H), 6.33 (d, *J* = 7.2 Hz, 1H), 5.44 (d, *J* = 7.2 Hz, 1H), 5.35 (d, *J* = 8.4 Hz, 1H), 5.11 (d, *J* = 5.2 Hz, 1H), 3.92 (s, 3H), 2.67 (s, 3H), 2.47 (s, 3H), 2.23­(s, 3H); ^13^C NMR (100 MHz, CDCl_3_): δ 171.3, 144.5, 143.2, 143.0, 139.5, 138.9, 136.7, 135.1, 130. 8, 130.3, 129.4, 128.9, 127.4, 126.5, 125.2, 124.6, 123.7, 121.5, 120.2, 118.7, 109.7, 109.3, 104.0, 52.8, 45.5, 30.4, 27.9, 21.6, 21.6 ppm; HRMS (ESI) calcd for (C_39_H_35_ClN_4_O_4_S_2_) [M + H]^+^; 723.1866; found, 723.1877.

#### 3.3.3 3,3′-(4-Bromophenylmethane-1,1-diyl)*bis*(*N*-tosyl-1-ethylindol-2-amine) (**6c**)

White solid; yield: 80%; *R*_*f*_ = 0.65 (Hexane/EtOAc = 9:1); mp 188–189 °C; IR (neat): ν_max_ = 3317, 3060, 2924, 1553, 1484, 1334, 1161, 741 cm^–1^. ^1^H NMR (400 MHz, CDCl_3_): δ = 9.52 (s, 1H), 7.88 (d, *J* = 8.4 Hz, 2H), 7.54 (d, *J* = 8 Hz, 2H), 7.30–7.21 (m, 6H), 7.00–6.91 (m, 4H), 6.75 (d, *J* = 8.4 Hz, 2H), 6.41–6.34 (m, 2H), 6.27 (d, *J* = 7.2 Hz, 1H), 5.38–5.03 (m, 2H), 5.03 (d, *J* = 6 Hz, 1H), 4.70–4.61 (m, 1H), 4.35–4.26 (m, 1H), 3.54–3.45 (m, 1H), 2.98–2.89 (m, 1H), 2.42 (s, 3H), 2.19 (s, 3H), 1.42 (t, *J* = 7.2 Hz, 3H), 0.08 (t, *J* = 7.2 Hz, 3H); ^13^C NMR (100 MHz, CDCl_3_): δ = 170.5, 143.7, 143.2, 142.9, 139.7, 138.8, 136.6, 134.2, 130.7, 130.4, 129.8, 129.4, 129.3, 128.9, 128.8, 127.5, 126.5, 125.3, 125.0, 123.5, 121.8, 121.5, 120.2, 118.5, 109.8, 109.6, 104.6, 52.6, 45.8, 38.1, 36.0, 21.6, 21.6, 14.8, 10.4 ppm; HRMS (ESI) calcd for (C_41_H_39_BrN_4_O_4_S_2_) [M + H]^+^; 795.1674; found, 795.1674.

#### 3.3.4 3,3′-(Tolylmethane-1,1-diyl)*bis*(*N*-tosyl-1-ethylindol-2-amine) (**6d**)

White solid; yield: 83%; *R*_*f*_ = 0.78 (Hexane/EtOAc = 9:1); mp 140–141 °C; IR (neat): ν_max_ = 3393, 3324, 2924, 1553, 1489, 1451, 1316, 736 cm^–1^. ^1^H NMR (400 MHz, CDCl_3_): δ = 9.50 (s, 1H), 7.89 (d, *J* = 8.4 Hz, 2H), 7.53 (d, *J* = 8 Hz, 2H), 7.29–7.18 (m, 5H), 6.95–6.87 (m, 5H), 6.68 (d, *J* = 8.4 Hz, 2H), 6.39–6.23 (m, 3H), 5.40–5.30 (m, 2H), 5.03 (d, *J* = 6 Hz, 1H), 4.70–4.61 (m, 1H), 4.35–4.26 (m, 1H), 3.53–3.44 (m, 1H), 2.97–2.87 (m, 1H), 2.42–2.40­(m, 6H), 2.10 (s, 3H), 1.42 (t, *J* = 7.2 Hz, 3H), 0.08 (t, *J* = 7.2 Hz, 3H); ^13^C NMR (100 MHz, CDCl_3_): δ 170.9, 143.6, 142.8, 142.7, 139.9, 139.5, 136.5, 136.4, 135.5, 134.2, 130.1, 129.3, 128.9, 128.5, 128.3, 127.4, 126.4, 126.2, 125.5, 125.3, 123.4, 122.2, 121.3, 118.2, 109.5, 109.4, 105.5, 52.9, 46.0, 38.0, 35.9, 21.6, 21.2, 14.8, 10.5 ppm; HRMS (ESI) calcd for (C_42_H_42_N_4_O_4_S_2_) [M + H]^+^; 731.2726; found, 731.2729.

#### 3.3.5 3,3′-(4-Methoxyphenylmethane-1,1-diyl)*bis*(*N*-tosyl-1-ethylindol-2-amine) (**6e**)

White solid; yield: 79%; *R*_*f*_ = 0.67 (Hexane/EtOAc = 9:1); mp 145–146 °C; IR (neat): ν_max_ = 3375, 3064, 2925, 1539, 1486, 1311, 741 cm^–1^. ^1^H NMR (400 MHz, CDCl_3_): δ = 9.53 (s, 1H), 7.57 (d, *J* = 8 Hz, 2H), 7.33 (d, *J* = 8 Hz, 2H), 7.25–7.22 (m, 2H), 7.01 (d, *J* = 8 Hz, 3H), 6.97–6.94 (m, 1H), 6.79–6.72 (m, 4H), 6.45–6.40 (m, 2H), 6.32 (d, *J* = 8 Hz, 3H), 5.45–5.37 (m, 2H), 5.09 (d, *J* = 8 Hz, 3H), 3.91–3.90 (m, 6H), 2.65 (s, 3H), 2.47 (s, 3H), 2.17 (s, 3H); ^13^C NMR (100 MHz, CDCl_3_): δ = 171.6, 158.0, 144.5, 142.9, 139.7, 136.6, 135.1, 131.8, 130.1, 129.4, 128.9, 128.7, 127.3, 126.7, 126.2, 125.4, 124.9, 123.5, 121.9, 121.3, 118.5, 114.1, 112.9, 109.5, 109.2, 104.9, 55.3, 55.2, 45.4, 36.2, 30.3, 27.9, 21.6 ppm; HRMS (ESI) calcd for (C_40_H_38_N_4_O_5_S_2_) [M + H]^+^; 719.2362; found, 719.2356.

#### 3.3.6 3,3′-(Thiophen-3-ylmethane-1,1-diyl)*bis*(*N*-tosyl-1-ethylindol-2-amine) (**6f**)

White solid; yield: 82%; *R*_*f*_ = 0.67 (Hexane/EtOAc = 9:1); mp 130–131 °C; IR (neat): ν_max_ = 3385, 3236, 2925, 1553, 1497, 1320, 802 cm^–1^. ^1^H NMR (400 MHz, CDCl_3_): δ = 9.52 (s, 1H), 7.89 (d, *J* = 8.4 Hz, 2H), 7.54 (d, *J* = 8 Hz, 2H), 7.30–7.21 (m, 5H), 7.00–6.91 (m, 4H), 6.75 (d, *J* = 8.4 Hz, 2H), 6.41–6.34 (m, 2H), 6.27 (d, *J* = 7.2 Hz, 1H), 5.38–5.24 (m, 2H), 5.03 (d, *J* = 5.6 Hz, 1H), 4.70–4.61 (m, 1H), 4.35–4.26 (m, 1H), 3.54–3.45 (m, 1H), 2.98–2.89 (m, 1H), 2.42 (s, 3H), 2.19 (s, 3H), 1.42 (t, *J* = 7.2 Hz, 3H), 0.08 (t, *J* = 7.2 Hz, 3H); ^13^C NMR (100 MHz, CDCl_3_): δ 170.5, 143.7, 143.2, 142.9, 139.7, 138.8, 136.6, 134.2, 130.7, 130.4, 129.8, 129.4, 129.3, 128.9, 128.8, 127.5, 126.4, 125.3, 125.0, 123.5, 121.8, 121.5, 120.2, 118.5, 109.7, 109.6, 104.6, 52.6, 45.8, 38.1, 36.0, 21.6, 21.5, 14.8, 10.4 ppm; HRMS (ESI) calcd for (C_39_H_38_N_4_O_5_S_2_) [M + H]^+^; 723.2133; found, 723.2148.

#### 3.3.7 3,3′-(Furan-3-ylmethane-1,1-diyl)*bis*(*N*-tosyl-1-methylindol-2-amine) (**6g**)

White solid; yield: 85%; *R*_*f*_ = 0.67 (Hexane/EtOAc = 9:1); mp 127–129 °C; IR (neat): ν_max_ = 3313, 2923, 1637, 1574, 1280, 1138, 1087, 820, 671 cm^–1^. ^1^H NMR (400 MHz, CDCl_3_): δ = 9.54 (s, 1H), 7.95 (d, *J* = 8.4 Hz, 2H), 7.56 (d, *J* = 8 Hz, 2H), 7.35–7.24 (m, 3H), 7.10–7.00 (m, 6H), 6.79 (d, *J* = 8.4 Hz, 2H), 6.47–6.41 (m, 2H), 6.31 (d, *J* = 7.2 Hz, 1H), 5.44–5.33 (m, 2H), 5.10 (d, *J* = 8 Hz, 1H), 3.91­(s, 3H), 2.67 (s, 3H), 2.47 (s, 3H), 2.23 (s, 3H); ^13^C NMR (100 MHz, CDCl_3_): δ = 169.7, 143.2, 143.3, 143.2, 143.1, 139.0, 136.4, 133.6, 132.9, 131.2, 130.8, 129.4, 129.0, 128.6, 127.4, 127.1, 126.7, 126.5, 125.6, 124.8, 124.1, 123.9, 116.9, 112.4, 110.8, 110.5, 104.4, 54.1, 42.8, 30.6, 28.0, 21.7, 21.6 ppm; HRMS (ESI) calcd for (C_37_H_34_N_4_O_5_S_2_) [M + H]^+^; 679.2049, found:679.2052.

#### 3.3.8 3,3′-(3,4,5-Trimethoxyphenylmethane-1,1-diyl)*bis*(*N*-tosyl-1-methyl-indol-2-amine) (**6h**)

White solid; yield: 94%; *R*_*f*_ = 0.80 (Hexane/EtOAc = 9:1); mp 159–160 °C; IR (neat): ν_max_ = 3244, 3060, 2925, 1562, 1485, 1275, 1150, 1085, 853, 671 cm^–1^. ^1^H NMR (400 MHz, CDCl_3_): δ = 9.56 (s, 1H), 7.90 (d, *J* = 8 Hz, 2H), 7.51–7.47 (m = 3H), 7.31–7.27 (m, 2H), 7.10 (s, 2H), 6.80 (d, *J* = 8 Hz, 2H), 6.39–6.20 (m, 3H), 6.19 (s, 1H), 5.66 (s, 1H), 5.27 (d, *J*_1_ = 5.6 Hz, 1H). 5.02 (d, *J* = 5.6 Hz, 1H), 3.97 (s, 3H), 3.90 (s, 3H), 3.72 (s, 6H), 2.63 (s, 3H), 2.43 (s, 3H), 2.20 (s, 3H); ^13^C NMR (100 MHz, CDCl_3_): δ 170.5, 152.6, 143.6, 143.4, 143.2, 139.1, 137.0, 136.5, 134.9, 133.7, 132.8, 131.4, 131.0, 129.4, 129.0, 128.5, 127.5, 127.0, 126.0, 125.0, 125.0, 124.2, 116.7, 112.3, 111.1, 110.8, 105.7, 104.2, 61.3, 56.2, 53.7, 46.2, 30.7, 28.0, 21.6, 21.2 ppm; HRMS (ESI) calcd for (C_42_H_40_Br_2_N_4_O_7_S_2_) [M + H]^+^; 937.0763; found, 937.0775.

#### 3.3.9 3,3′-(3,5-Dinitrophenylmethane-1,1-diyl)*bis*(*N*-tosyl-1-methylindol-2-amine) (**6i**)

Orange solid; yield: 64%; *R*_*f*_ = 0.30 (Hexane/EtOAc = 3:1); mp 170–182 °C; IR (neat): ν_max_ = 3224, 2928, 2856, 1602, 1462, 1342, 1158, 814, 745, 670 cm^–1^. ^1^H NMR (400 MHz, CDCl_3_): δ = 9.22 (s, 1H), 9.03 (s, 1H), 8.02–7.92 (m, 4H), 7.84 (s, 1H), 7.69–7.27 (m, 1H), 7.51–7.47 (m, 1H), 7.41–7.34 (m, 5H), 7.29–7.25 (m, 1H), 7.14–6.99 (m, 3H), 6.90 (t, *J* = 7.2 Hz, 1H), 6.24 (d, *J* = 7.2 Hz, 1H), 3.88 (s, 2H), 3.44 (s, 4H), 2.49 (s, 4H), 2.41 (s, 2H); ^13^C NMR (100 MHz, CDCl_3_): δ 171.3, 144.5, 143.2, 143.0, 139.5, 138.8, 136.6, 135.1, 130.8, 130.3, 130.2, 129.6, 129.4, 128.9, 127.4, 126.5, 125.2, 124.6, 123.7, 121.5, 120.2, 118.7, 109.7, 109.3, 104.0 52.7, 45.5, 30.4, 27.9, 21.6, 21.5 ppm; HRMS (ESI) calcd for (C_39_H_34_N_6_O_8_S_2_) [M + H]^+^; 779.1958; found, 779.1970.

#### 3.3.10 3,3′-(2-(Prop-2-yn-1-yloxy)­phenylmethane-1,1-diyl)*bis*(*N*-tosyl-1-methylindol-2-amine) (**6j**)

White solid; yield: 68%; *R*_*f*_ = 0.74 (Hexane/EtOAc = 9:1); mp 123–124 °C; IR (neat): ν_max_ = 3213, 3072, 2928, 1547, 1501, 1313, 1220, 829 cm^–1^. ^1^H NMR (400 MHz, CDCl_3_): δ = 9.76 (s, 1H), 7.95 (d, *J* = 8.4 Hz, 2H), 7.49 (d, *J* = 8.4 Hz, 2H), 7.34–7.22 (m, 5H), 7.08–7.03 (m, 2H), 7.02–6.90 (m, 2H), 6.71 (d, *J* = 8 Hz, 2H), 6.47–6.40 (m, 2H), 6.10 (dd, *J*_1_ = 27.2 Hz, *J*_2_ = 7.2 Hz, 2H), 5.30–5.26 (m, 2H), 5.04 (dd, *J*_1_ = 16 Hz, *J*_2_ = 5.4 Hz, 1H), 4.79 (dd, *J*_1_ = 16 Hz, *J*_2_ = 8.8 Hz, 1H), 3.90 (s, 3H), 2.58 (s, 3H), 2.55 (t, *J* = 2.4 Hz, 2H), 2.46 (s, 3H), 2.16 (s, 3H); ^13^C NMR (100 MHz, CDCl_3_): δ 172.7, 154.8, 144.9, 142.5, 142.1, 140.3, 136.6, 135.2, 131.0, 130.8, 129.9, 129.3, 129.2, 129.1, 128.8, 128.4, 128.2, 127.6, 127.2, 126.5, 126.3, 125.4, 125.2, 123.3, 122.2, 121.3, 120.6, 118.3, 111.4, 109.6, 109.1, 105.5, 79.4, 75.5, 55. 2, 50.1, 42.3, 30.5, 27.7, 21.8, 21.6 ppm; HRMS (ESI) calcd for (C_42_H_38_N_4_O_5_S_2_) [M + H]^+^; 743.2362; found, 743.2352.

#### 3.3.11 3,3′-(4-Bromophenylmethane-1,1-diyl)*bis*(*N*-tosyl-1-methylindol-2-amine) (**6k**)

White solid; yield: 78%; *R*_*f*_ = 0.69 (Hexane/EtOAc = 9:1); mp 177–178 °C; IR (neat): ν_max_ = 3068, 2923, 2857, 1587, 1488, 1150, 1021, 815, 755, 673 cm^–1^. ^1^H NMR (400 MHz, CDCl_3_): δ = 9.50 (s, 1H), 7.90 (d, *J* = 8 Hz, 2H), 7.52 (d, *J* = 8 Hz, 2H), 7.31–7.29 (m, 3H), 7.23–7.20 (m, 2H), 7.02–6.93 (m, 4H), 6.75 (d, *J* = 8 Hz, 2H), 6.43–6.40 (m, 2H), 6.28 (d, *J* = 7.6 Hz, 1H), 5.37 (d, *J* = 5.6 Hz, 1H), 5.31–5.29 (m, 2H), 5.10 (d, *J* = 5.2 Hz, 1H), 3.90 (s, 3H), 2.62 (s, 3H), 2.43 (s, 3H), 2.20 (s, 3H); ^13^C NMR (100 MHz, CDCl_3_): δ 171.3, 144.5, 143.2, 143.0, 139.5, 138.8, 136.6, 135.1, 130.8, 130.3, 130.2, 129.6, 129.4, 128.9, 127.4, 126.5, 125.2, 124.6, 123.7, 121.5, 120.2, 118.7, 109.7, 109.3, 104.0, 52.7, 45.5, 30.4, 27.9, 21.6, 21.5 ppm; HRMS (ESI) calcd for (C_39_H_35_BrN_4_O_4_S_2_) [M + H]^+^; 767.1361; found, 767.1363.

#### 3.3.12 3,3′-(4-Tolylphenylmethane-1,1-diyl)*bis*(*N*-tosyl-1-benzylindol-2-amine) (**6L**)

White solid; yield: 57%; *R*_*f*_ = 0.67 (Hexane/EtOAc = 9:1); mp 139–141 °C; IR (neat): ν_max_ = 3144, 2974, 1556, 1155, 1061, 814, 739, 674, 830 cm^–1^. ^1^H NMR (400 MHz, CDCl_3_): δ = 9.73 (s, 1H), 7.87 (d, *J* = 8.4 Hz, 2H), 7.87 (d, *J* = 8.4 Hz, 2H), 7.32–7.30 (m, 2H), 7.23–7.15 (m, 1H), 7.14–7.12 (m, 7H), 7.11–6.98 (m, 6H), 6.97–6.74 (m, 5H), 6.41–6.23 (m, 6H), 6.10 (d, *J* = 16.4 Hz, 1H), 5.56–5.44 (m, 2H), 5.35–5.23 (m, 2H), 2.27 (d, *J* = 4.8 Hz, 2H), 2.48–2.45 (m, 6H), 2.16 (s, 3H); ^13^C NMR (100 MHz, CDCl_3_): δ = 171.3, 144.5, 143.3, 143.0, 139.5, 138.9, 136.6, 135.1, 130.8, 130.3, 129.4, 129.0, 127.4, 126.5, 125.1, 124.6, 123.7, 121.5, 120.2, 118.7, 109.7, 109.4, 104.1, 52.8, 45.5, 30.4, 28.0, 21.7, 21.6 ppm; HRMS (ESI) calcd for (C_32_H_46_N_4_O_4_S_2_) [M + H]^+^; 855.3039; found, 855.3051.

#### 3.3.13 3,3′-(4-Bromophenylmethane-1,1-diyl)*bis*(*N*-tosyl-1-ethyl-5-methoxyindol-2-amine) (**6m**)

White solid; yield: 85%; *R*_*f*_ = 0.66 (Hexane/EtOAc = 9:1); mp 169–170 °C; IR (neat): ν_max_ = 3374, 2925, 1567, 1252, 1077, 809, 730, 682 cm^–1^. ^1^H NMR (400 MHz, CDCl_3_): δ = 9.52 (s, 1H), 7.99 (d, *J* = 8 Hz, 2H), 7.73 (d, *J* = 8 Hz, 2H), 7.39–7.37 (m, 2H), 7.29 (d, *J* = 5.2 Hz, 1H), 7.21 (d, *J* = 9.2 Hz, 1H), 6.97–6.90 (m, 3H), 6.84 (dd, *J*_1_ = 8 Hz, *J*_2_ = 2.4 Hz, 2H), 6.79–6.77 (m, 1H), 6.49–6.47 (m, 1H), 6.31–6.30 (m, 1H), 5.40–5.39 (m, 2H), 5.32 (dd, *J*_1_ = 5.2 Hz, *J*_2_ = 5.6 Hz, 1H), 4.78–4.69 (m, 1H), 4.40–4.31 (m, 1H), 3.69 (s, 3H), 3.65–3.58 (m, 1H), 3.50 (s, 3H), 3.11–3.03 (m, 1H), 2.52 (s, 3H), 2.32 (s, 3H), 1.50 (t, *J* = 7.2 Hz, 3H), 0.29 (t, *J* = 7.2 Hz, 3H); ^13^C NMR (100 MHz, CDCl_3_): δ 169.3, 156.5, 152.8, 143.8, 143.1, 142.7, 140.0, 137.1, 136.6, 131.4, 129.4, 129.4, 129.3, 129.0, 127.6, 127.1, 126.4, 126.0, 125.0, 125.5, 114.4, 112.6, 111.8, 110.3, 109.7, 105.3, 104.2, 55.6, 55.4, 54.3, 43.0, 38.1, 36.1, 21.7, 21.5, 14. 9, 10.7 ppm; HRMS (ESI) calcd for (C_44_H_43_BrN_4_O_6_S_3_) [M + H]^+^; 855.1886; found, 855.1897.

#### 3.3.14 3,3′-(4-Bromophenylmethane-1,1-diyl)*bis*(*N*-tosyl-1-methyl-5-bromoindol-2-amine) (**6n**)

White solid; yield: 77%; *R*_*f*_ = 0.68 (Hexane/EtOAc = 9:1); mp 189–190 °C; IR (neat): ν_max_ = 3056, 2931, 1561, 1276, 1145, 1087, 813, 750, 679 cm^–1^. ^1^H NMR (400 MHz, CDCl_3_): δ = 9.46 (s, 1H), 7.94 (d, *J* = 8.4 Hz, 2H), 7.62 (d, *J* = 8 Hz, 2H), 7.51–7.49 (m, 2H), 7.35–7.26 (m, 3H), 7.16–7.10 (m, 2H), 6.91–6.88 (m, 3H), 6.61 (s, 1H), 6. 46 (s, 1H), 6.36 (d, *J* = 8.4 Hz, 1H), 5.75 (s, 1H), 5.30 (d, *J* = 7.2 Hz, 1H), 5.13 (s, 1H), 3.88 (s, 3H), 2.67 (s, 3H), 2.47 (s, 3H), 2.26 (s, 3H); ^13^C NMR (100 MHz, CDCl_3_): δ = 169.7, 143.2, 143.3, 143.2, 143.1, 139.0, 136.4, 133.6, 132.9, 131.2, 130.8, 129.4, 129.0, 128.6, 127.4, 127.1, 126.7, 126.5, 125.6, 124.8, 124.1, 123.9, 116.9, 112.4, 110.8, 110.5, 104.4, 54.1, 42.8, 30.6, 28.0, 21.7, 21.6 ppm; HRMS (ESI) calcd for (C_39_H_33_Br_3_N_4_O_4_S_2_) [M + H]^+^; 924.9551; found, 924.9541.

#### 3.3.15 3,3′-(4-Bromophenylmethane-1,1-diyl)*bis*(*N*-tosyl-1-ethyl-6-bromoindol-2-amine) (**6o**)

White solid; yield: 82%; *R*_*f*_ = 0.71 (Hexane/EtOAc = 9:1); mp 188–189 °C; IR (neat): ν_max_ = 3054, 2925, 2856, 1493, 1224, 1018, 735 cm^–1^. ^1^H NMR (400 MHz, CDCl_3_): δ = 9.48 (s, 1H), 7.87 (d, *J* = 8.4 Hz, 2H), 7.51 (d, *J* = 8 Hz, 2H), 7.41–7.40 (m, 1H), 7.31–7.26 (m, 5H), 7.07–7.05 (m, 1H), 6.90–6.89 (m, 2H), 6.75 (d, *J* = 6 Hz, 2H), 6.62–6.60 (m, 2H), 6.08 (d, *J* = 8 Hz, 1H), 5.29–5.19 (m, 2H), 4.99 (d, *J* = 5.6 Hz, 1H), 4.68–4.59 (m, 1H), 4.28–4.19 (m, 1H), 3.51–3.43 (m, 1H), 2.97–2.88 (m, 1H), 2.42 (s, 3H), 2.19 (s, 3H), 1.40 (t, *J* = 7.2 Hz, 3H), 0.15 (t, *J* = 7.2 Hz, 3H); ^13^C NMR (100 MHz, CDCl_3_): δ 170.3, 145.0, 143.4, 143.3, 139.1, 138.1, 136.5, 135.1, 131.0, 130.2, 130.1, 129.4, 129.0, 128.5, 127.4, 126.5, 126.3, 126.2, 123.6, 122.8, 122.7, 121.9, 120.6, 115.7, 113.2, 112.8, 104.9, 52.1, 45.6, 38.3, 36.2, 21.6, 21.6, 14.8, 10.6 ppm; HRMS (ESI) calcd for (C_41_H_37_Br_3_N_4_O_4_S_2_) [M + H]^+^; 952.9864; found, 952.9877.

#### 3.3.16 *N*-(4-(Bromomethyl)­benzyl)-*N*-(1-ethyl-3-((1-ethyl-2-((4-tolyl)­sulfonamido)-1*H*-indol-3-yl)­(*p*-bromophenyl)­methyl)-1*H*-indol-2-yl)-4-tolylsulfonamide (**6p**)

White solid; yield: 54%; *R*_*f*_ = 0.66 (Hexane/EtOAc = 3:1); mp 172–174 °C; IR (neat): ν_max_ = 3241, 2929, 1562, 1486, 1212, 1163, 1032, 735 cm^–1^. ^1^H NMR (400 MHz, CDCl_3_): δ = 7.79 (d, *J* = 8 Hz, 3H), 7.54 (d, *J* = 8 Hz, 3H), 7.24–6.17 (m, 8H), 6.08–7.02 (m, 7H), 6.89–6.86 (m, 5H), 6.35 (m, 1H), 4.01 (s, 4H), 4.00 (q, *J* = 7.2 Hz, 2H), 3.83 (q, *J* = 7.2 Hz, 2H), 2.32–2.27 (m, 6H), 1.32 (t, *J* = 7.2 Hz, 3H), 1.18 (t, *J* = 7.2 Hz, 3H); ^13^C NMR (100 MHz, CDCl_3_): δ = 169.2, 143.4, 143.0, 142.7, 139.5, 136.8, 134.3, 129.4, 128.1, 127.5, 126.7, 124.6, 124.5 123.6, 122.0, 119.7, 117.8, 110.9, 109.5, 109. 41.1, 37.0, 36.2, 21.5, 15.4, 12.1 ppm; HRMS (ESI) calcd for (C_49_H_46_Br_2_N_4_O_4_S_2_) [M + H]^+^; 979.1385; found, 979.1389.

#### 3.3.17 (3*E*)-*N*-Tosyl-3-[(indolyl)­methylidene]-2,3-dihydro-1-methylindol-2-imine (**7a**)

Orange solid; yield: 48%; *R*_*f*_ = 0.68 (Hexane/EtOAc = 3:1); mp 133–134 °C; IR (neat): ν_max_ = 3311, 3055, 2924, 1572, 1462, 1276, 1137, 1085, 818 cm^–1^. ^1^H NMR (400 MHz, CDCl_3_): δ = 8.16 (s, 1H), 7.59–7.57 (m, 4H), 7.48–7.44 (m, 1H), 7.38–7.16 (m, 8H), 6.92 (s, 1H), 4.02 (s, 3H), 2.37 (s, 3H); ^13^C NMR (100 MHz, CDCl_3_): δ = 187.6, 163.2, 161.7, 160.5, 158.1, 153.6, 143.0, 139.7, 139.6, 139.3, 129.2, 127.1, 126.5, 126.0, 125.9, 125.4, 117.9, 117.1, 116.9, 111.7, 111.5, 111.0, 110.9, 110.1, 83.6, 29.5, 21.5 ppm; HRMS (ESI) calcd for (C_25_H_21_N_3_O_2_S) [M + H]^+^; 428.1432; found, 428.1439.

#### 3.3.18 (3*E*)-*N*-Tosyl-3-[(naphthyl)­methylidene]-2,3-dihydro-1-methylindol-2-imine (**7b**)

Yellow solid; yield: 58%; *R*_*f*_ = 0.77 (Hexane/EtOAc = 9:1); mp 132–135 °C; IR (neat): ν_max_ = 3233, 3059, 2926, 1561, 1484, 1272, 1149, 1084, 809, 768, 671 cm^–1^. ^1^H NMR (400 MHz, CDCl_3_): δ = 8.96 (s, 1H), 8.16 (s, 1H), 8.03 (d, *J* = 8 Hz, 2H), 7.98–7.90 (m, 2H), 7.76–7.73 (m, 1H), 7.64–7.56 (m, 3H), 7.37–7.31 (m, 4H), 7.00–6.90 (m, 2H), 3.58 (s, 3H), 2.48 (s, 3H); ^13^C NMR (100 MHz, CDCl_3_): δ = 164.2, 151.6, 144.6, 141.3, 135.7, 135.4, 135.2, 129.7, 127.7, 127.2, 124.6, 124.4, 123.5, 121.4, 119.2, 118.8, 110.6, 110.1, 91.5, 31.0, 21.6 ppm; HRMS (ESI) calcd for (C_27_H_22_N_2_O_2_S) [M + H]^+^; 439.1480. found: 439.1477.

#### 3.3.19 (3*E*)-*N*-Tosyl-3-[(anthryl)­methylidene]-2,3-dihydro-1-methylindol-2-imine (**7c**)

Red solid; yield: 60%; *R*_*f*_ = 0.76 (Hexane/EtOAc = 9:1); mp 130–131 °C; IR (neat): ν_max_ = 3057, 2928, 1556, 1467, 1272, 1084, 809, 744, 675 cm^–1^. ^1^H NMR (400 MHz, CDCl_3_): δ = 9.77 (s, 1H), 8.63 (s, 1H), 8.14–8.06 (m, 6H), 7.57–7.48 (m, 4H), 7.39–7.36 (m, 3H), 6.89–6.85 (m, 2H), 5.57–5.55 (m, 1H), 3.55 (s, 3H), 2.49 (s, 3H); ^13^C NMR (100 MHz, CDCl_3_): δ = 174.2, 154.6, 144.9, 135.4, 135.3, 135.0, 133.3, 133.0, 132.2, 129.8, 127.9, 126.9, 124.5, 124.0, 122.1, 121.9, 121.2, 119.5, 117.4, 111.7, 95.8, 46.9, 21.6 ppm; HRMS (ESI) calcd for (C_31_H_24_N_2_O_2_S) [M + H]^+^; 489.1635; found, 489.0335.

#### 3.3.20 (3*E*)-*N*-Tosyl-3-[(4-methylphenyl)­methylidene]-2,3-dihydro-1*H*-indol-2-imine (**7d**)

Yellow solid; yield: 64%; *R*_*f*_ = 0.87 (Hexane/EtOAc = 9:1); mp 127–128 °C; IR (neat): ν_max_ = 3366, 3056, 2933, 1501, 1217, 830, 737 cm^–1^. ^1^H NMR (400 MHz, CDCl_3_): δ = 10.2 (s, 1H), 8.14 (s, 1H), 7.97 (d, *J* = 8.4 Hz, 2H), 7.77 (d, *J* = 7.6 Hz, 1H), 7.58 (d, *J* = 8 Hz, 2H), 7.35–7.27 (m, 5H), 7.10–7.10 (m, 1H), 7.00–6.96 (m, 1H), 2.49–2.46 (m, 6H); ^13^C NMR (100 MHz, CDCl_3_): δ = 174.2, 154.6, 144.9, 135.4, 135.3, 135.0, 133.3, 133.0, 132.2, 129.8, 127.9, 126.9, 124.0, 122.1, 121.9, 121.4, 121.2, 119.5, 117.4, 111.7, 95.8, 46.9, 21.6 ppm; HRMS (ESI) calcd for (C_23_H_20_N_2_O_2_S) [M + H]^+^; 389.1323; found, 389.1230.

#### 3.3.21 (3*E*)-*N*-Tosyl-3-[(4-bromophenyl)­methylidene]-5-fluoro-2,3-dihydro-1-methylindol-2-imine (**7e**)

Red solid; yield: 73%; *R*_*f*_ = 0.75 (Hexane/EtOAc = 9:1); mp 142–143 °C; IR (neat): ν_max_ = 3156, 2924, 1566, 1472, 1335, 1157, 1084, 813, 746, 678 cm^–1^. ^1^H NMR (400 MHz, CDCl_3_): δ = 8.76 (s, 1H), 7.98 (d, *J* = 8 Hz, 2H), 7.67 (d, *J* = 8 Hz, 2H), 7.51–7.50 (m, 1H), 7.40–7.34 (m, 1H), 7.17 (dd, *J*_1_ = 8 Hz, *J*_2_ = 8 Hz 1H), 7.10 (dd, *J*_1_ = 8 Hz, *J*_2_ = 8 Hz 1H), 6.93–6.90 (m, 1H), 3.53 (s, 3H), 2.48 (s, 3H); ^13^C NMR (100 MHz, CDCl_3_): δ = 160.4, 159.2, 158.0, 144.8, 142.3, 141.1, 140.2, 133.2, 132.2, 130.7, 129.3, 127.8, 127.0, 126.2, 124.8, 123.2, 123.1, 116.5, 116.3, 110.7, 110.4, 110.1, 110.0, 30.1, 21.5 ppm; HRMS (ESI) calcd for (C_23_H_18_BrFN_2_O_2_S) [M + H]^+^; 485.0334; found, 485.0355.

#### 3.3.22 (3*E*)-*N*-Tosyl-3-[(3,5-dinitrophenyl)­methylidene]-5-fluoro-2,3-dihydro-1-methylindol-2-imine (**7f**)

Red solid; yield: 67%; *R*_*f*_ = 0.73 (Hexane/EtOAc = 9:1); mp 150–151 °C; IR (neat): ν_max_ = 3384, 3054, 2924, 1548, 1482, 1317, 1009, 790 cm^–1^. ^1^H NMR (400 MHz, CDCl_3_): δ = 8.22 (s, 1H), 8.27–7.92 (m, 1H), 7.78–7.76 (m, 2H), 7.39 (d, *J* = 8 Hz, 4H), 7.15–7.10 (m, 2H), 6.36 (d, *J*_1_ = 2.4 Hz, 1H), 3.40 (s, 3H), 2.44 (s, 3H); ^13^C NMR (100 MHz, CDCl_3_): δ = 187.7, 163.2, 161.3, 153.6, 143.1, 139.7, 139.1, 126.9, 125.6, 118.0, 117.2, 116.9, 111.7, 111.5, 111.0, 111.0, 109.9, 83.6, 29.5, 21.5 ppm; HRMS (ESI) calcd for (C_23_H_17_FN_4_O_6_S) [M + H]^+^; 497.0931; found, 497.0941.

#### 3.3.23 (3*E*)-*N*-Tosyl-3-[(4-methylphenyl)­methylidene]-2,3-dihydro-1,4-bromobenzylindol-2-amine (**7g**)

Red solid; yield: 70%; *R*_*f*_ = 0.72 (Hexane/EtOAc = 9:1); mp 138–139 °C; IR (neat): ν_max_ = 3067, 2923, 2857, 1586, 1488, 1337, 1149, 1004, 818, 754 cm^–1^. ^1^H NMR (400 MHz, CDCl_3_): δ = 9.10 (s, 1H), 7.87 (d, *J*_1_ = 8.4 Hz, 2H), 7.65–7.61 (m, 3H), 7.42 (d, *J*_1_ = 8.4 Hz, 3H), 7.35–7.21 (m, 3H), 7.14 (d, *J*_1_ = 8.4 Hz, 2H), 6.95–6.92 (m, 1H), 6.88–6.86 (m, 2H), 5.01 (s, 2H), 2.49–2.46 (m, 6H); ^13^C NMR (100 MHz, CDCl_3_): δ = 174.2, 154.6, 144.9, 135.4, 135.3, 135.0, 133.3, 133.0, 132.2, 129.8, 127.9, 126.9, 124.0, 122.1, 121.9, 121.4, 121.2, 119.5, 117.4, 111.7, 95.8, 46.9, 21.6 ppm; HRMS (ESI) calcd for (C_30_H_25_BrN_2_O_2_S) [M + H]^+^; 557.0898; found, 557.0901.

### 3.4 General Procedure for Products **8**

To an oven-dried RB flask, 1 mmol of the appropriate **6** and a drop of DBU in dichloroethane were stirred. To this reaction mixture was added 1 mmol of the appropriate dibromo compound, and the reaction was stirred at 50 °C for 3 to 4 h. The completion of the reaction was monitored using TLC. After the completion of the reaction, the solvent was removed under reduced pressure and diluted with ethyl acetate (10 mL) and water (15 mL). The organic phase was separated and the aqueous layer washed with ethyl acetate (10 mL). The concentration of combined organic layers under reduced pressure afforded the crude product, which was purified by column chromatography using silica gel (100–200 mesh, EtOAc/hexane 10:90) to afford product **8**.

#### 3.4.1 16-(Thiophen-3-yl)-5,11-dimethyl-6,10-ditosyl-5,6,7,8,9,10,11,16-octahydro-[1,5]­diazecino­[6,7-*b*:10,9-*b*′]­diindole (**8a**)

White solid; yield: 52%; *R*_*f*_ = 0.76 (Hexane/EtOAc = 3:1); mp 135–136 °C; IR (neat): ν_max_ = 2926, 1594, 1504, 1427, 1347, 1264, 1160, 732 cm^–1^. ^1^H NMR (400 MHz, CDCl_3_): δ = 7.80 (d, *J* = 8 Hz, 4H), 7.22 (d, *J* = 8 Hz, 7H), 6.93–6.91 (m, 3H), 6.82–6.80 (m, 3H), 6.74–6.74 (m, 3H), 3.86–3.81 (m, 10H), 2.36 (s, 6H), 1.65–1.60 (m, 4H), 1.32–1.24 (m, 2H); ^13^C NMR (100 MHz, CDCl_3_): δ = 155.0, 142.4, 140.4, 133.7, 129.4, 127.4, 126.2, 125.9, 122.9, 119.2, 117.0, 110.0, 64.2, 42.7, 29.0, 27.7, 26.7, 21.5 ppm; HRMS (ESI) calcd for (C_40_H_39_N_4_O_4_S_3_) [M + H]^+^; 735.2133; found, 735.2139.

#### 3.4.2 17-(3,4,5-Trimethoxyphenyl)-3,14-dibromo-5,12-dimethyl-6,11-ditosyl-6,7,8,9,10,11,12,17-octahydro-5*H*-[1,6]­diazacycloundecino­[7,8-*b*:11,10-*b*′]­diindole (**8b**)

White solid; yield: 70%; *R*_*f*_ = 0.69 (Hexane/EtOAc = 9:1); mp 140–142 °C; IR (neat): ν_max_ = 2925, 2856, 1593, 1463, 1344, 1158, 1120, 810, 737, 671 cm^–1^. ^1^H NMR (400 MHz, CDCl_3_): δ = 7.90 (d, *J* = 8 Hz, 4H), 7.32 (s = 2H), 7.24–7.22 (m, 2H), 7.10–7.10 (m, 4H), 7.00–6.97 (m, 2H), 6.43 (s, 2H), 5.84 (s, 1H), 4.45–4.40, (m, 2H), 3.93 (s, 3H), 3.61–3.60 (m, 8H), 3.13 (s, 6H), 2.34 (s, 6H), 1.84 (m, 2H), 1.42–1.41 (m, 2H); ^13^C NMR (100 MHz, CDCl_3_): δ 152.6, 143.9, 138.3, 136.8, 136.3, 133.7, 131.5, 129.7, 127.2, 126.9, 125.5, 125.1, 116.0, 112.9, 110.9, 106.9, 61.3, 56.1, 51.4, 37.4, 29.4, 26.4, 21.4 ppm; HRMS (ESI) calcd for (C_46_H_46_^81^Br_2_N_4_O_7_S_2_) [M + H]^+^; 991.1232; found, 991.1237.

#### 3.4.3 18-(*p*-Tolyl)-5,13-dimethyl-6,12-ditosyl-5,6,7,8,9,10,11,12,13,18-decahydro-[1,7]­diazacyclododecino­[2,3-*b*:6,5-*b*′]­diindole (**8c**)

White solid; yield: 64%; *R*_*f*_ = 0.74 (Hexane/EtOAc = 9:1); mp 139–140 °C; IR (neat): ν_max_ = 3059, 2927, 1587, 1282, 1082, 815, 769, 669 cm^–1^. ^1^H NMR (400 MHz, CDCl_3_): δ = 7.96 (d, *J* = 8 Hz, 4H), 7.67 (s, 2H), 7.66–7.45 (m, 2H), 7.37–7.28 (m, 7H), 6.71 (s, 1H), 6.62 (d, *J* = 8.8 Hz, 3H), 6.45 (d, *J* = 8.4 Hz, 2H), 4.48–4.45 (m, 2H), 3.75 (s, 6H), 2.93–2.87 (m, 2H), 2.72 (s, 3H), 2.48 (6H), 2.10–2.02 (m, 2H); ^13^C NMR (100 MHz, CDCl_3_): δ = 157.9, 144.0, 140.2, 136.1, 134.5, 132.7, 130.3, 129.7, 129.6, 129.0, 128.5, 127.3, 125.6, 125.0, 122.7, 113.3, 112.6, 111.7, 110.1, 55.3, 44.7, 35.6, 20.3, 21.5 ppm; HRMS (ESI) calcd for (C_45_H_46_N_4_O_4_S_2_) [M + H]^+^; 771.3039; found, 771.3048.

#### 3.4.4 19-(4-Bromophenyl)-5,14-diethyl-6,13-ditosyl-6,7,8,9,10,11,12,13,14,19-decahydro-5*H*-[1,7]­diazacyclotridecino­[2,3-*b*:6,5-*b*′]­diindole (**8d**)

White solid; yield: 51%; *R*_*f*_ = 0.68 (Hexane/EtOAc = 3:1); mp 140–141 °C; IR (neat): ν_max_ = 2929, 1616, 1455, 1340, 1158, 1090, 818, 737 cm^–1^. ^1^H NMR (400 MHz, CDCl_3_): δ = 8.10 (d, *J* = 8 Hz, 4H), 7.91 (d, *J* = 8 Hz, 2H), 7.47–7.33 (m, 5H), 7.20 (d, *J* = 8.4 Hz, 4H), 6.74 (d, *J* = 8.4 Hz, 4H), 6.67 (d, *J* = 7.6 Hz, 2H), 4.10 (dd, *J*_1_ = 14.4 Hz, *J*_2_ = 4.4 Hz 4H), 3.81 (q, *J* = 6.8 Hz, 4H), 3.35 (t, *J* = 14.8 Hz, 4H), 2.75 (dd, *J*_1_ = 16 Hz, *J*_2_ = 4 Hz, 4H) 2.55 (s, 6H), 0.66 (m, 10H), 2.36 (s, 6H), 0.66 (t, *J* = 7.2 Hz, 6H); ^13^C NMR (100 MHz, CDCl_3_): δ = 165.0, 143.9, 142.7, 141.3, 136.0, 131.1, 130.0, 129.6, 128.2, 126.2, 125.3, 123.6, 121.7, 110.9, 61.1, 48.9, 43.8, 39.2, 21.6, 11.4 ppm; HRMS (ESI) calcd for (C_47_H_49_^81^BrN_4_O_4_S_2_) [M + H]^+^; 877.2457; found, 877.2465.

#### 3.4.5 21-(4-Methoxyphenyl)-5,16-diethyl-6,15-ditosyl-6,7,8,9,10,11,12,13,14,15,16,21-dodecahydro-5*H*-[1,7]­diazacyclopentadecino­[2,3-*b*:6,5-*b*′]­diindole (**8e**)

White solid; yield: 46%; *R*_*f*_ = 0.70 (Hexane/EtOAc = 3:1); mp 152–153 °C; IR (neat): ν_max_ = 2926, 2855, 1594, 1502, 1347, 1159, 1122, 812, 738, 672 cm^–1^. ^1^H NMR (400 MHz, CDCl_3_): δ = 7.24 (d, *J* = 7.6 Hz, 2H), 6.83 (d, *J* = 7.2 Hz, 1H), 6.42–6.14 (m, 7H), 6.23 (m, 4H), 5.87–5.76 (m, 3H), 5.54 (d, *J* = 7.2 Hz, 1H), 4.11–3.93 (m, 5H), 3.68–3.62 (m, 3H), 3.41–2.96 (m, 5H), 2.94–2.91 (m, 3H) 2.67 (s, 6H), 1.54–1.45 (m, 10H), 0.54 (t, *J* = 6.8 Hz, 6H); ^13^C NMR (100 MHz, CDCl_3_): δ = 172.9, 161.8, 156.8, 142.2, 141.9, 141.2, 136.2, 135.2, 129.9, 129.4, 129.0, 128.8, 127.5, 126.8, 126.1, 48.9, 43.8, 39.2, 21.6, 11.4 ppm; HRMS (ESI) calcd for (C_50_H_56_N_4_O_5_S_2_) [M + H]^+^; 857.3770; found, 857.3773.

#### 3.4.6 2-(4-Bromophenyl)-1^1^,3^1^-diethyl-4,8-ditosyl-1^1^ *H*,3^1^ *H*-4,8-diaza-1,3­(3,2)-diindola-6­(1,4)-benzenacyclooctaphane (**8f**)

White solid; yield: 39%; *R*_*f*_ = 0.74 (Hexane/EtOAc = 3:1); mp 148–150 °C; IR (neat): ν_max_ = 3058, 2925, 1724, 1157, 1121, 809, 734, 672 cm^–1^. ^1^H NMR (400 MHz, CDCl_3_): δ = 7.87 (d, *J* = 8 Hz, 5H), 7.33–7.26 (m, 12H), 7.15–7.09 (m, 4H), 6.89–6.86 (m, 4H), 6.95 (d, *J* = 8 Hz, 3H), 4.18 (s, 4H), 3.92 (q, *J* = 7.2 Hz, 4H), 2.40 (s, 6H), 1.26 (t, *J* = 7.2 Hz, 6H); ^13^C NMR (100 MHz, CDCl_3_): δ = 156.5, 149.5, 144.3, 136.6, 135.1, 129.6, 127.5, 126.3, 124.6, 120.4, 116.9, 113.8, 112.1, 55.8, 39.2, 21.5, 14.7 ppm; HRMS (ESI) calcd for (C_49_H_45_BrN_4_O_4_S_2_) [M + H]^+^; 897.2144; found, 897.2149.

#### 3.4.7 1^1^,3^1^-Dibenzyl-2-phenyl-4,8-ditosyl-1^1^ *H*,3^1^ *H*-4,8-diaza-1,3­(3,2)-diindola-6­(1,4)-benzenacyclooctaphane (**8g**)

White solid; yield: 54%; *R*_*f*_ = 0.72 (Hexane/EtOAcD = 9:1); mp 153–155 °C; IR (neat): ν_max_ = 2929, 1617, 1456, 1342, 1158, 1090, 735, 664 cm^–1^. ^1^H NMR (400 MHz, CDCl_3_): δ = 7.75 (d, *J* = 8 Hz, 4H), 7.64 (d, *J* = 8 Hz, 2H), 7.23–7.10 (m, 22H), 7.07–6.94 (m, 6H), 6.54–6.53 (m, 2H), 6.26 (s, 4H), 5.09 (s, 4H), 2.35 (s, 6H); ^13^C NMR (100 MHz, CDCl_3_): δ = 155.2, 142.5, 140.0, 137.4, 136.2, 134.6, 133.5, 129.4, 128.7, 128.2, 128.0, 127.1, 126.3, 126.0, 123.2, 121.8, 121.1, 119.7, 119.0, 117.0, 110.4, 109.7, 101.9, 64.4, 49.7, 45.7, 21.5 ppm; HRMS (ESI) calcd for (C_59_H_50_N_4_O_4_S_2_) [M + H]^+^; 943.3352; found, 943.3358.

#### 3.4.8 1^1^,3^1^-Dimethyl-2-(thiophen-3-yl)-4,8-ditosyl-1^1^ *H*,3^1^ *H*-4,8-diaza-1,3­(3,2)-diindola-6­(1,3)-benzenacyclooctaphane (**8h**)

White solid; yield: 63%; *R*_*f*_ = 0.69 (Hexane/EtOAc = 9:1); mp 142–143 °C; IR (neat): ν_max_ = 3074, 2928, 1547, 1502, 1314, 1221, 1056, 1091, 830 cm^–1^. ^1^H NMR (400 MHz, CDCl_3_): δ = 7.84 (d, *J* = 8 Hz, 4H), 7.30–7.27 (m, 10H), 7.23–7.22 (m, 4H), 6.95 d, *J* = 7.2 Hz, 4H), 6.82–6.77 (m, 4H), 5.17 (s, 4H), 3.84 (s, 6H), 2.44 (s, 6H); ^13^C NMR (100 MHz, CDCl_3_): δ = 154.0, 143.4, 135.5, 135.4, 133.4, 131.6, 128.7, 127.1, 122.7, 119.7, 112.3, 109.9, 106.7, 105.5, 106.9, 55.8, 49.1, 30.2, 21.4; ^13^C NMR (100 MHz, CDCl_3_): δ ppm; HRMS (ESI) calcd for (C_45_H_40_N_4_O_4_S_3_) [M + H]^+^; 797.2289; found, 797.2291.

## Supplementary Material







## Notes

Experimental procedures, analytical data, copy of spectra **8b**, ^1^H and ^13^C NMR spectroscopy, and HRMS details of all the compounds (PDF)
Crystal data for compound **6c** (CIF)
Cystal data for compound **8b** (CIF)
