# Supplementary material for: A Build, Couple, Pair (B/C/P) Strategy in the Synthesis of Diindole Fused Diazamacrocycles: An Attempt to a Green Synthetic Approach
Source: ACS Omega. 2025 Sep 23;10(39):46153–64. doi: 10.1021/acsomega.5c08238 (PMC12509125; doi:10.1021/acsomega.5c08238)
Supplement: Supplementary file 1 [file ao5c08238_si_001.pdf]

Supporting Information

for

A Build, Couple, Pair (B/C/P) Strategy in the Synthesis of Diindole Fused Diazamacrocycles: An Attempt to a Green Synthetic Approach

Rofin Mangali*[a,b]

[a]*School of Chemistry, Bharathidasan University, Tiruchirappalli-620 024, India*

[b]*St. Francis de Sales College (Autonomous), Electronics City, Bengaluru - 560 100, India*

*Tel: +91-8150857079; E-mail: francisrufin@gmail.com; rofinmangali@sfscollege.in

CONTENTS

1.	Crystal data for compound **6c** and **8b** (Figure S1 – S6)	S2-S4
2.	^{1}H and ^{13}C NMR of **6a - p** (Spectra S1 – S35)	S5-S39
3.	^{1}H and ^{13}C NMR of **7a** – **g** (Spectra S36 -S49)	S40-S53
4.	^{1}H and ^{13}C NMR of **8a** – **h** (Spectra S50 -S65)	S54-S69

1. Crystal data for product

Crystal Data for 6c: (CCDC 2284071) $C_{41}H_{39}BrN_4O_4S_2$, *M* = 796.8190, 0.98 x 0.40 x 0.30 mm, monoclinic, space group P -2_1/n (14) with *a* = 18.5607(3) Å, *b* = 16.4969(3) Å, *c* = 25.5991(5) Å, α = 90, β = 103.8080(10), γ = 90, *V* = 7611.77 Å^3, R_1 = 0.065, wR_2 = 0.1064 on observed data, z = 8, D_{calcd} = 1.425 mg cm^{-3}, *F(000)* = 616, Absorption coefficient = 0.365 mm^{-1}, 0.708 reflections. The X-ray single crystal data of the compound was collected on Bruker D8 quest Eco with Hypix-3000 detector, equipped with graphite monochromated radiation (λ = 0.668 Å) at *T* = 293.(2) K, The data interpretations were processed with Bruker D8 quest Eco diffraction.

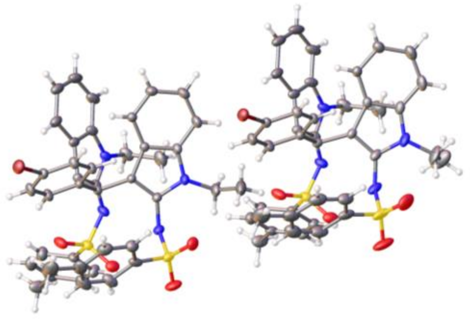

Figure S1. ORTEP view of compound **6c** showing two asymmetric units in unit cell.

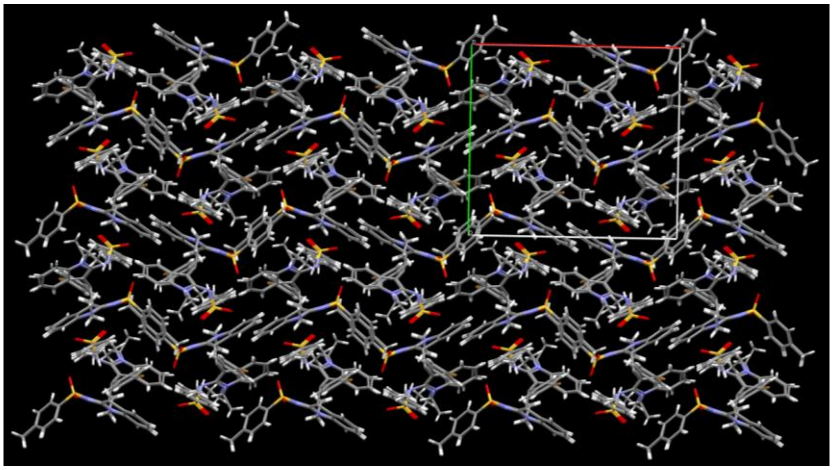

Figure S2. Partial crystal packing view of compound **6c** through *b*-axis

The molecules are arranged in three-dimensional network with the presence of a C–H···π and two hydrogen bonding interactions as described below.

C(38)-H(38A)---Cg(1); H(38A)---Cg(1) = 2.301 Å and < C(38)-H(36A)---Cg(1); H(38)--Cg(1) = 177.42°

C(40B)-H(40B)…S(2) = 2.331 Å and < C(40B)-H(40B)…S(2) = 117.81°

O(4)-H(19A)…C(19A) = 2.531 Å and < O(4)-H(19A)…C(19A) = 130.76º

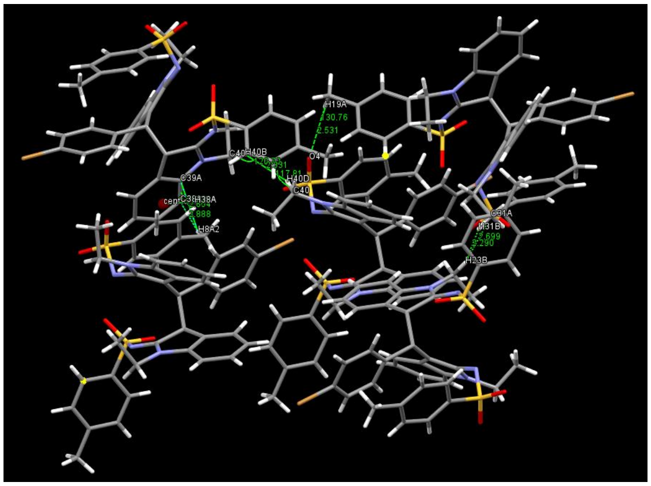

Figure S3. View of **6c** showing interactions, bond length and bond angle in the solid-state arrangement

Crystal Data for 8b: (CCDC 2293381) $C_{46}H_{46}Br_2N_4O_7S_2$, M = 991.1237, 0.652 x 0.666 x 0.639 mm, monoclinic, space group P -1(2) with a = 12.5902(3) Å, b = 12.6950(3) Å, c = 16.7072(5) Å, α = 69.512(2), β = 69.512(2), γ = 63.203(2), V = 2172.43 Å^3, R_1 = 0.0459, wR_2 = 0.0987 on observed data, z = 2, D_{calcd} = 1.515 g cm^{-3}, $F(000)$ = 1016.0, Absorption coefficient = 0.571 mm^{-1}, 0.0639 reflections. The X-ray single crystal data of the compound was collected on Xtlab synergy Rigaku oxford diffraction with Hypix-3000 detector, equipped with graphite monochromated radiation (λ = 0.7107 Å) at T = 298 K, The data interpretations were processed with CrysAlisPro, Xtlab Synergy Rigaku oxford diffraction.

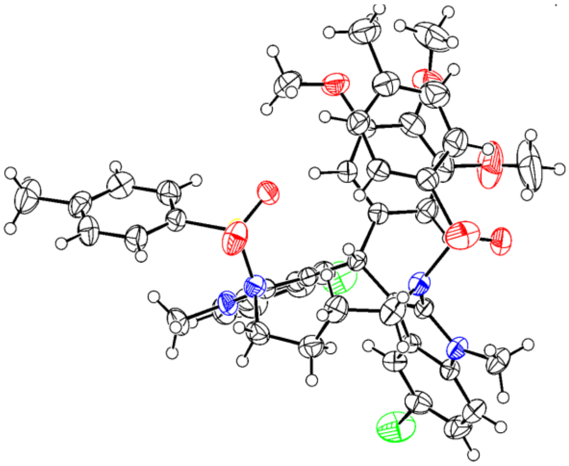

Figure S4. ORTEP view of compound **8b**

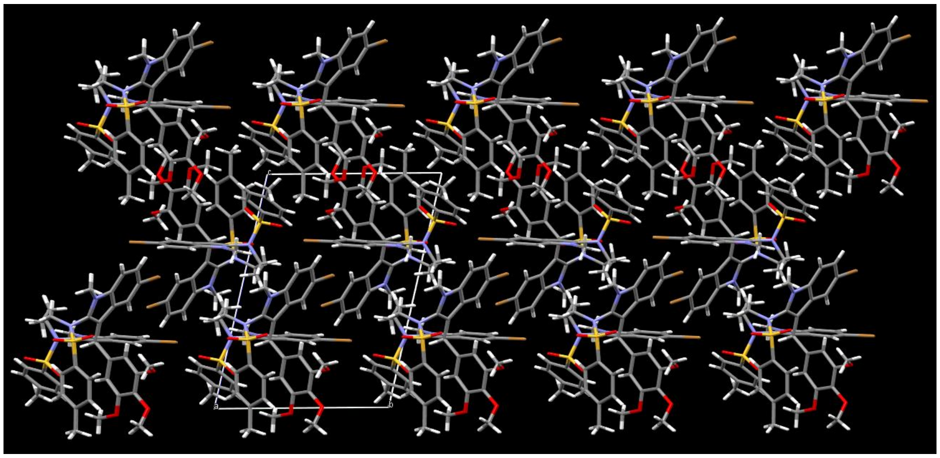

Figure S5. Partial crystal-packing view of compound **8b** through *b*-axis

The molecules are arranged in three-dimensional network with the presence of nine hydrogen bonding interactions as described below.

O(1)-H(43C)…C(43) = 2.548 Å and < O(1)-H(43C)…C(43) = 183.81^{o}

O(2)-H(34A)…C(34) = 2.792 Å and < O(2)-H(34A)…C(34) = 127.12^{o}

O(3)-H(42A)…C(42) = 2.520 Å and < O(3)-H(42A)…C(42) = 133.25^{o}

O(5)-H(45A)…C(45A) = 2.837 Å and < O(5)-H(45A)…C(45A) = 122.65^{o}

O(6)-H(30)…C(30) = 2.718 Å and < O(6)-H(30)…C(30) = 132.25^{o}

O(6)-H(45A)…C(45) = 2.637 Å and < O(6)-H(45A)…C(45) = 122.65^{o}

O(7)-H(33)…C(33) = 2.639 Å and < O(7)-H(33)…C(33) = 125.29^{o}

C(42)-H(42)…H(42B) = 2.191 Å and < C(42)-H(42)…H(42B) = 133.25^{o}

C(26)-H(45A)…H(45) = 2.338 Å and < C(26)-H(45A)…H(45) = 87.71^{o}

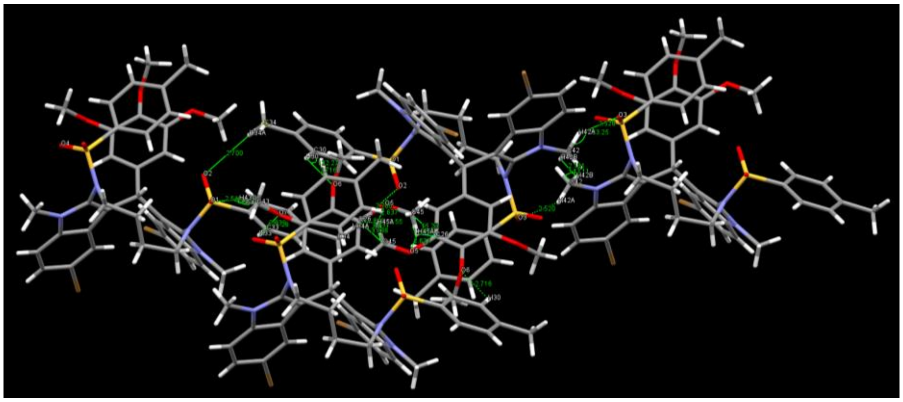

Figure S6. View of **8b** showing interactions, bond length and bond angle in solid-state arrangement

Scheme S2. Substrate scope for aldehydes[a] (6a-j)

3,3'-(Phenylmethane-1,1-diyl)bis(N-tosyl-1-ethylindol-2-amine)

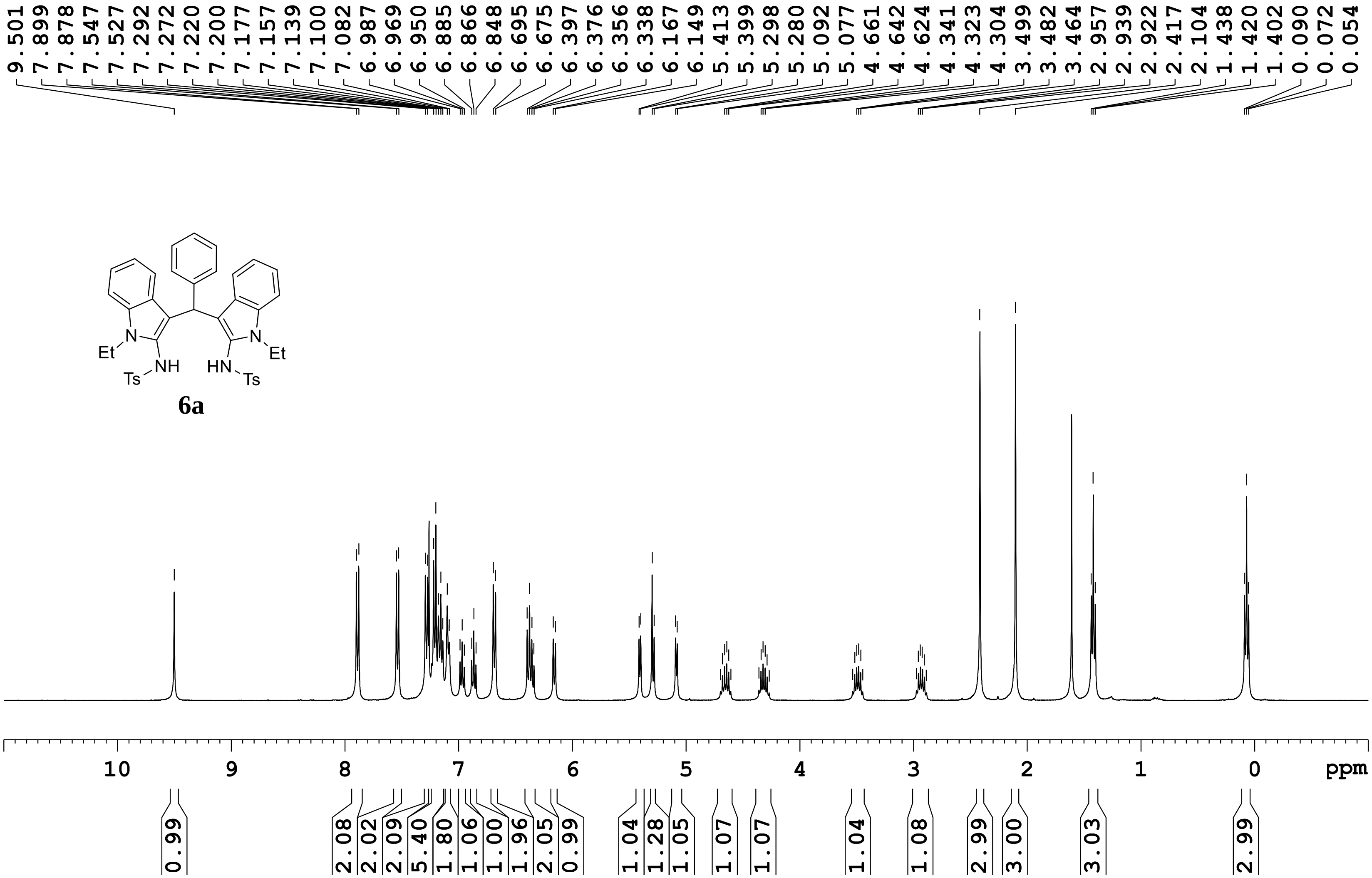

Spectra S1: ^{1}H NMR Spectrum for **6a**

3,3'-(Phenylmethane-1,1-diyl)bis(N-tosyl-1-ethylindol-2-amine)

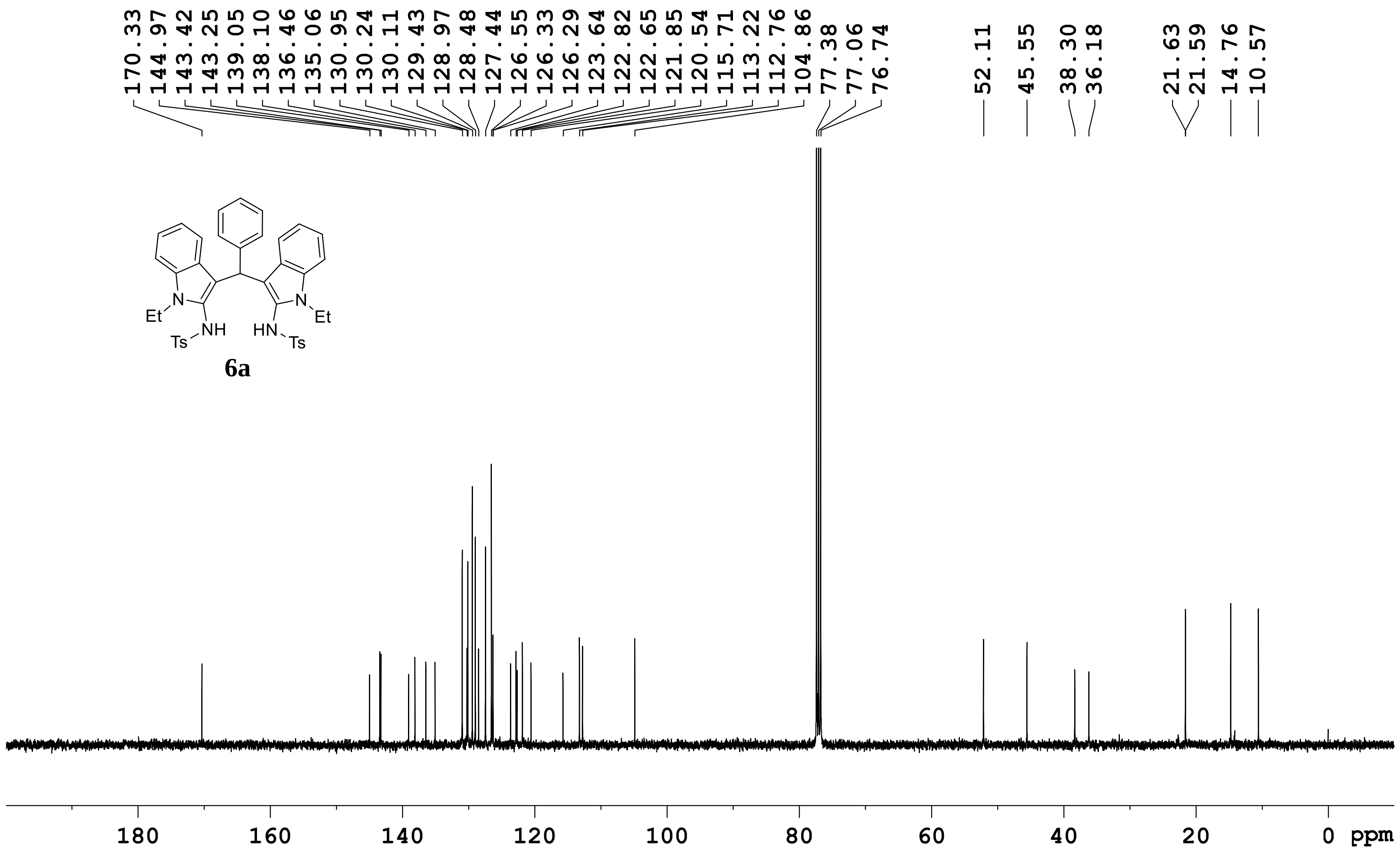

Spectra S2: ^{13}C NMR spectrum of **6a**

3,3'-(3-Chlorophenylmethane-1,1-diyl)bis(N-tosyl-1-methylindol-2-amine)

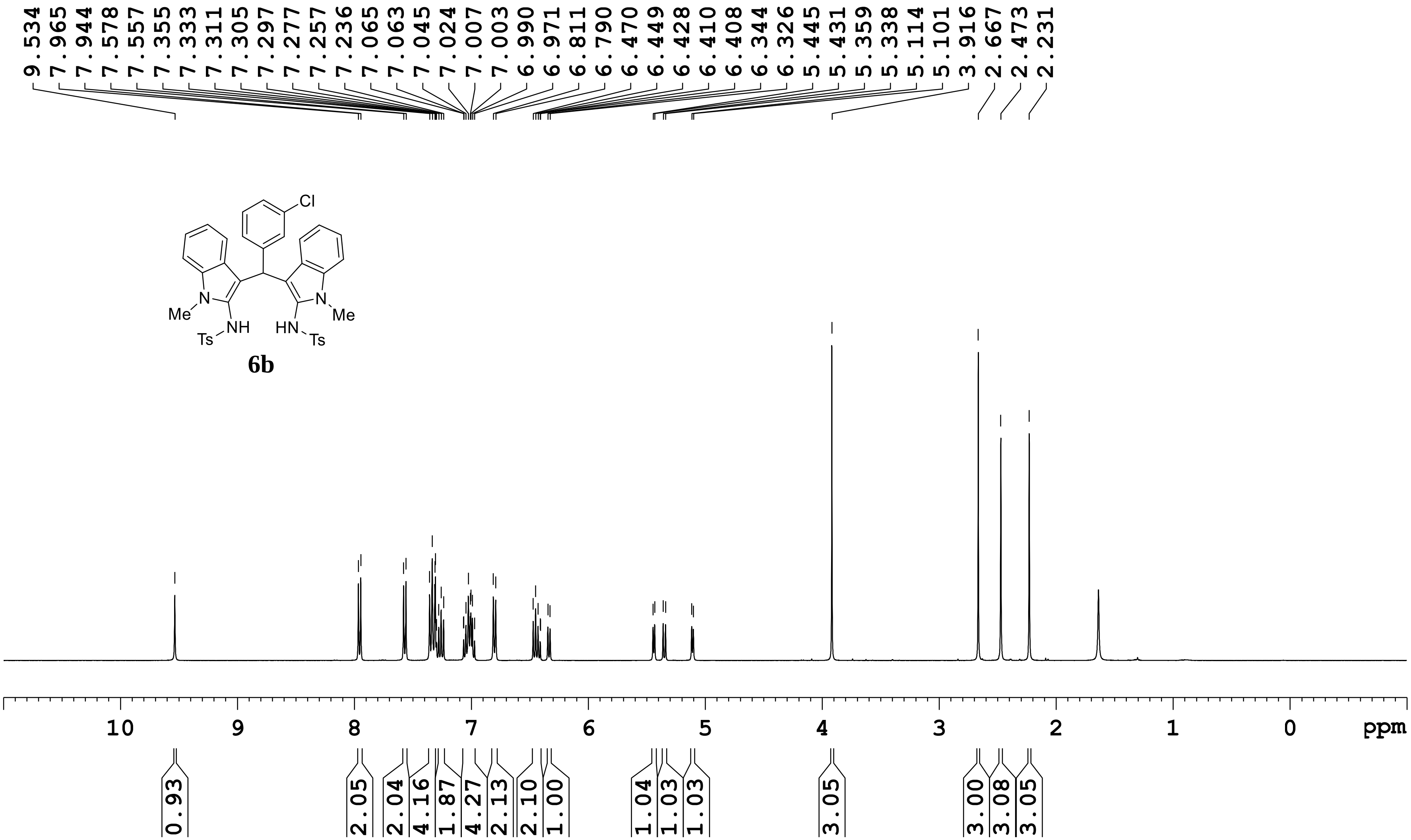

Spectra S3: ^{1}H NMR Spectrum for **6b**

3,3'-(3-Chlorophenylmethane-1,1-diyl)bis(N-tosyl-1-methylindol-2-amine)

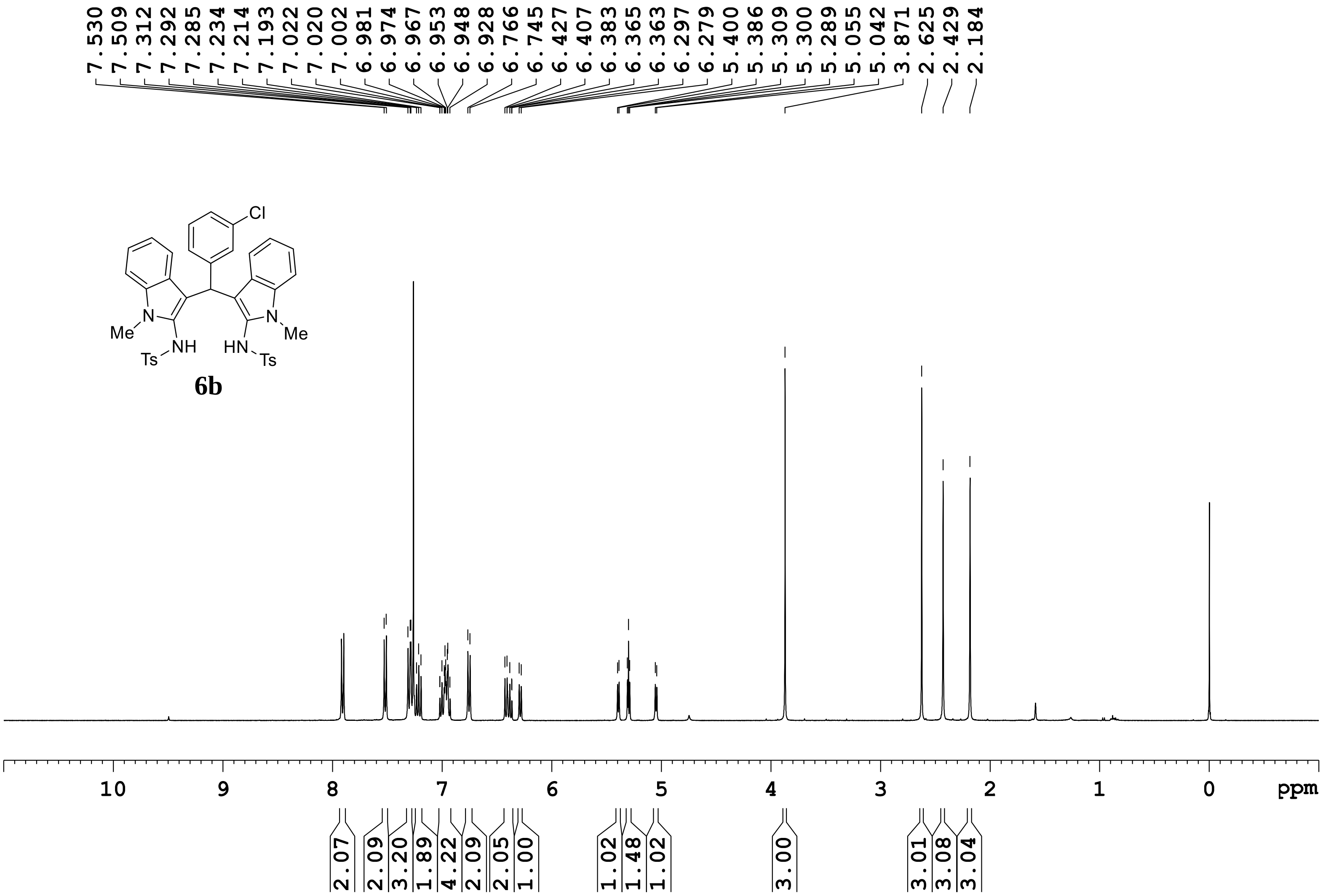

Spectra S4: ^{1}H NMR Spectrum after D_2O for **6b**

3,3'-(3-Chlorophenylmethane-1,1-diyl)bis(N-tosyl-1-methylindol-2-amine)

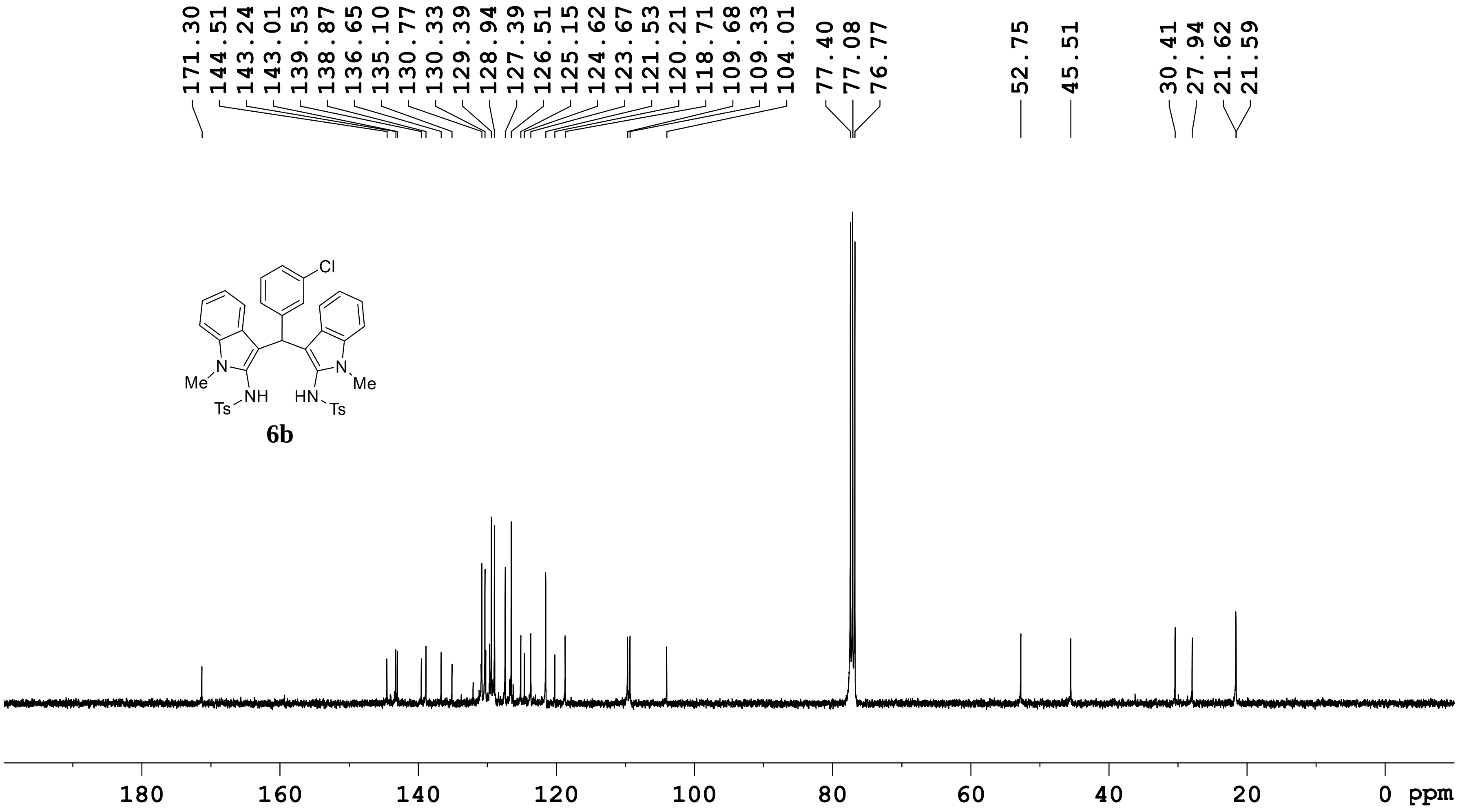

6b

Spectra S5: ^{13}C NMR Spectrum for **6b**

3,3'-(4-Bromophenylmethane-1,1-diyl)bis(N-tosyl-1-ethylindol-2-amine)

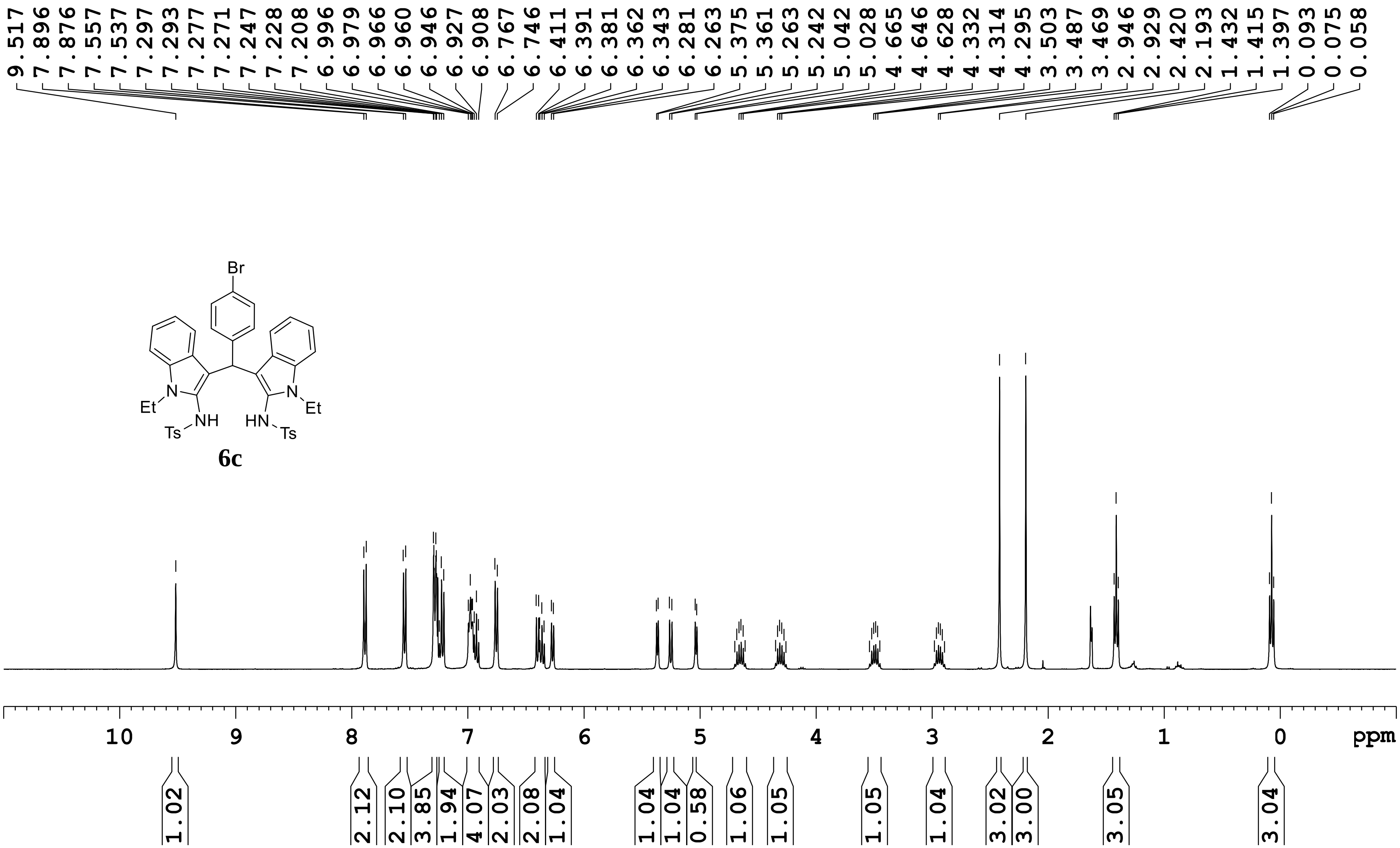

Spectra S6: ^{1}H NMR spectrum of **6c**

3,3'-(4-Bromophenylmethane-1,1-diyl)bis(N-tosyl-1-ethylindol-2-amine)

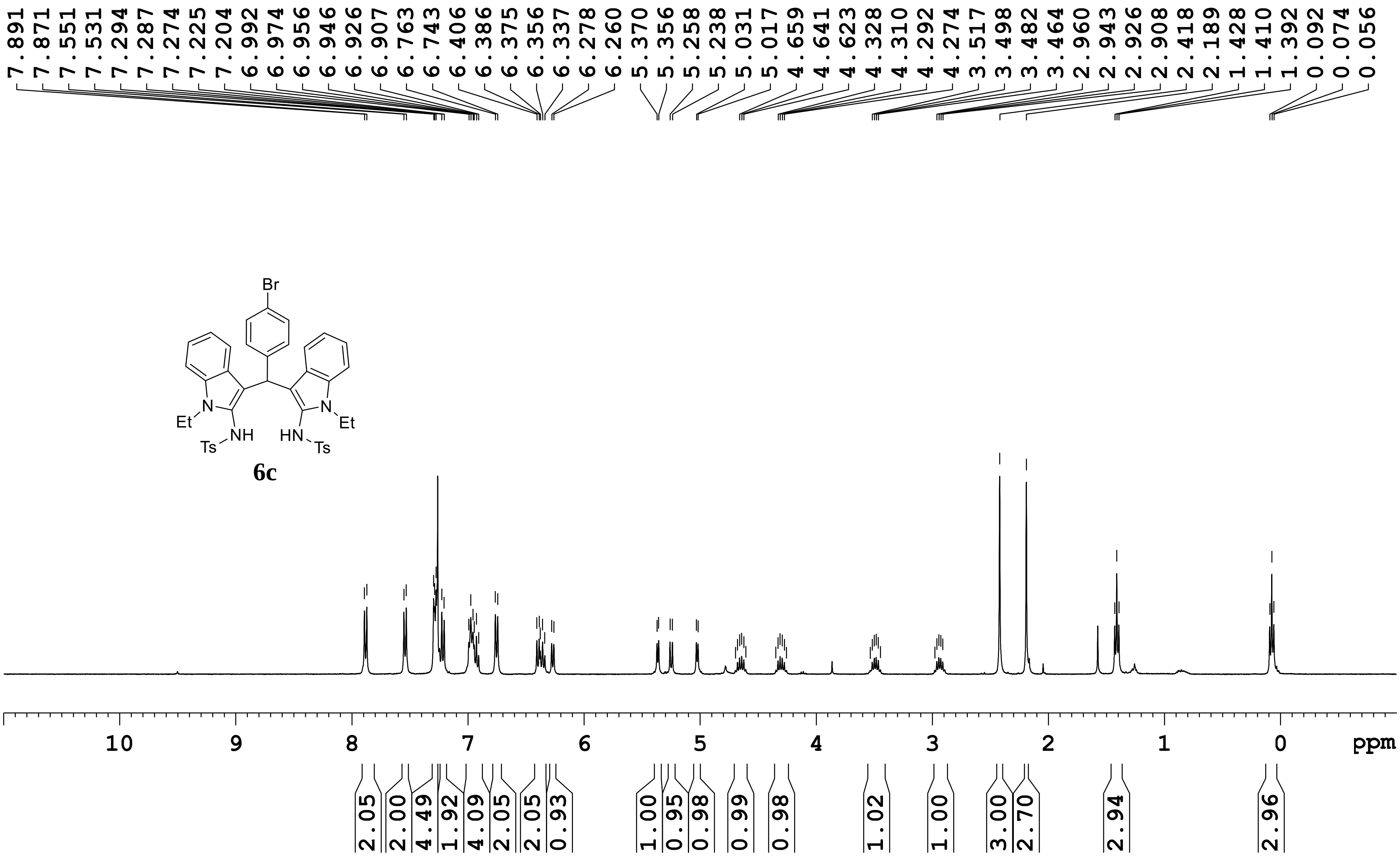

Spectra S7: ^{1}H NMR Spectrum after D_2O exchange for **6c**

3,3'-(4-Bromophenylmethane-1,1-diyl)bis(N-tosyl-1-ethylindol-2-amine)

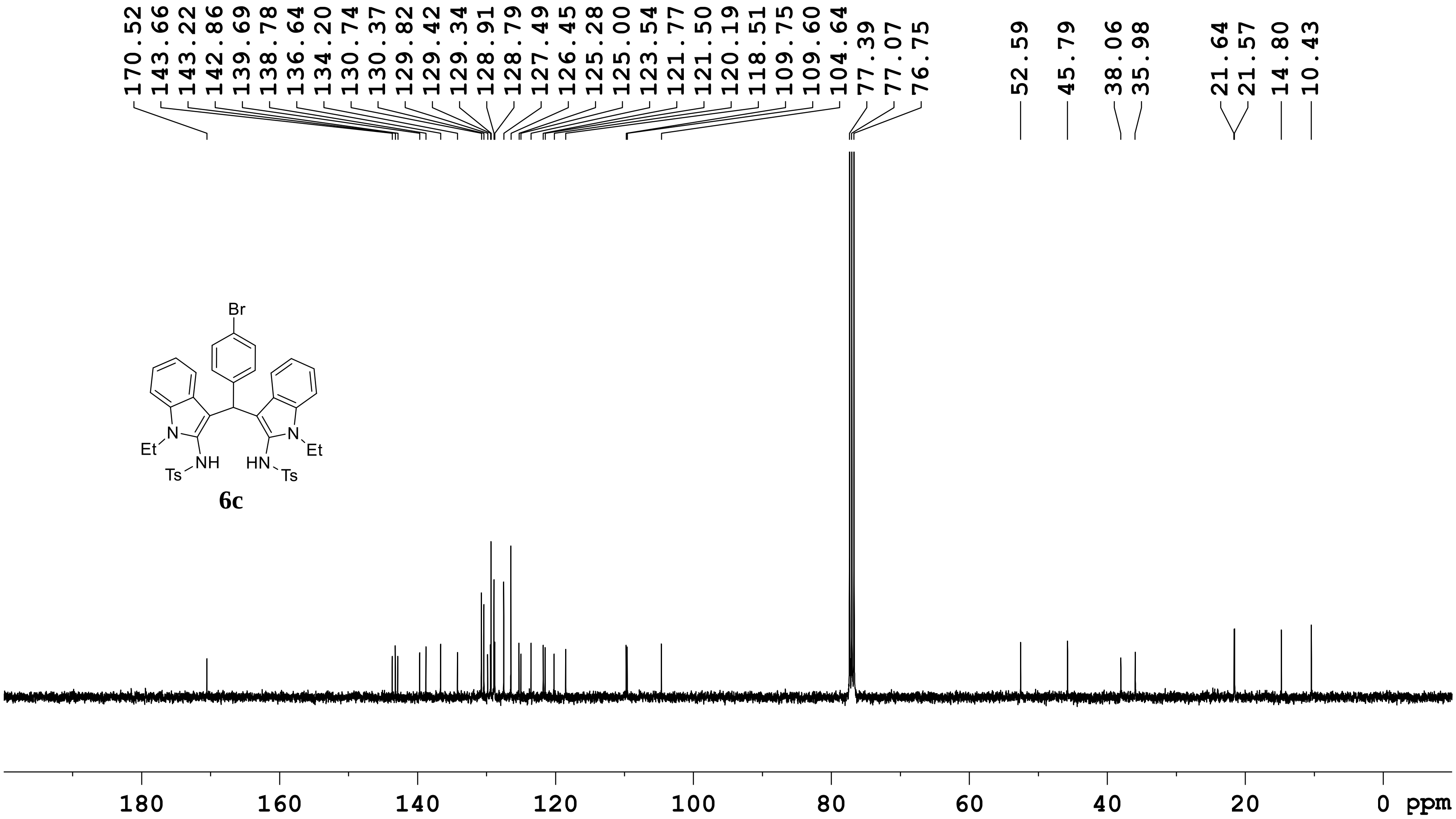

Spectra S8: ^{13}C NMR spectrum of **6c**

3,3'-(Tolylmethane-1,1-diyl)bis(N-tosyl-1-ethylindol-2-amine)

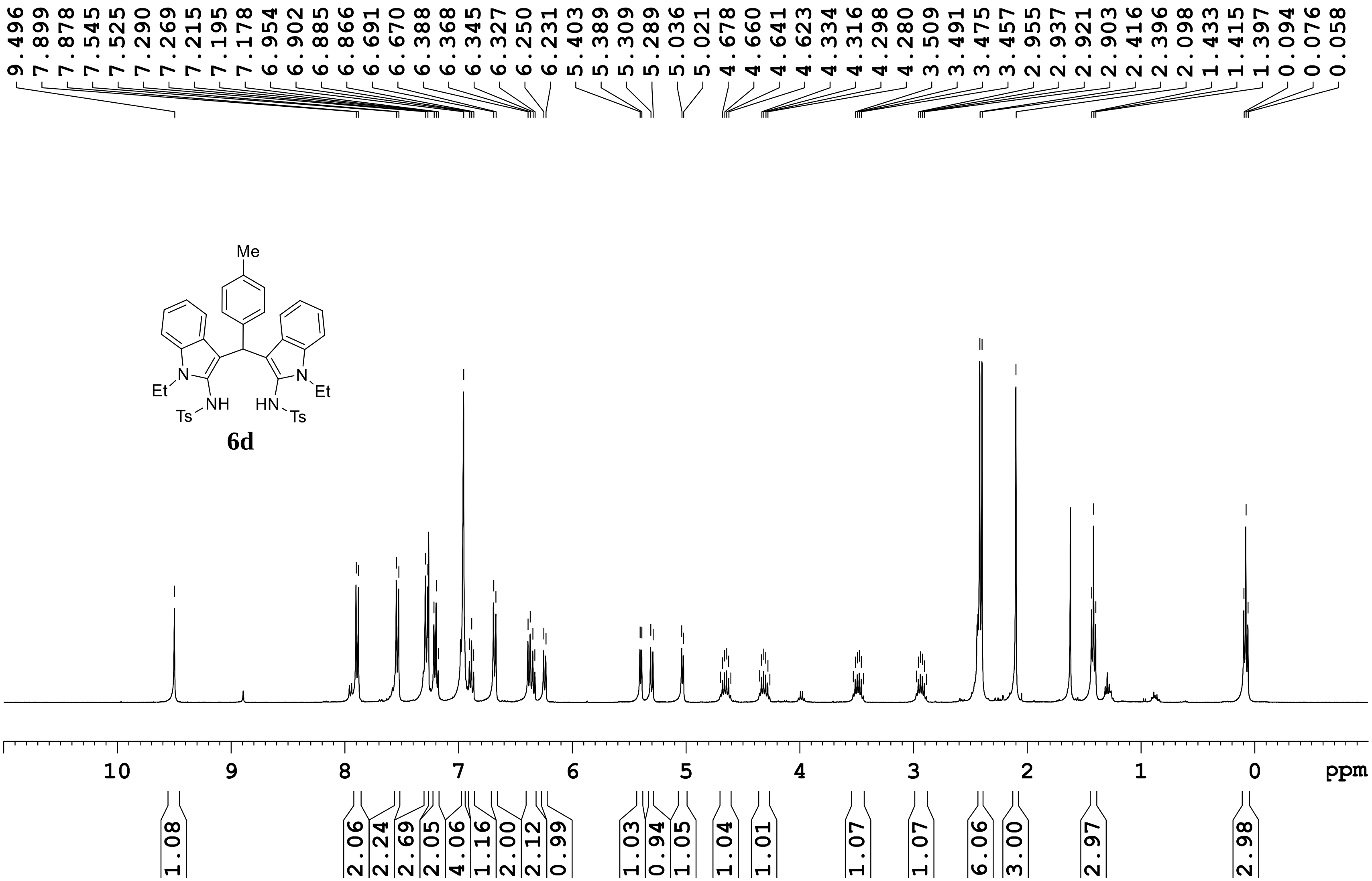

Spectra S9: ^{1}H NMR Spectrum for **6d**

3,3'-(Tolylmethane-1,1-diyl)bis(N-tosyl-1-ethylindol-2-amine)

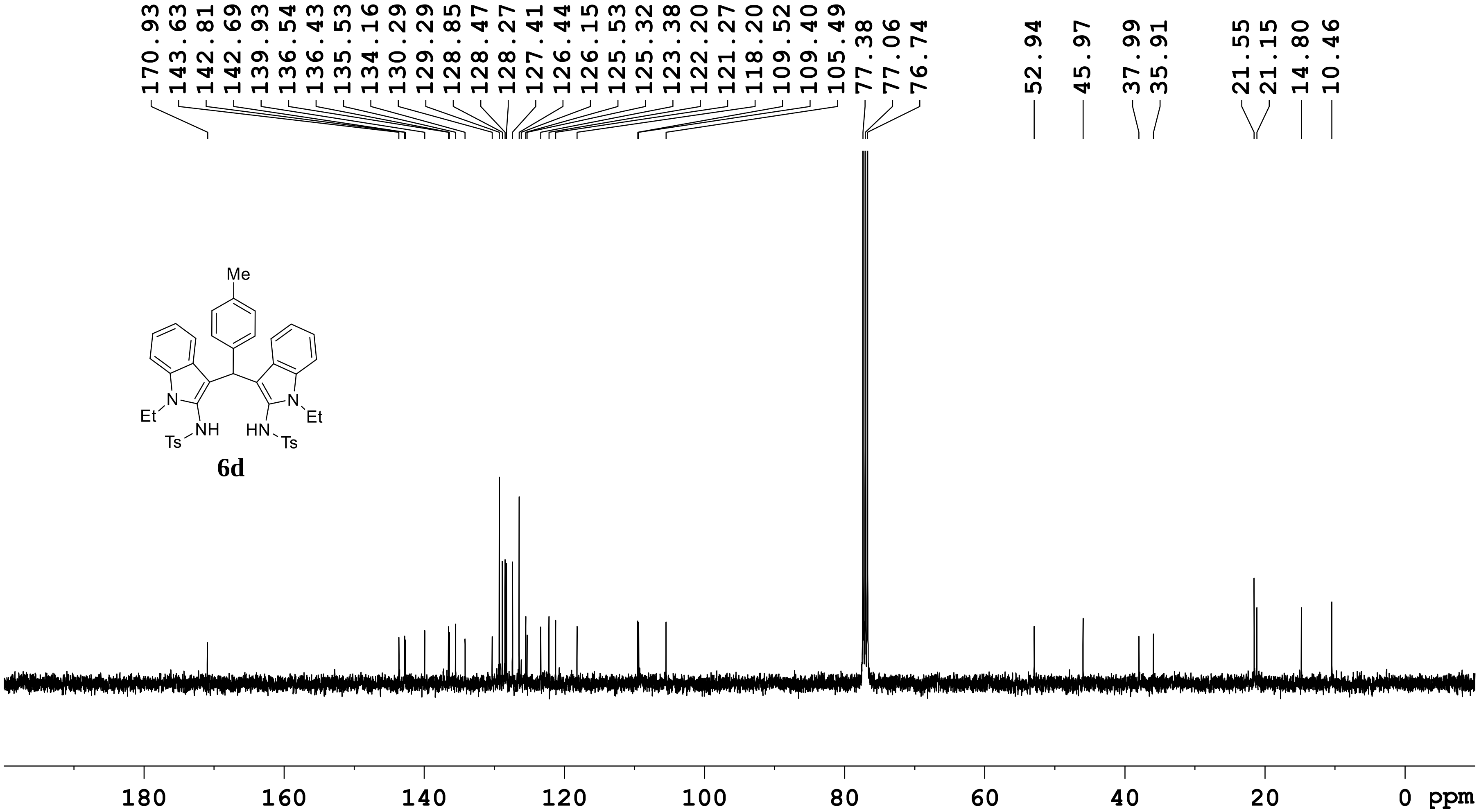

Spectra S10: ^{13}C NMR spectrum of **6d**

3,3'-(4-Methoxyphenylmethane-1,1-diyl)bis(N-tosyl-1-ethylindol-2-amine)

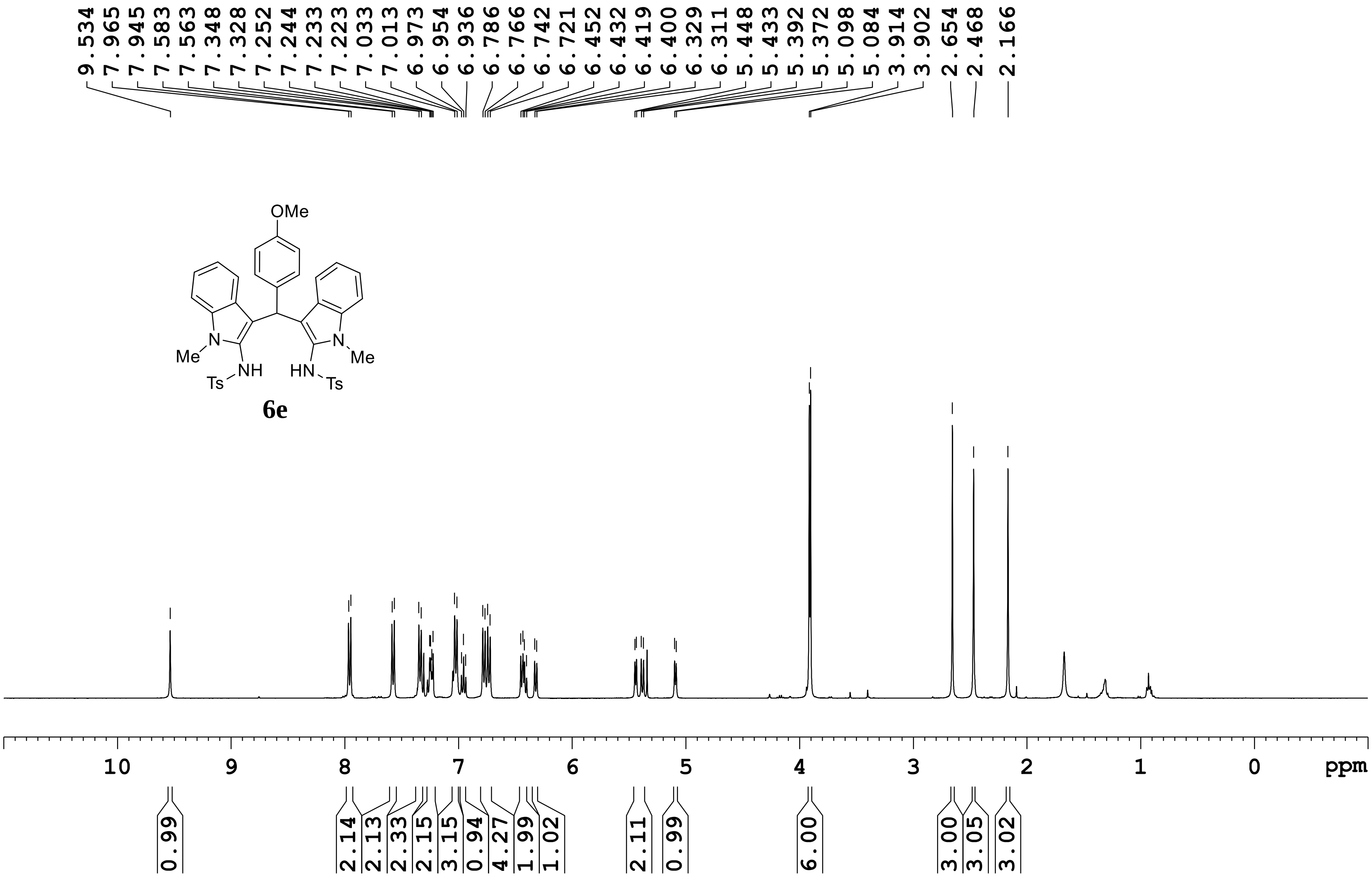

Spectra S11: ^{1}H NMR Spectrum for **6e**

3,3'-(4-Methoxyphenylmethane-1,1-diyl)bis(N-tosyl-1-ethylindol-2-amine)

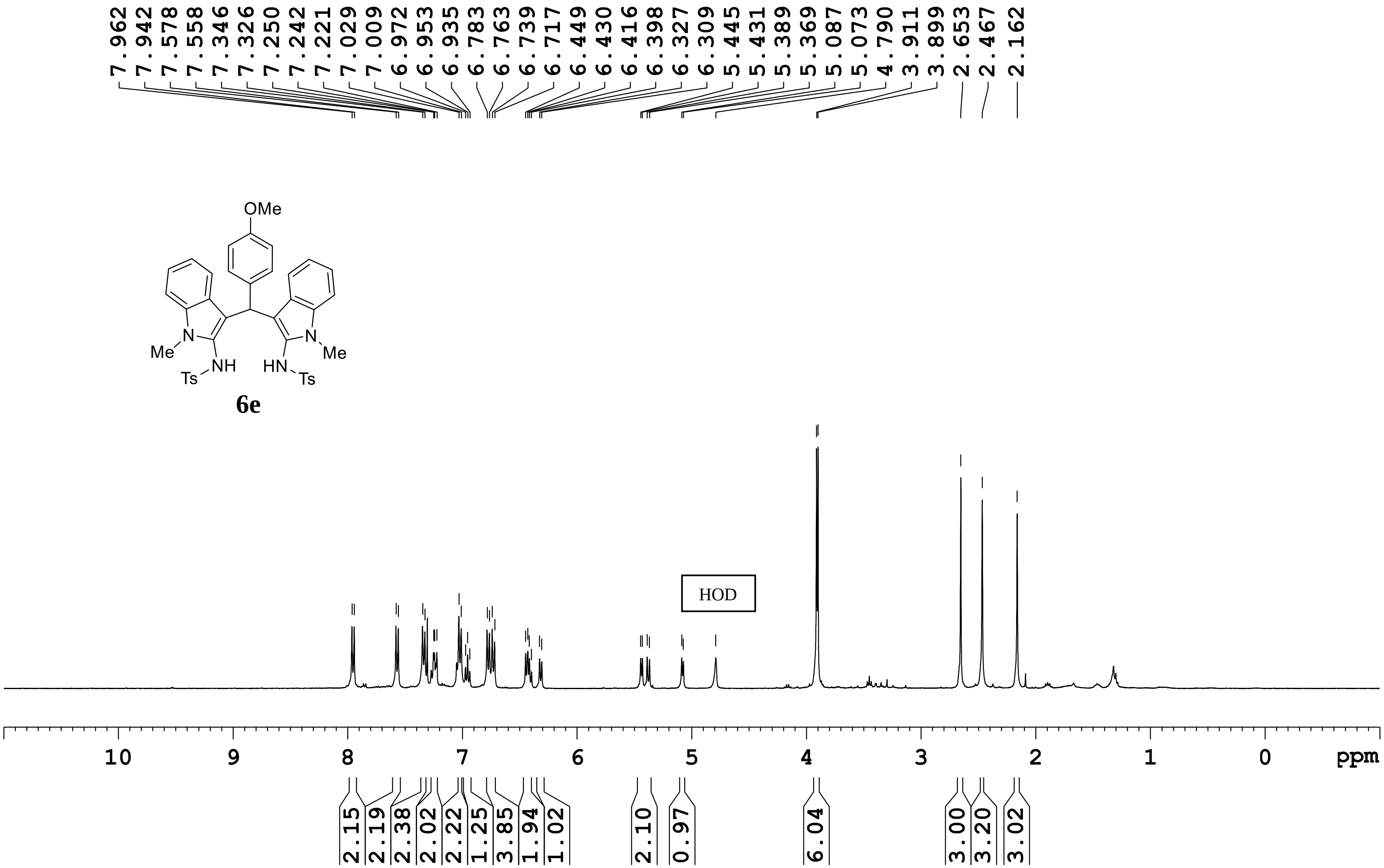

Spectra S12: ¹H NMR Spectrum after D_2O exchange for **6e**

3,3'-(4-Methoxyphenylmethane-1,1-diyl)bis(N-tosyl-1-ethylindol-2-amine)

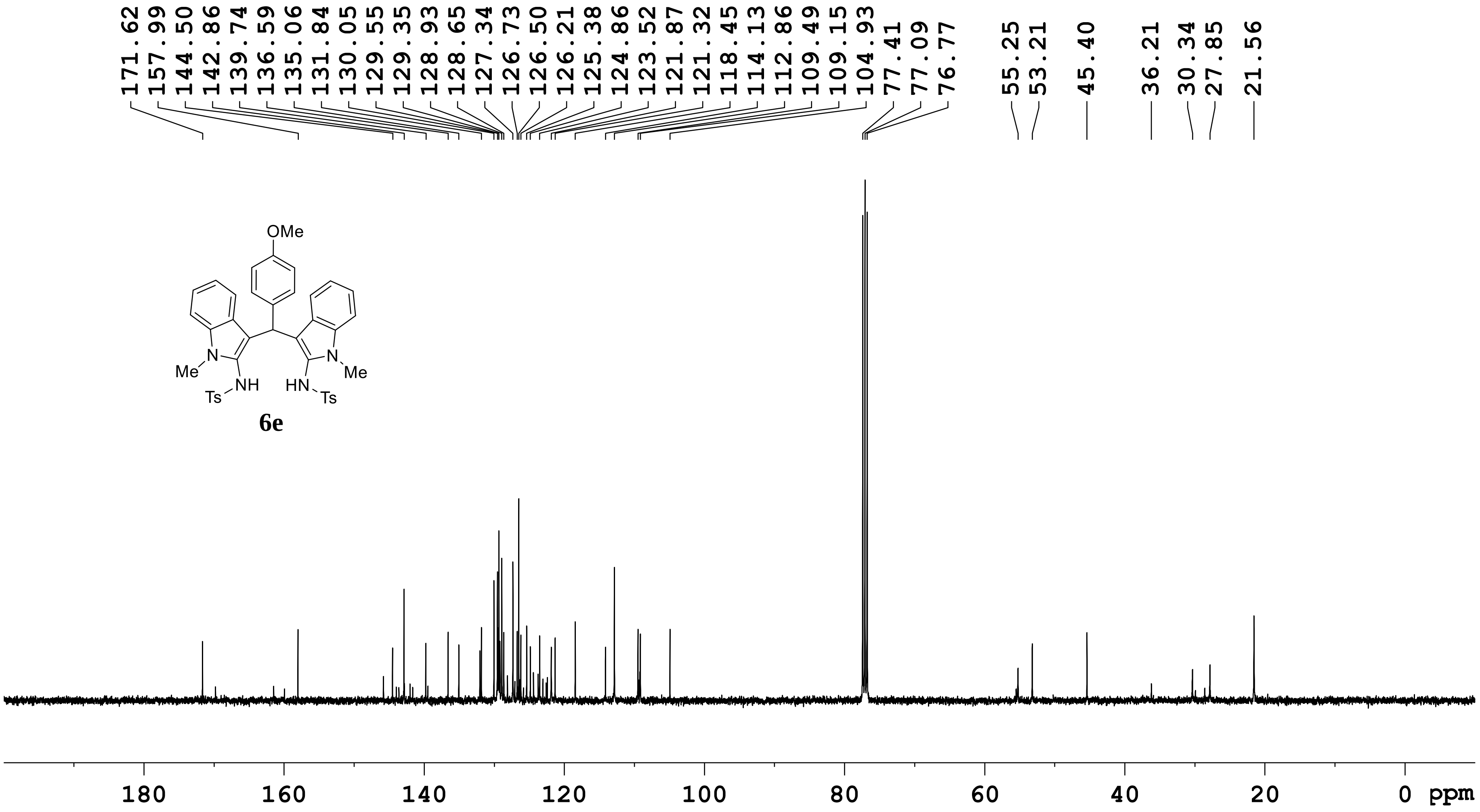

6e

Spectra S13: ^{13}C NMR Spectrum for **6e**

3,3'-(Thiophen-3-ylmethane-1,1-diyl)bis(N-tosyl-1-ethylindol-2-amine)

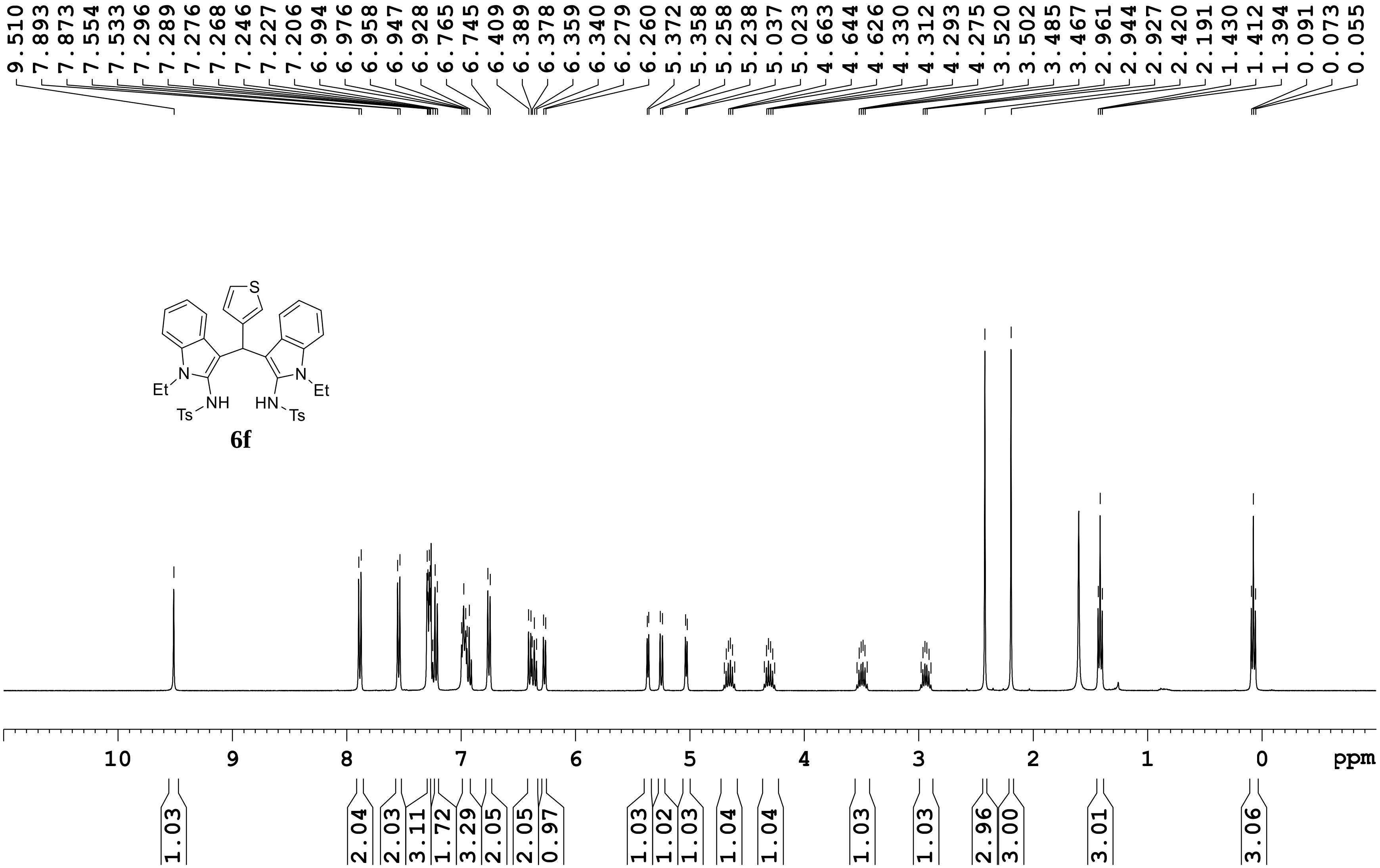

Spectra S14: ^{1}H NMR spectrum of **6f**

3,3'-(Thiophen-3-ylmethane-1,1-diyl)bis(N-tosyl-1-ethylindol-2-amine)

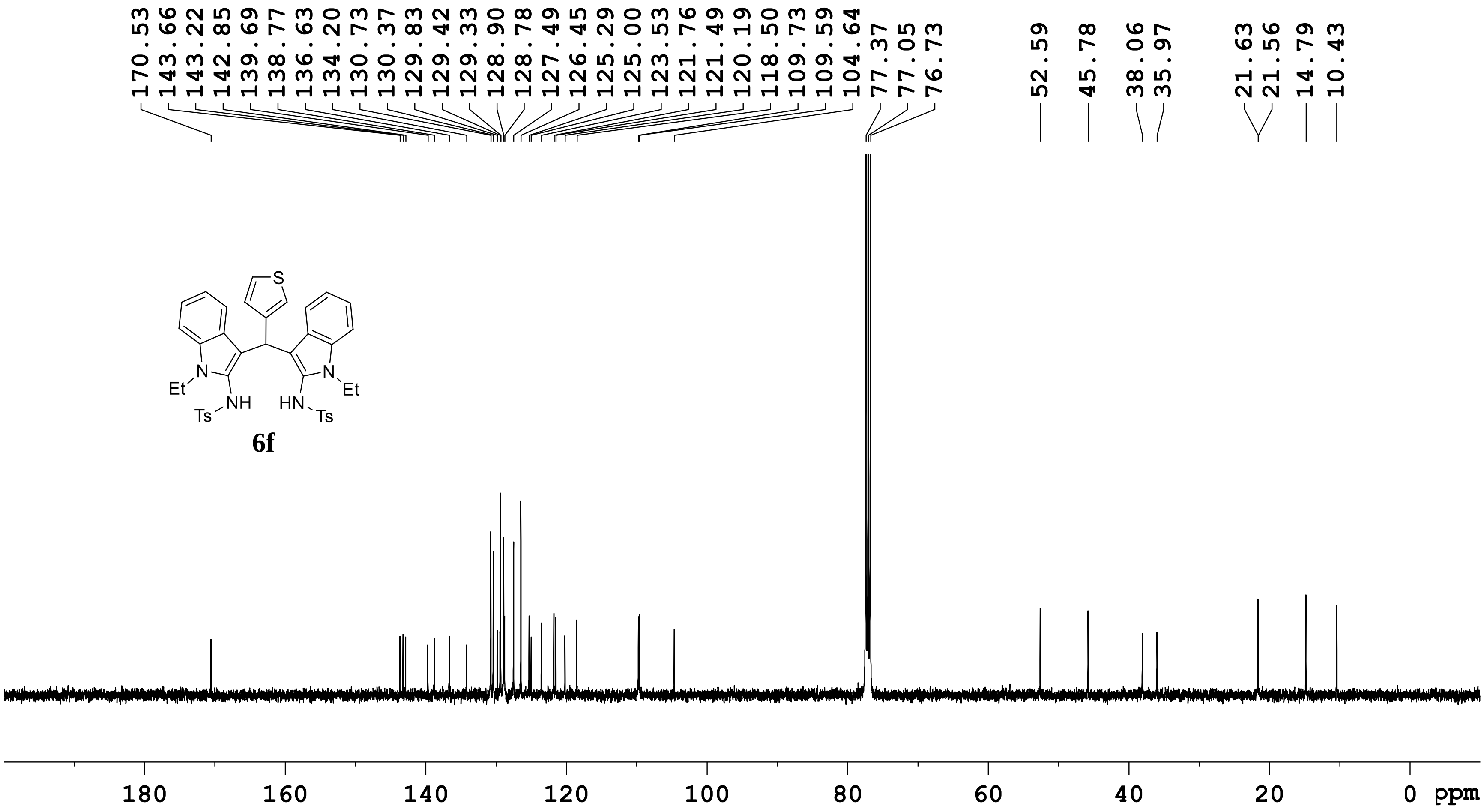

Spectra S15: ^{13}C NMR spectrum of **6f**

3,3'-(Furan-3-ylmethane-1,1-diyl)bis(N-tosyl-1-methylindol-2-amine)

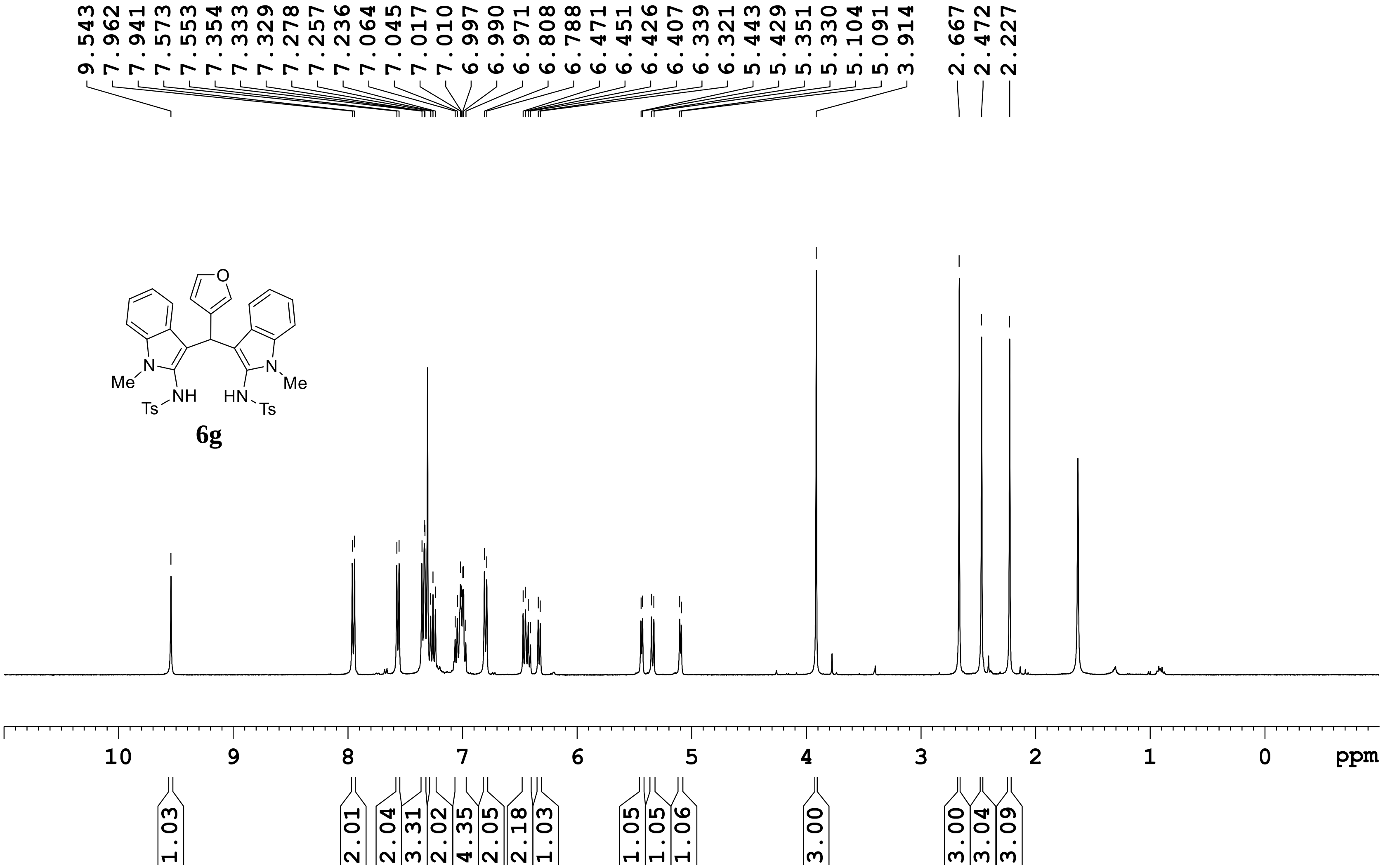

6g

Spectra S16: ^{1}H NMR spectrum of **6g**

3,3'-(Furan-3-ylmethane-1,1-diyl)bis(N-tosyl-1-methylindol-2-amine)

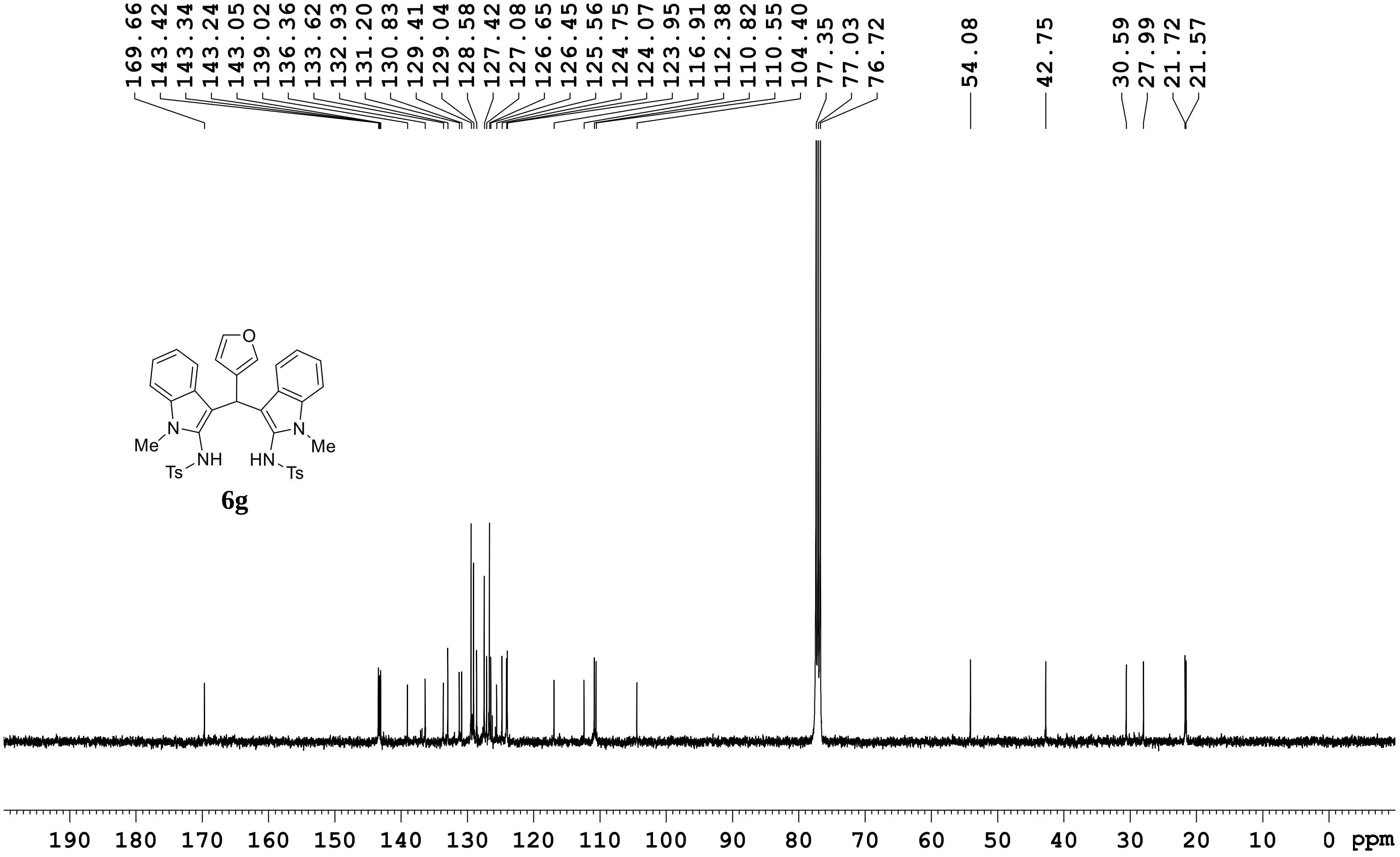

Spectra S17: ^{13}C NMR spectrum of **6g**

3,3'-(3,4,5-Trimethoxyphenylmethane-1,1-diyl)bis(N-tosyl-1-methyl-indol2-amine)

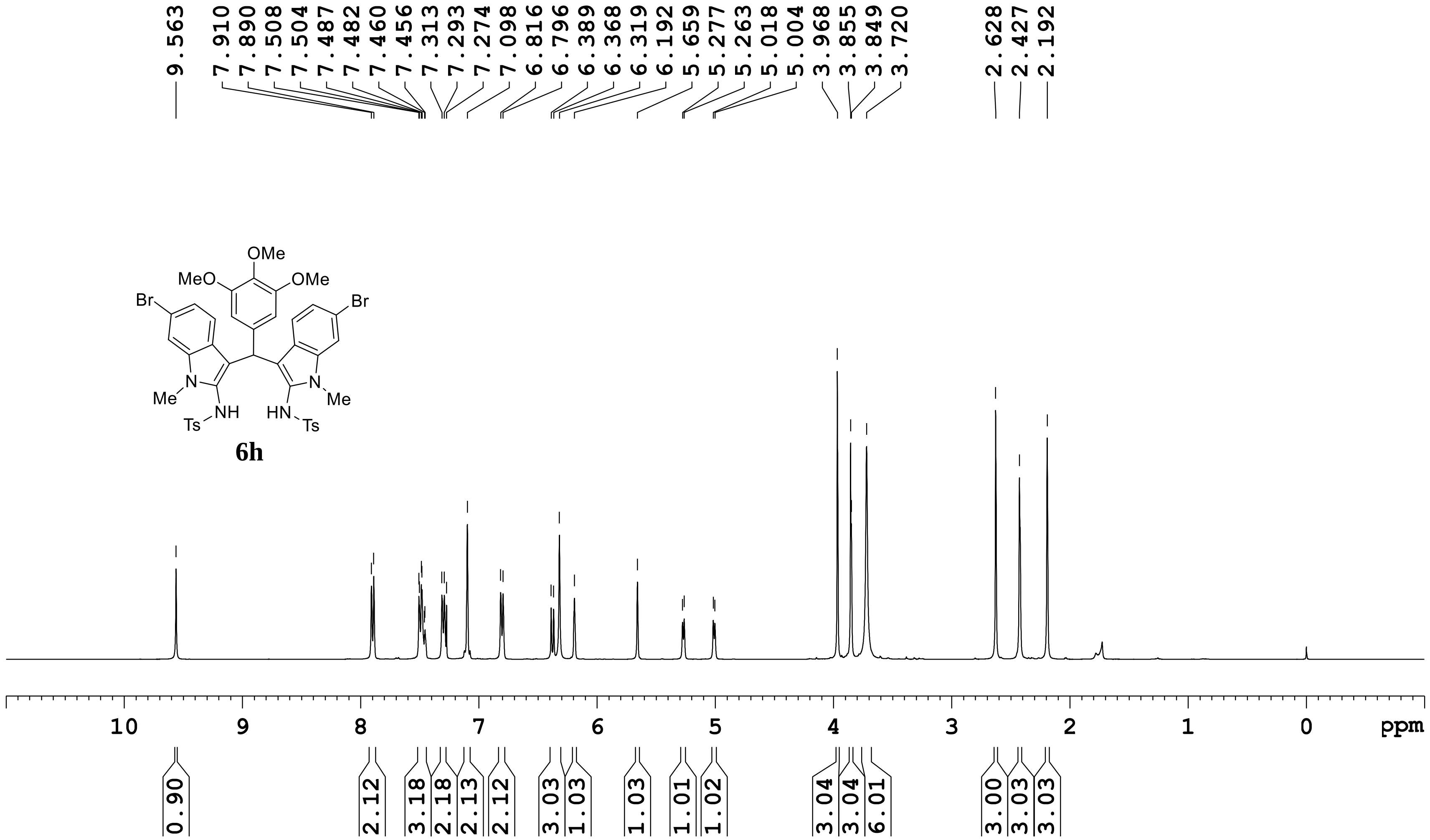

Spectra S18: ^{1}H NMR spectrum of **6h**

3,3'-(3,4,5-Trimethoxyphenylmethane-1,1-diyl)bis(N-tosyl-1-methyl-indol2-amine)

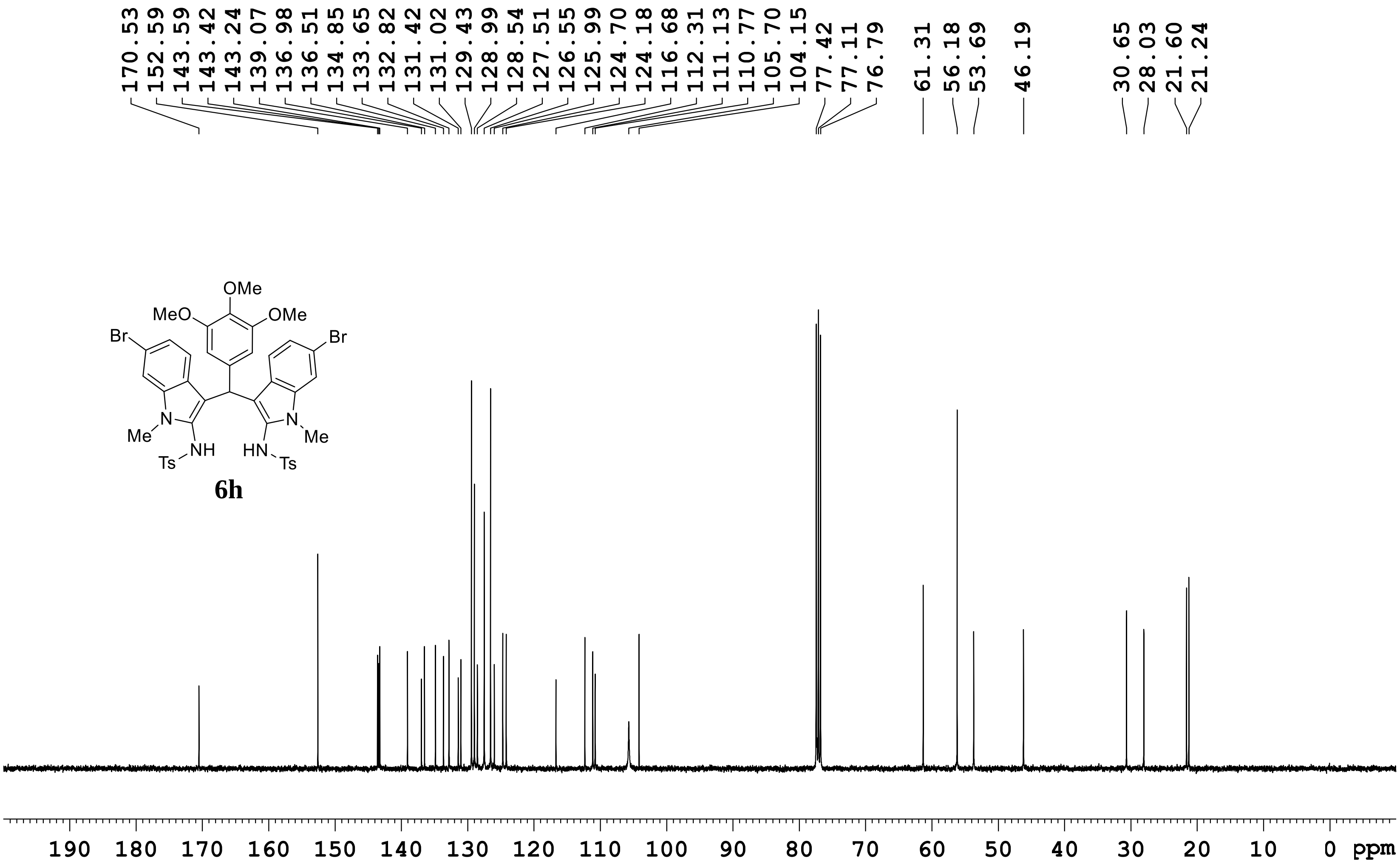

Spectra S19: ^{13}C NMR spectrum of **6h**

3,3'-(3,5-Dinitrophenylmethane-1,1-diyl)bis(N-tosyl-1-methylindol-2-amine)

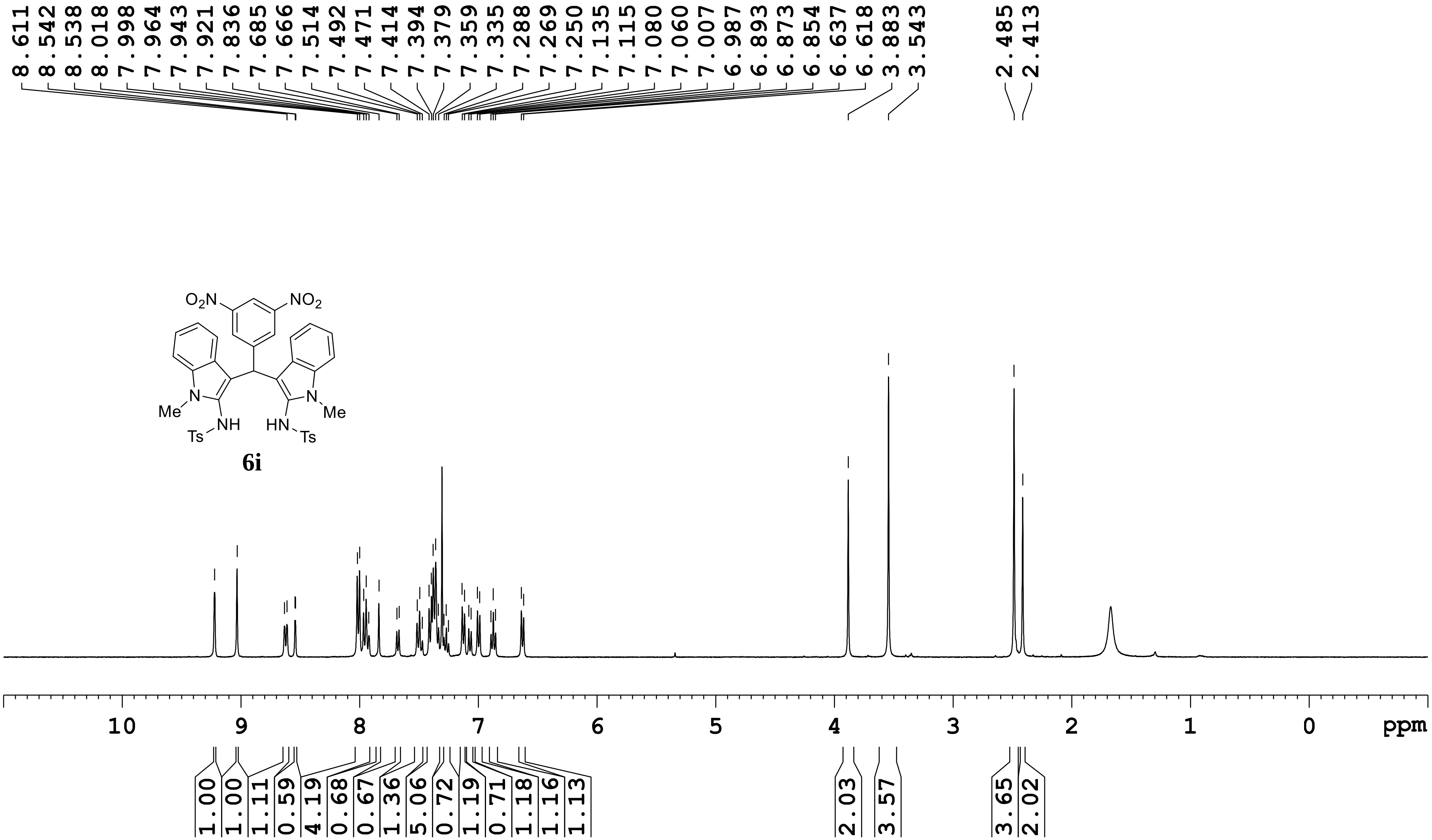

Spectra S20: ^{1}H NMR spectrum of **6i**

3,3'-(3,5-Dinitrophenylmethane-1,1-diyl)bis(N-tosyl-1-methylindol-2-amine)

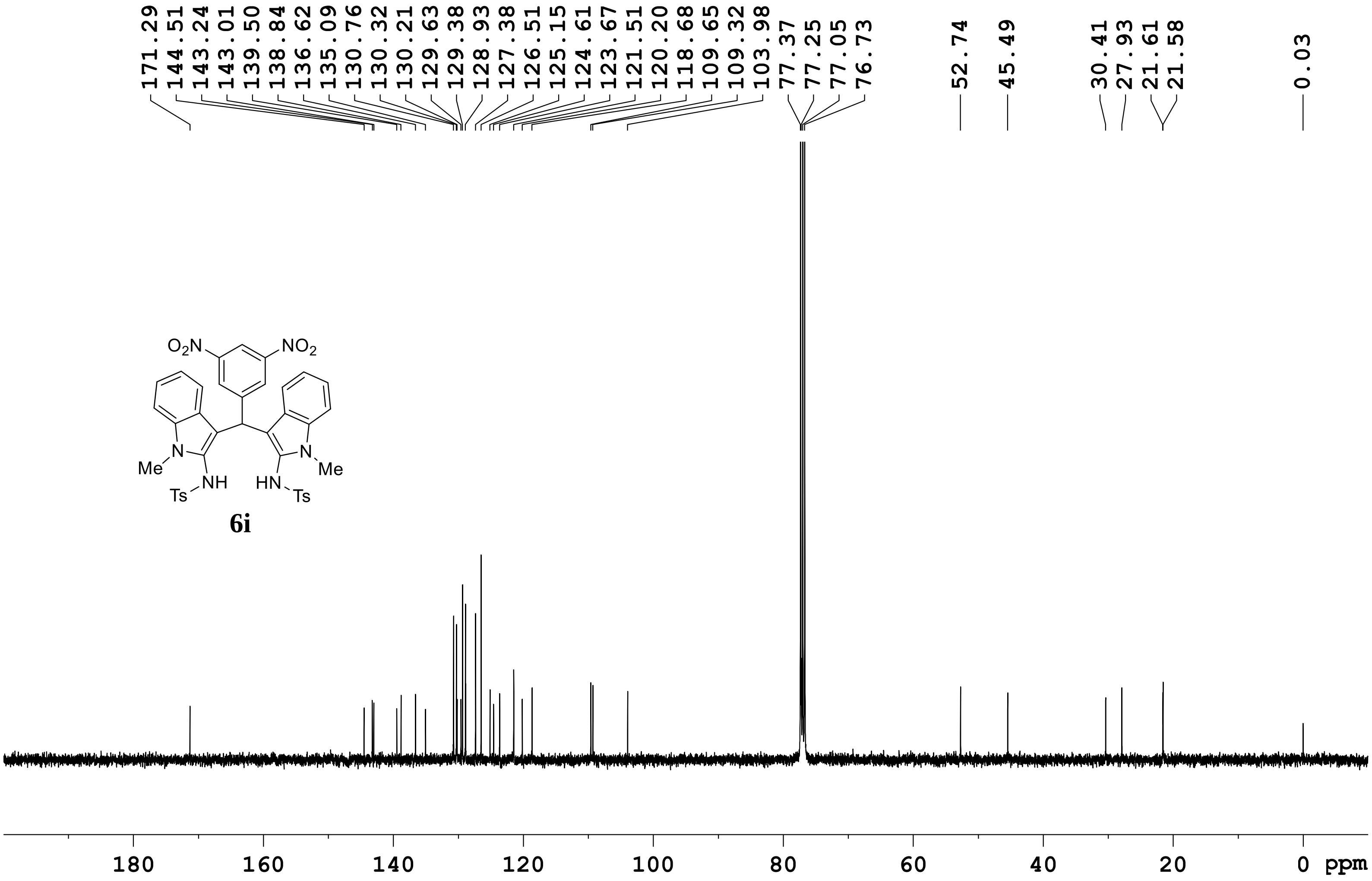

Spectra S21: ^{13}C NMR spectrum of **6i**

3,3'-(2-(Prop-2-yn-1-yloxy)phenylmethane-1,1-diyl)bis(N-tosyl-1-methylindol-2-amine)

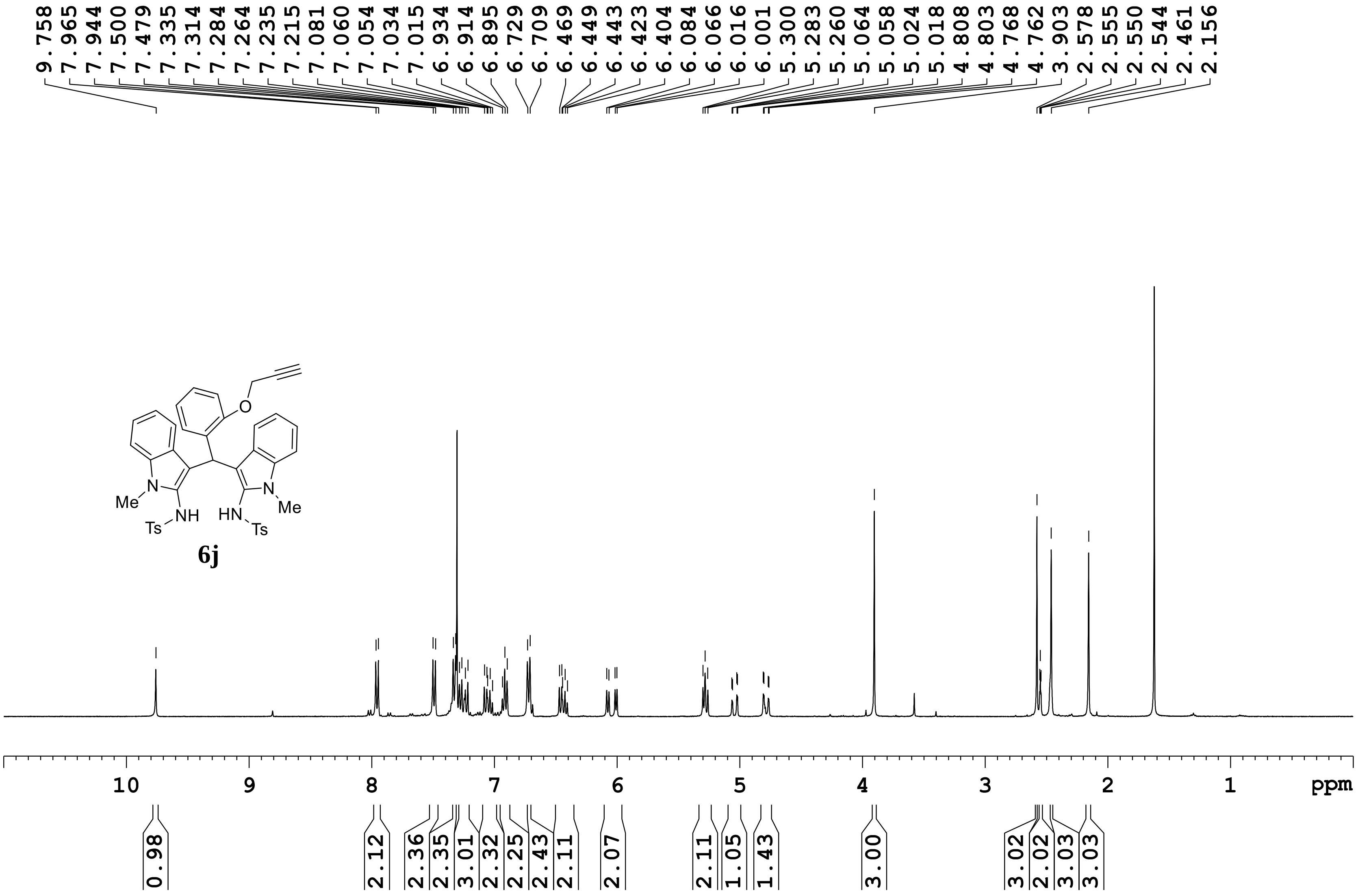

Spectra S22: ^{13}C NMR spectrum of **6j**

3,3'-(2-(Prop-2-yn-1-yloxy)phenylmethane-1,1-diyl)bis(N-tosyl-1-methylindol-2-amine)

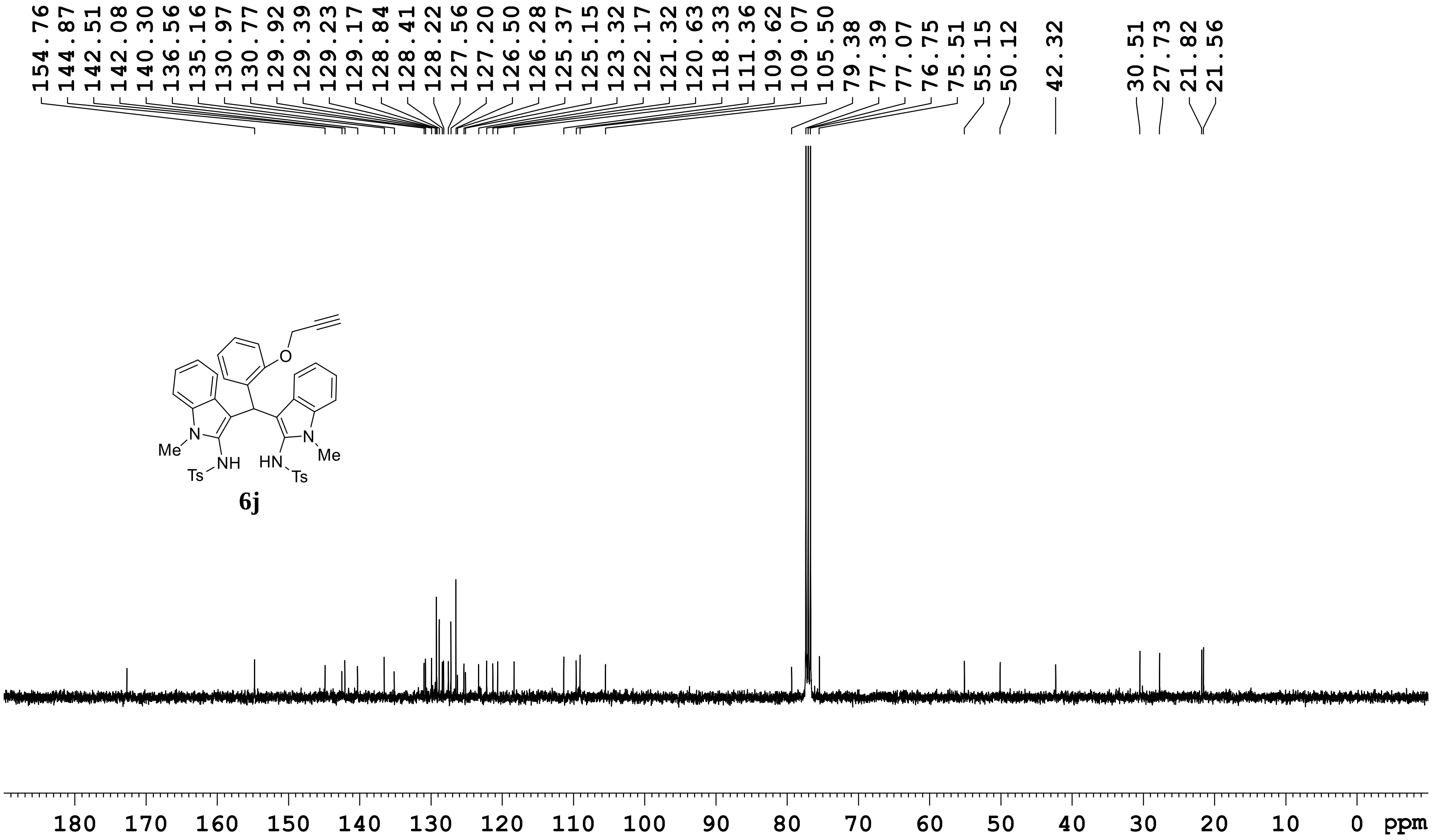

Spectra S23: ^{13}C NMR spectrum of **6j**

Scheme S3. Substrate scope for 2-sulfonamidoindoles (6k-p)

3,3'-(4-Bromophenylmethane-1,1-diyl)bis(N-tosyl-1-methylindol-2-amine)

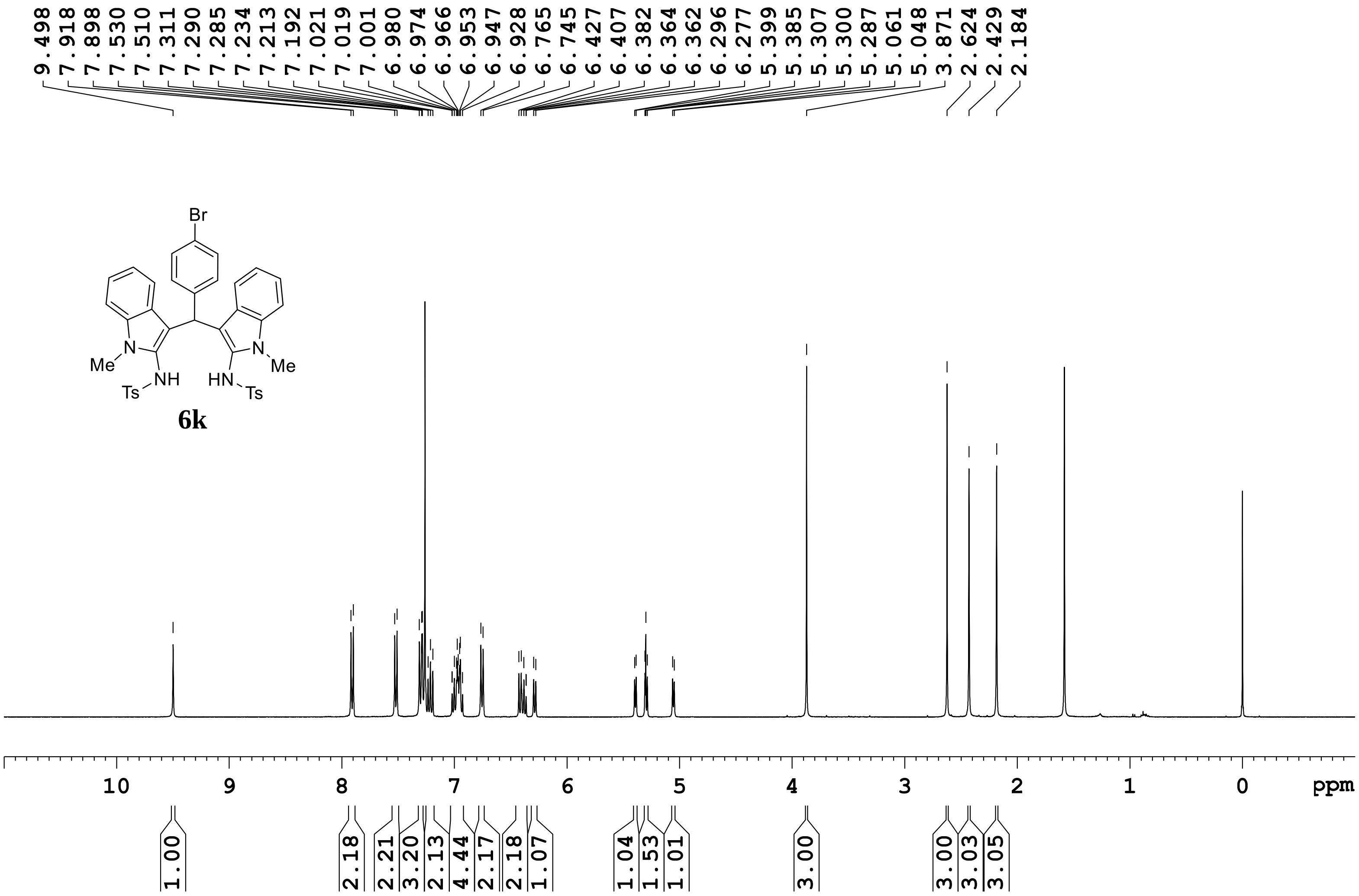

Spectra S24: ^{1}H NMR spectrum of **6k**

3,3'-(4-Bromophenylmethane-1,1-diyl)bis(N-tosyl-1-methylindol-2-amine)

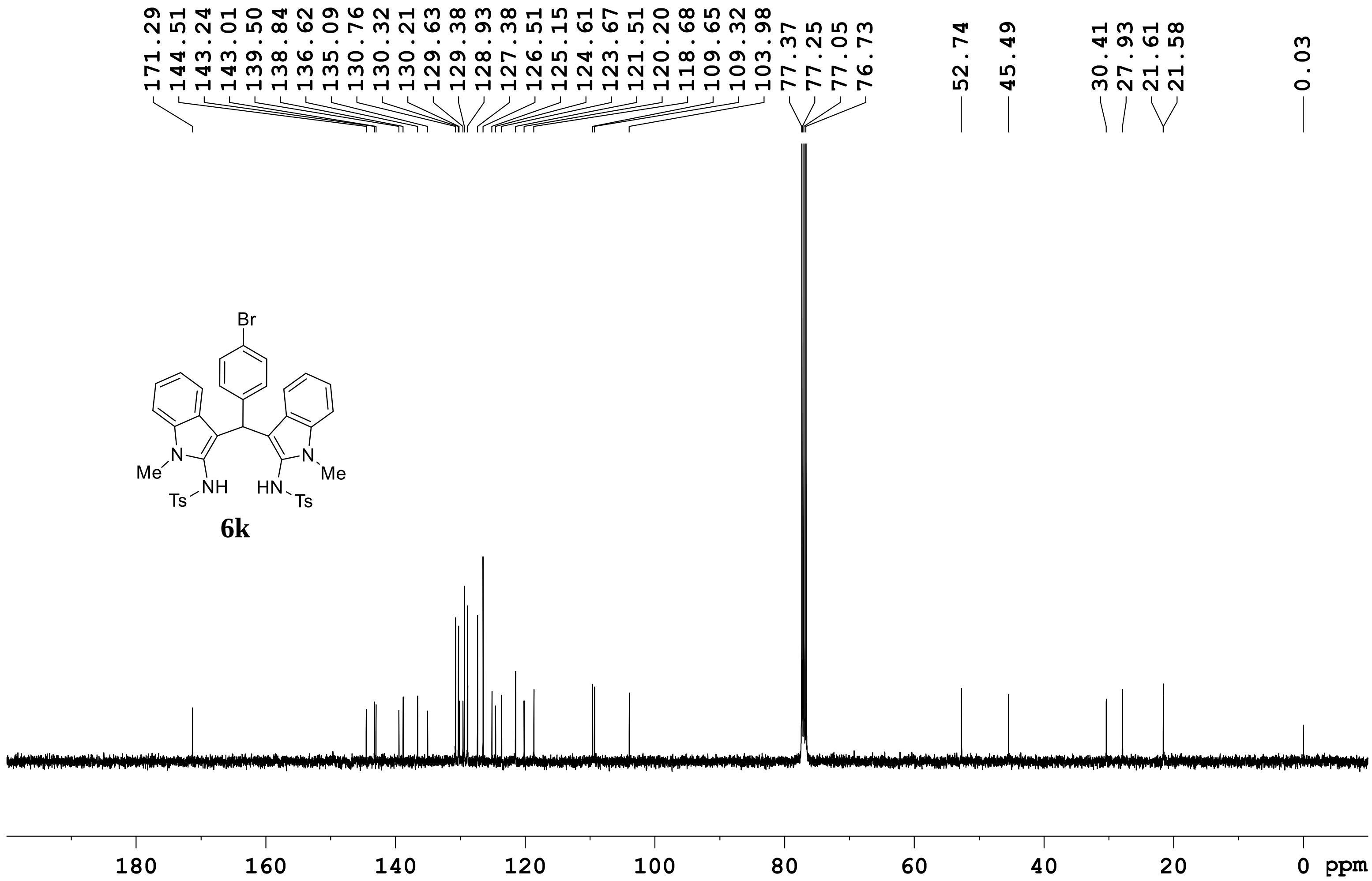

Spectra S25: ^{13}C NMR spectrum of **6k**

3,3'-(4-Tolylphenylmethane-1,1-diyl)bis(N-tosyl-1-benzylindol-2-amine)

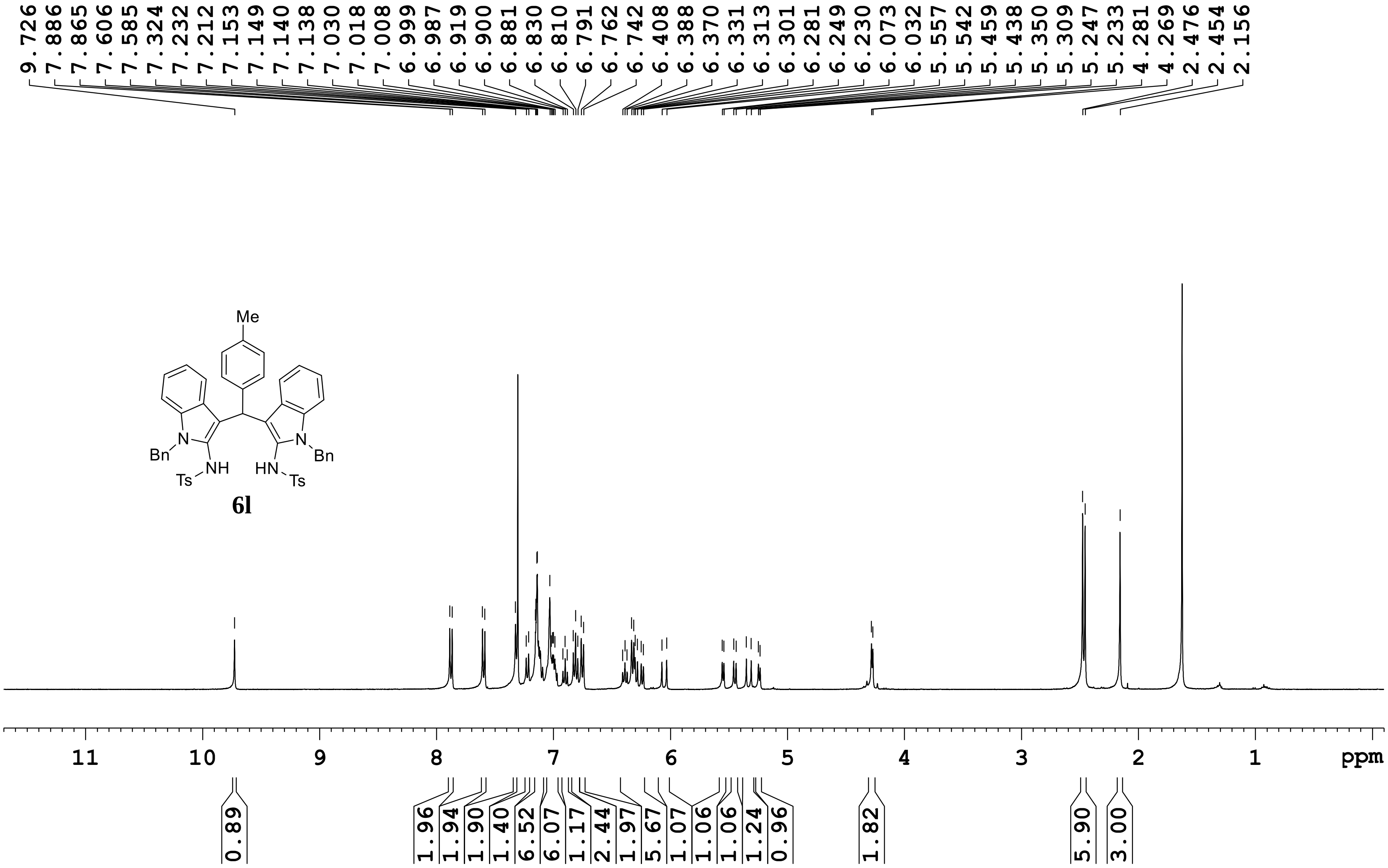

Spectra S26: ^{1}H NMR spectrum of **6l**

3,3'-(4-Tolylphenylmethane-1,1-diyl)bis(N-tosyl-1-benzylindol-2-amine)

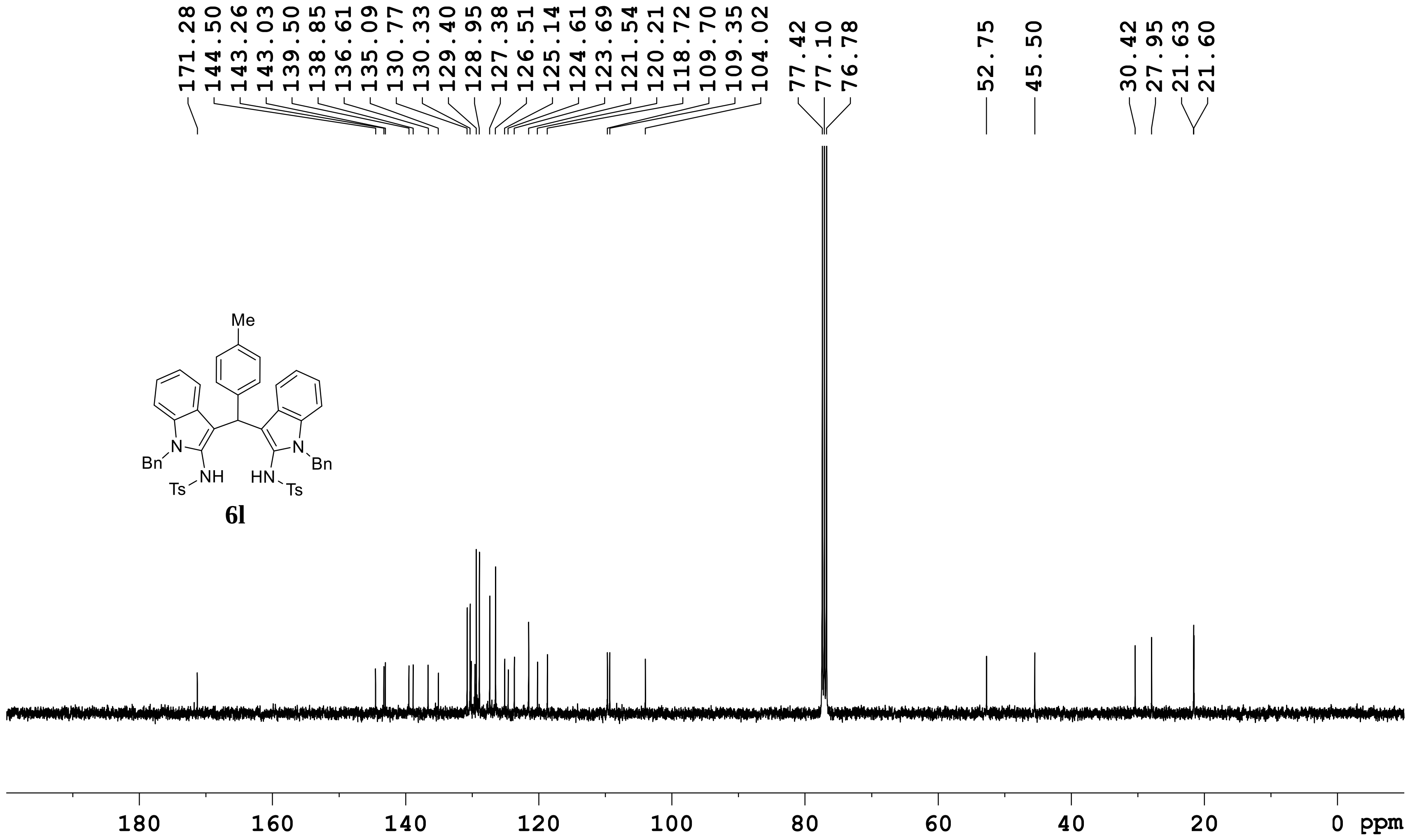

6l

Spectra S27: ^{13}C NMR spectrum of **6l**

3,3'-(4-Bromophenylmethane-1,1-diyl)bis(N-tosyl-1-ethyl-5-methoxyindol-2-amine)

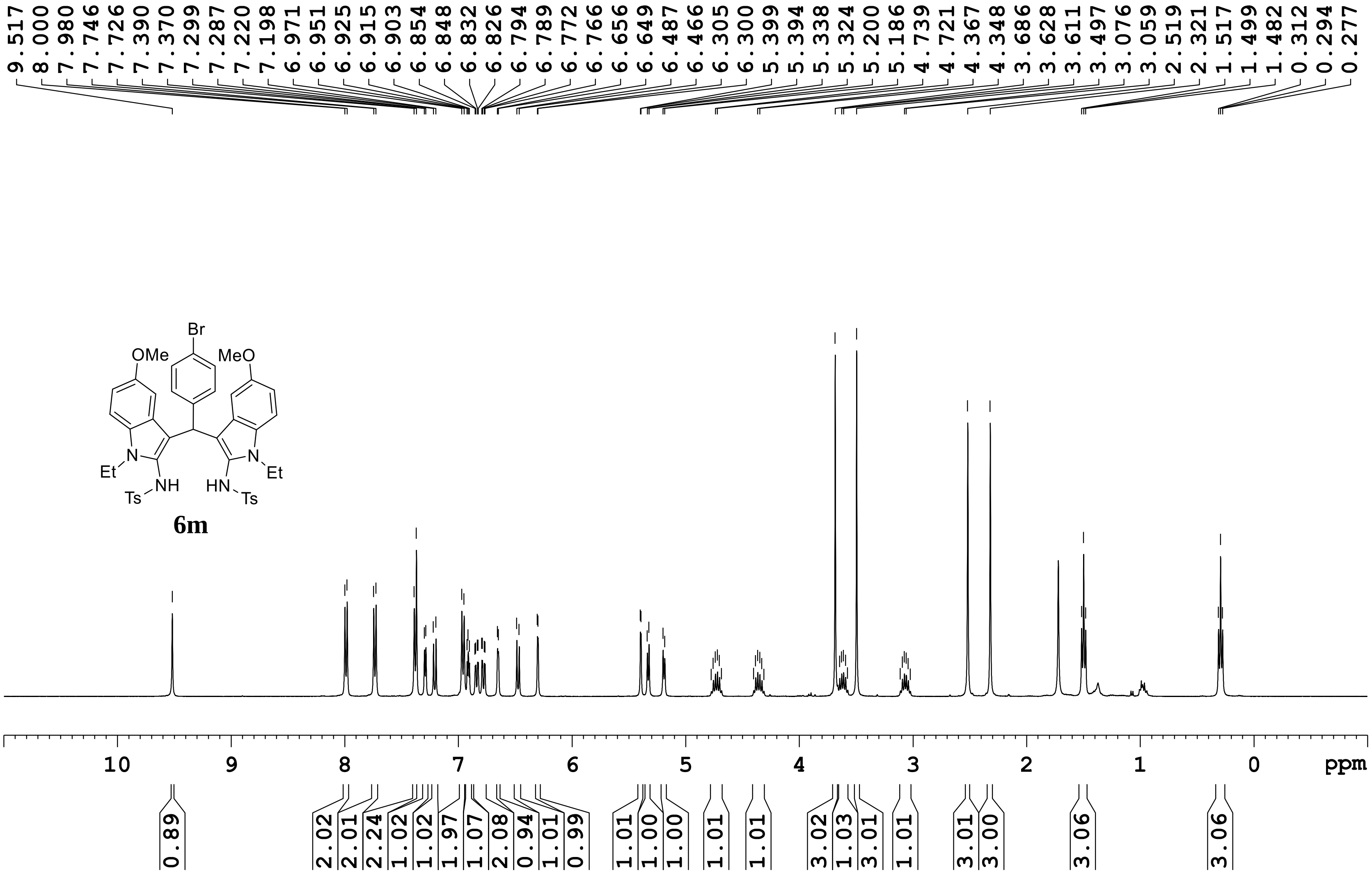

Spectra S28: ^{1}H NMR spectrum of **6m**

3,3'-(4-Bromophenylmethane-1,1-diyl)bis(N-tosyl-1-ethyl-5-methoxyindol-2-amine)

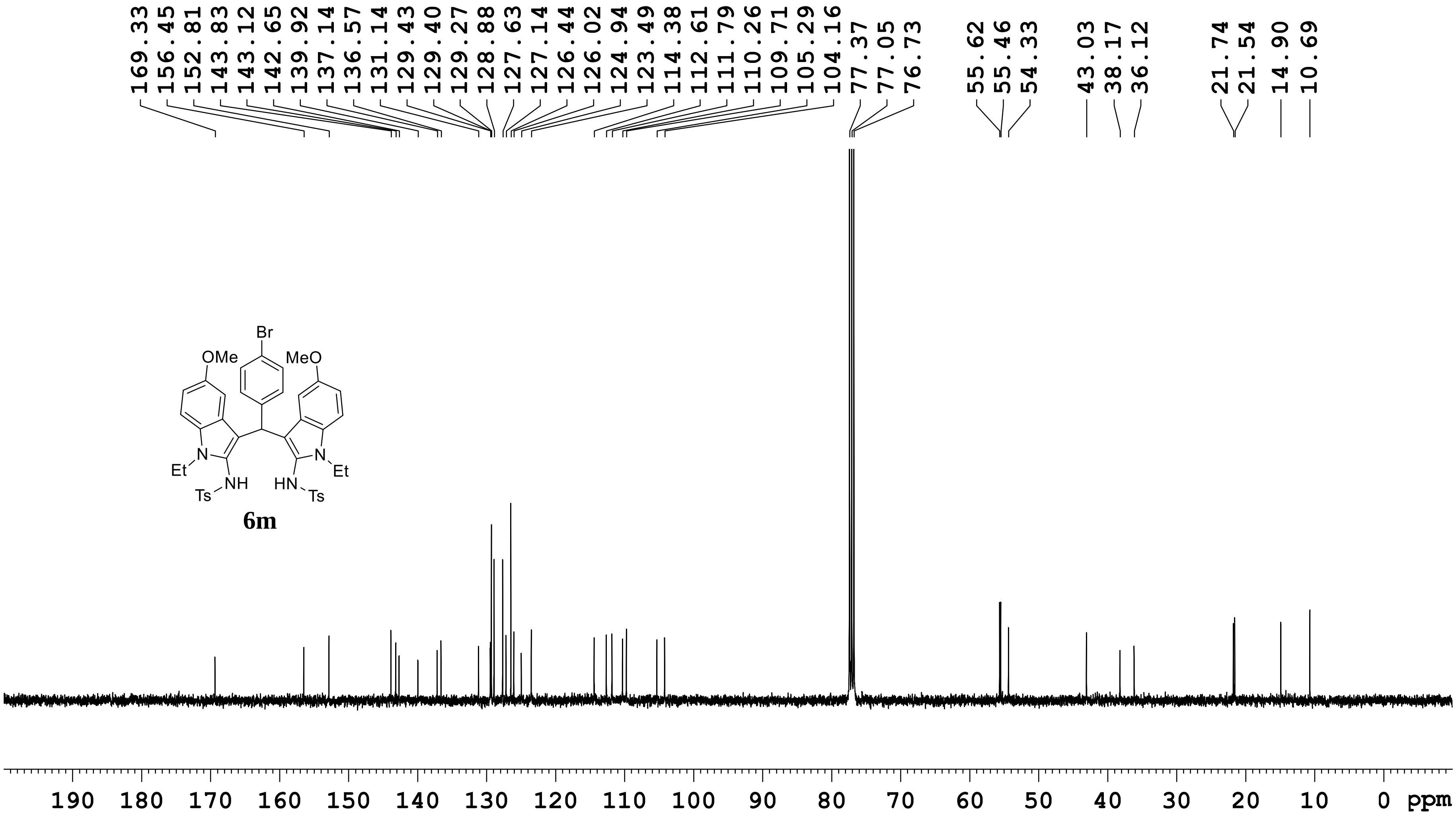

Spectra S29: ^{13}C NMR spectrum of **6m**

3,3'-(4-Bromophenylmethane-1,1-diyl)bis(N-tosyl-1-methyl-5-bromoindol-2-amine)

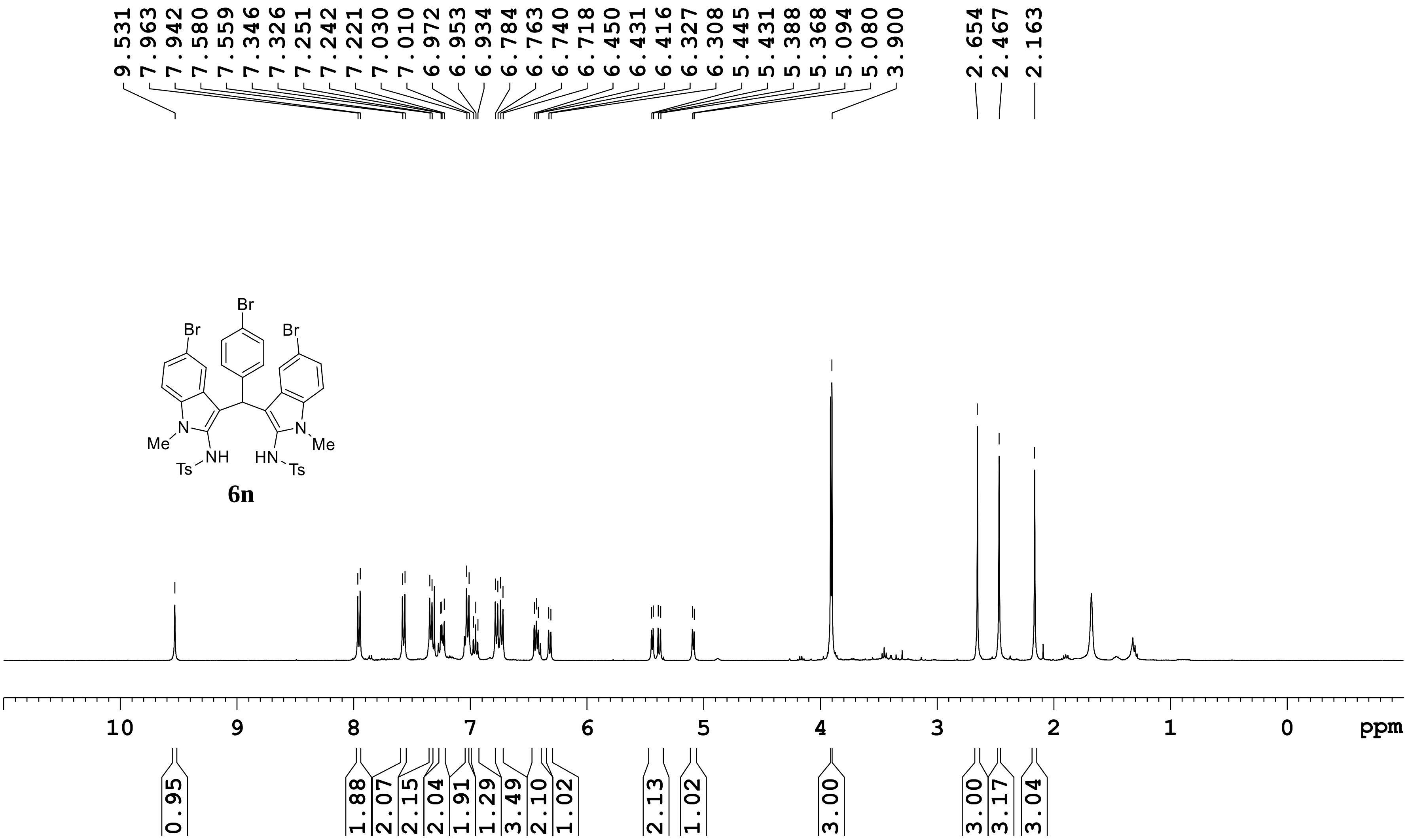

Spectra S30: ^{1}H NMR spectrum of **6n**

3,3'-(4-Bromophenylmethane-1,1-diyl)bis(N-tosyl-1-methyl-5-bromoindol-2-amine)

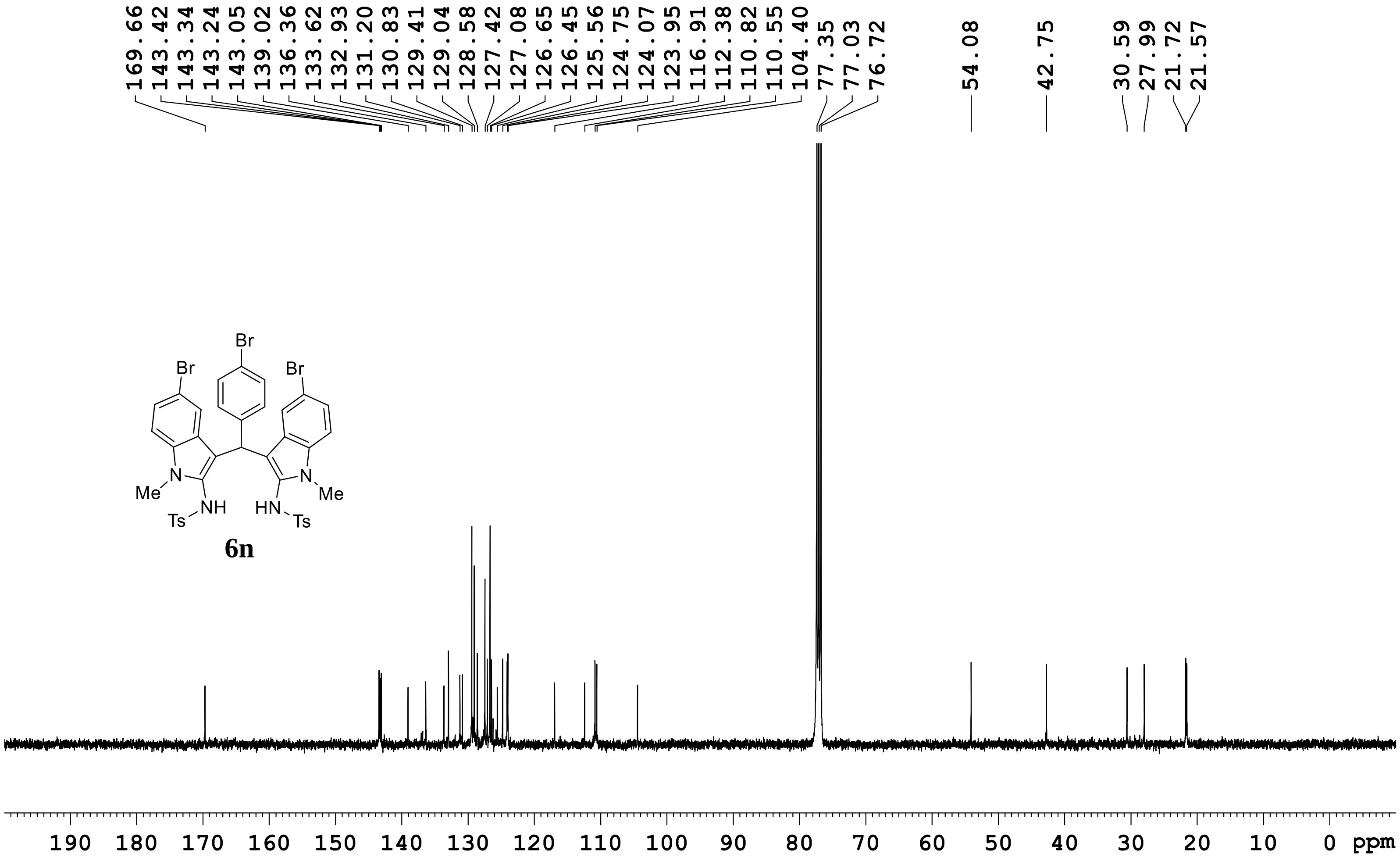

6n

Spectra S31: ^{13}C NMR spectrum of **6n**

3,3'-(4-Bromophenylmethane-1,1-diyl)bis(N-tosyl-1-ethyl-6-bromoindol-2-amine)

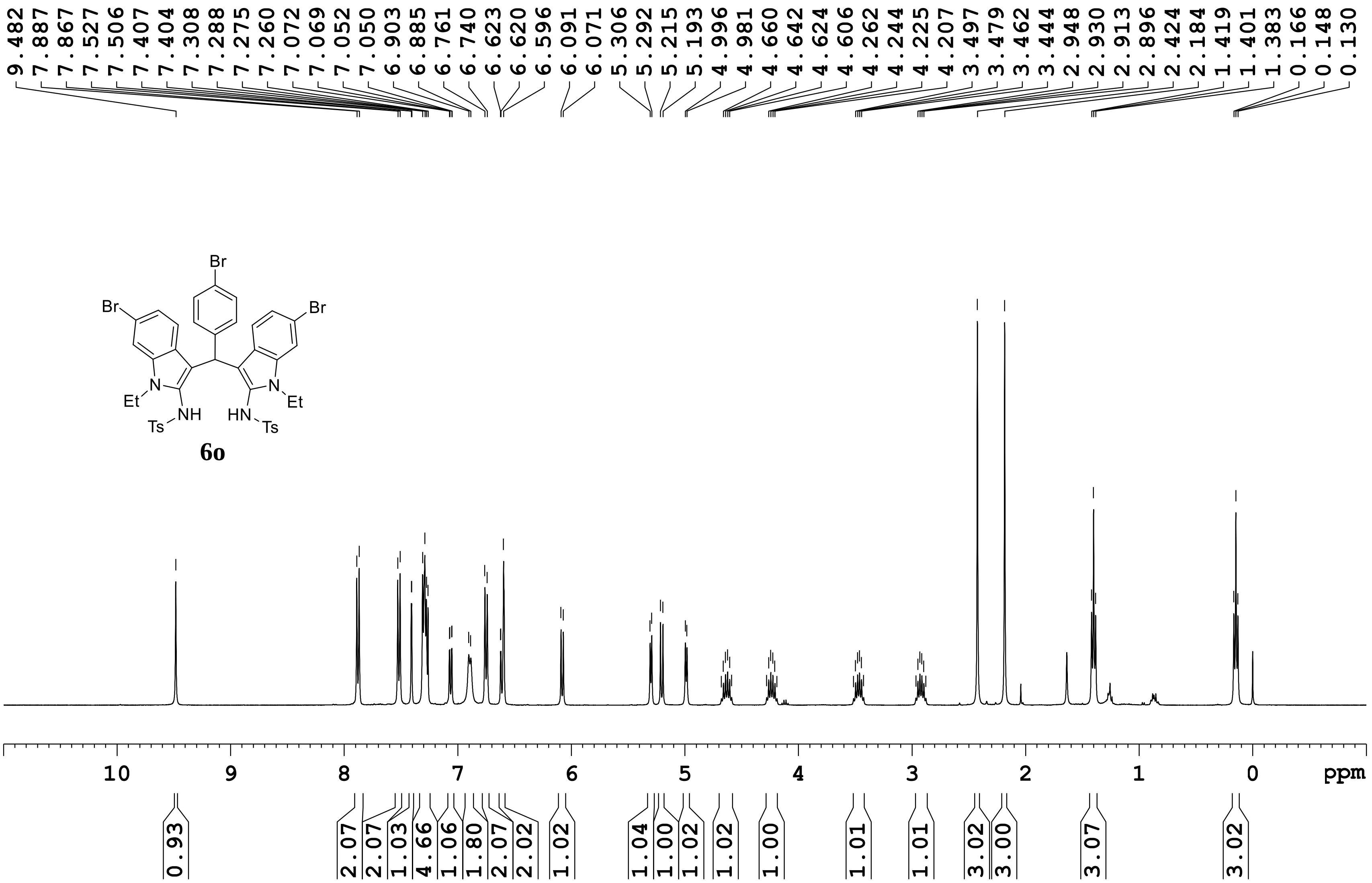

Spectra S32: ^{1}H NMR spectrum of **6o**

3,3'-(4-Bromophenylmethane-1,1-diyl)bis(N-tosyl-1-ethyl-6-bromoindol-2-amine)

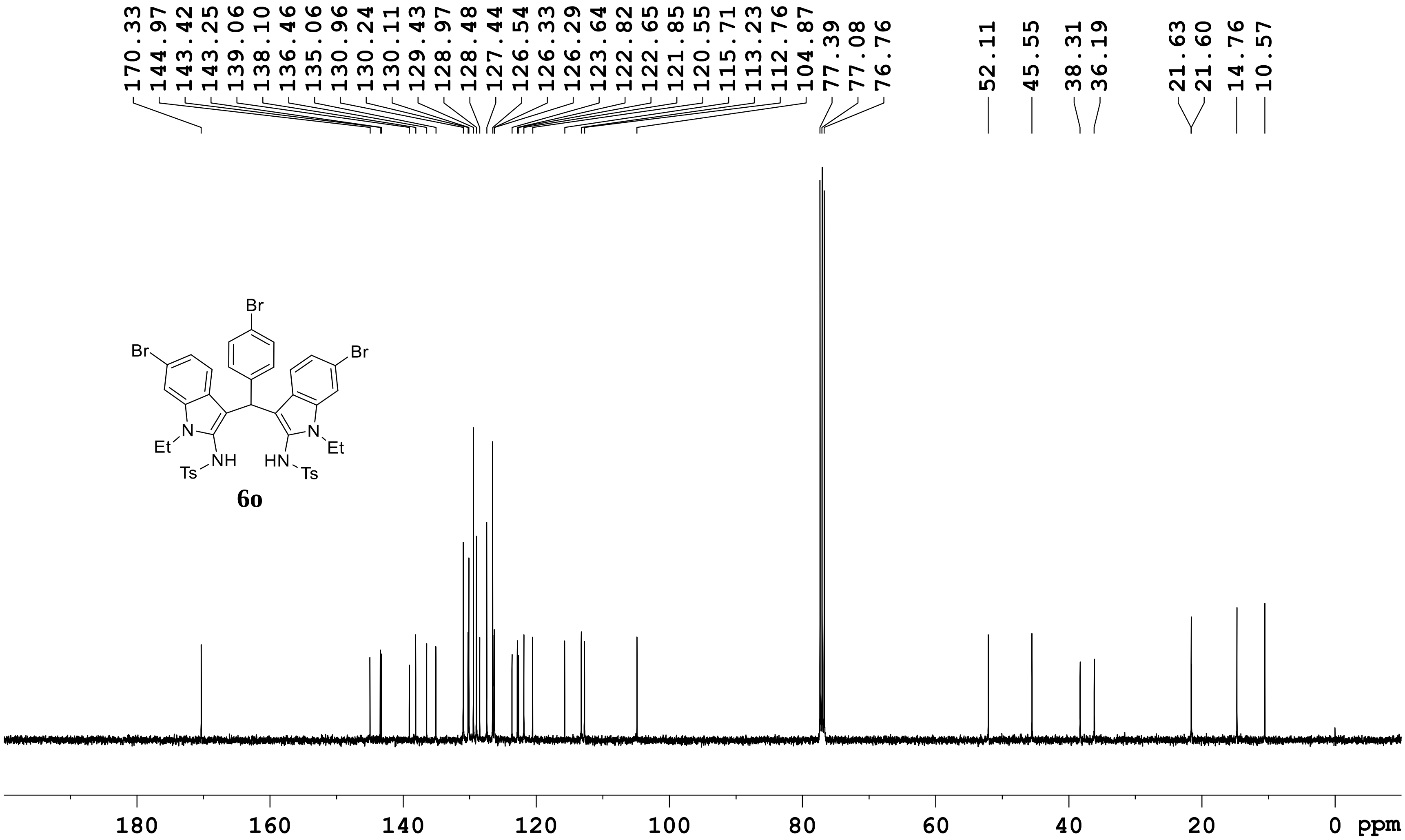

Spectra S33: ^{13}C NMR spectrum of **6o**

***N*-(4-(Bromomethyl)benzyl)-N-(1-ethyl-3-((1-ethyl-2-((4-tolyl)sulfonamido)-1H-indol-3-yl)(*p*-bromophenyl)methyl)-1H-indol-2-yl)-4-tolylsulfonamide**

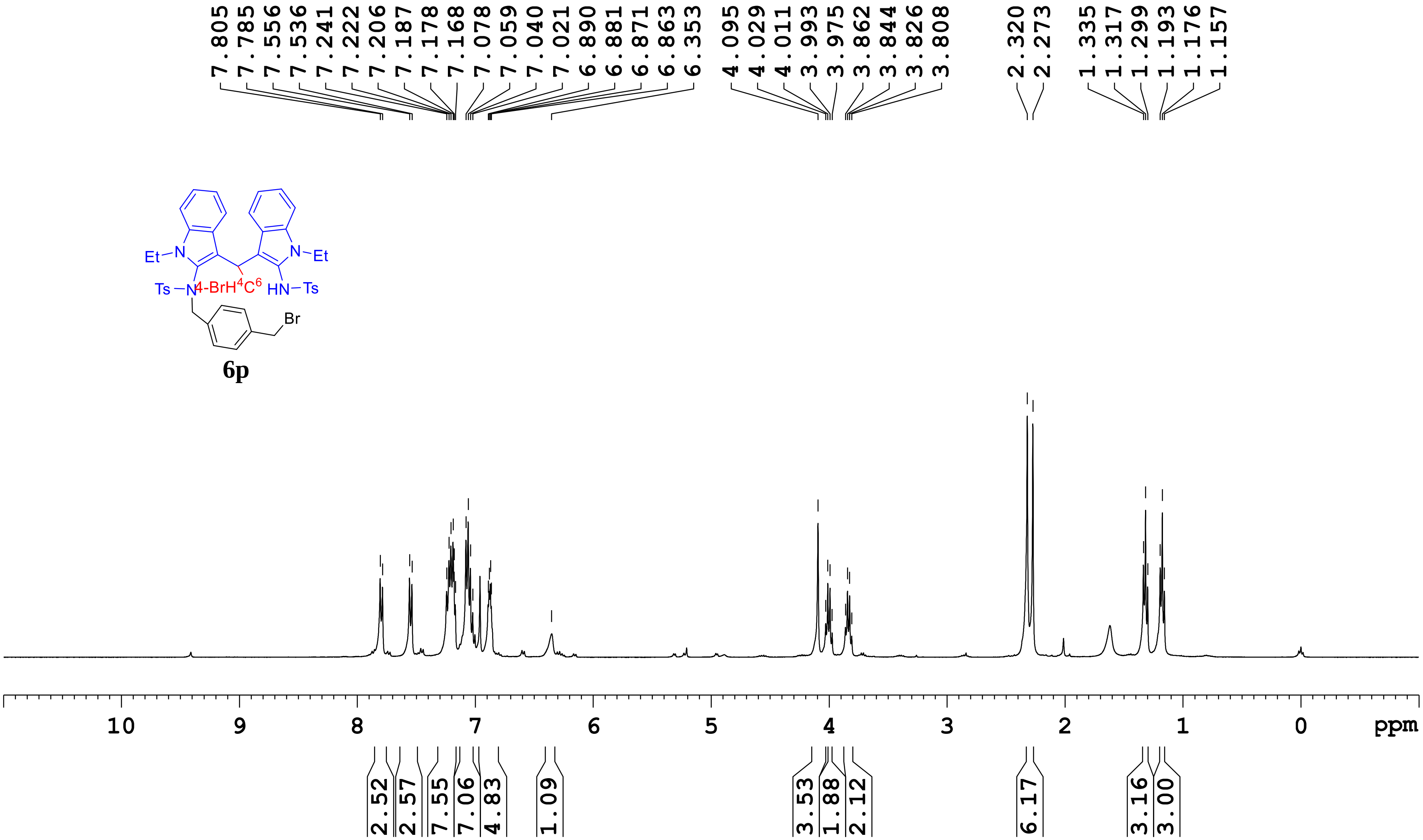

Spectra S34: ^{1}H NMR spectrum of **6p**

N-(4-(Bromomethyl)benzyl)-N-(1-ethyl-3-((1-ethyl-2-((4-tolyl)sulfonamido)-1H-indol-3-yl)(*p*-bromophenyl)methyl)-1H-indol-2-yl)-4-tolylsulfonamide

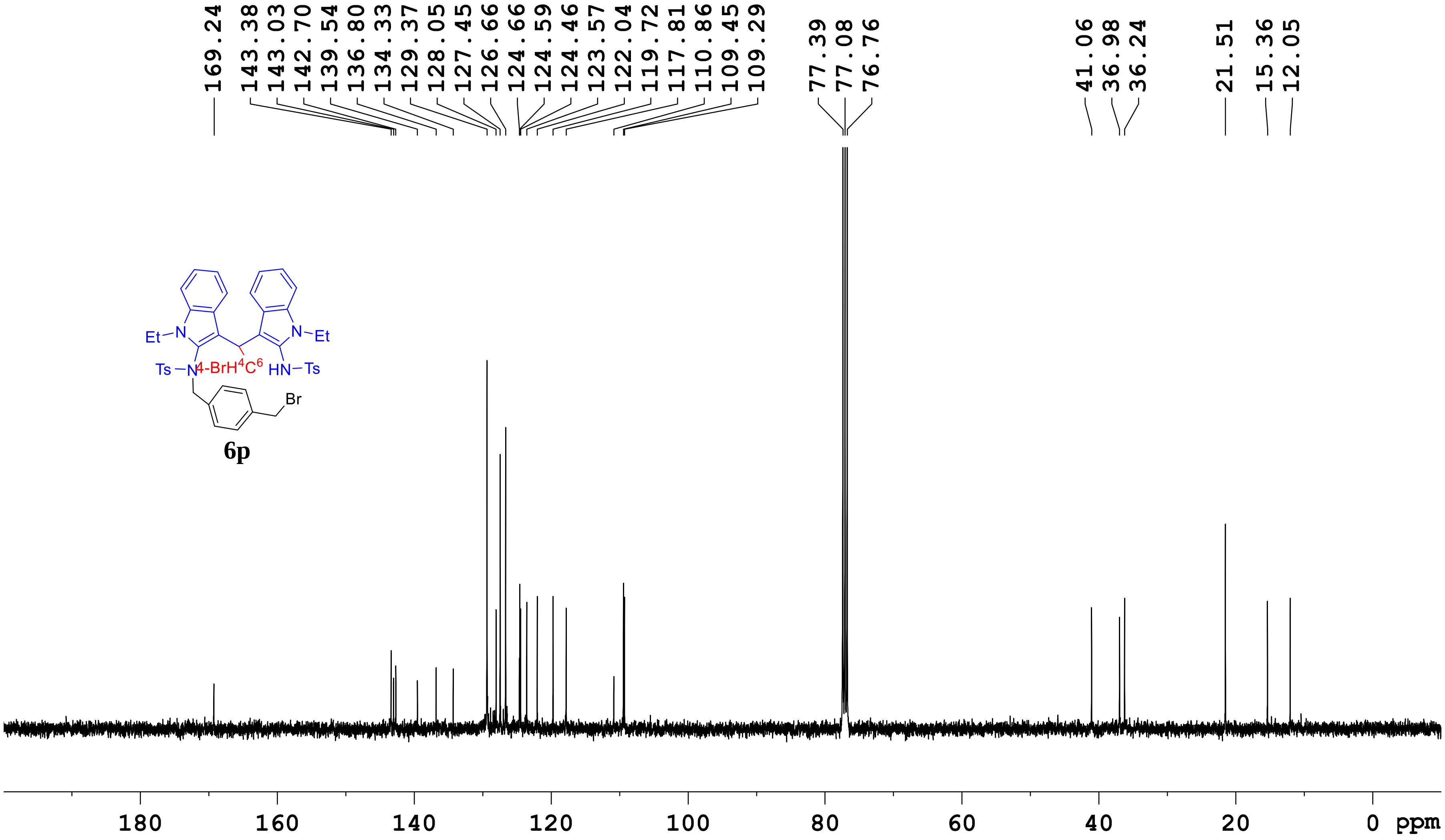

Spectra S35: ^{13}C NMR spectrum of **6p**

Scheme S4. Substrate scope for 2-amine-3-arylindoles (7a-g)

(3E)-N-Tosyl-3-[(indolyl)methylidene]-2,3-dihydro-1-methylindol-2-amine

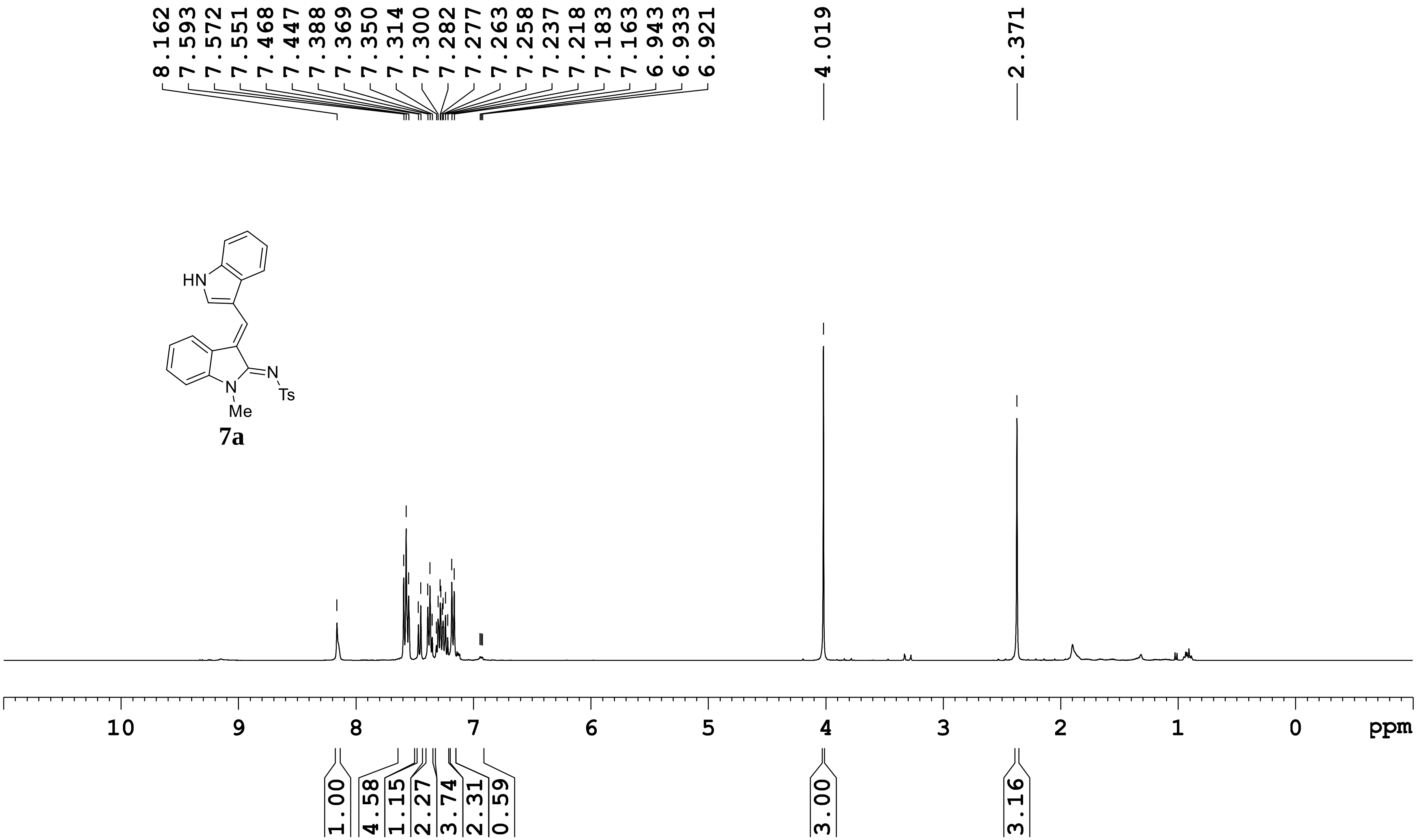

Spectra S36: ^{1}H NMR spectrum of **7a**

(3E)-N-Tosyl-3-[(indolyl)methylidene]-2,3-dihydro-1-methylindol-2-amine

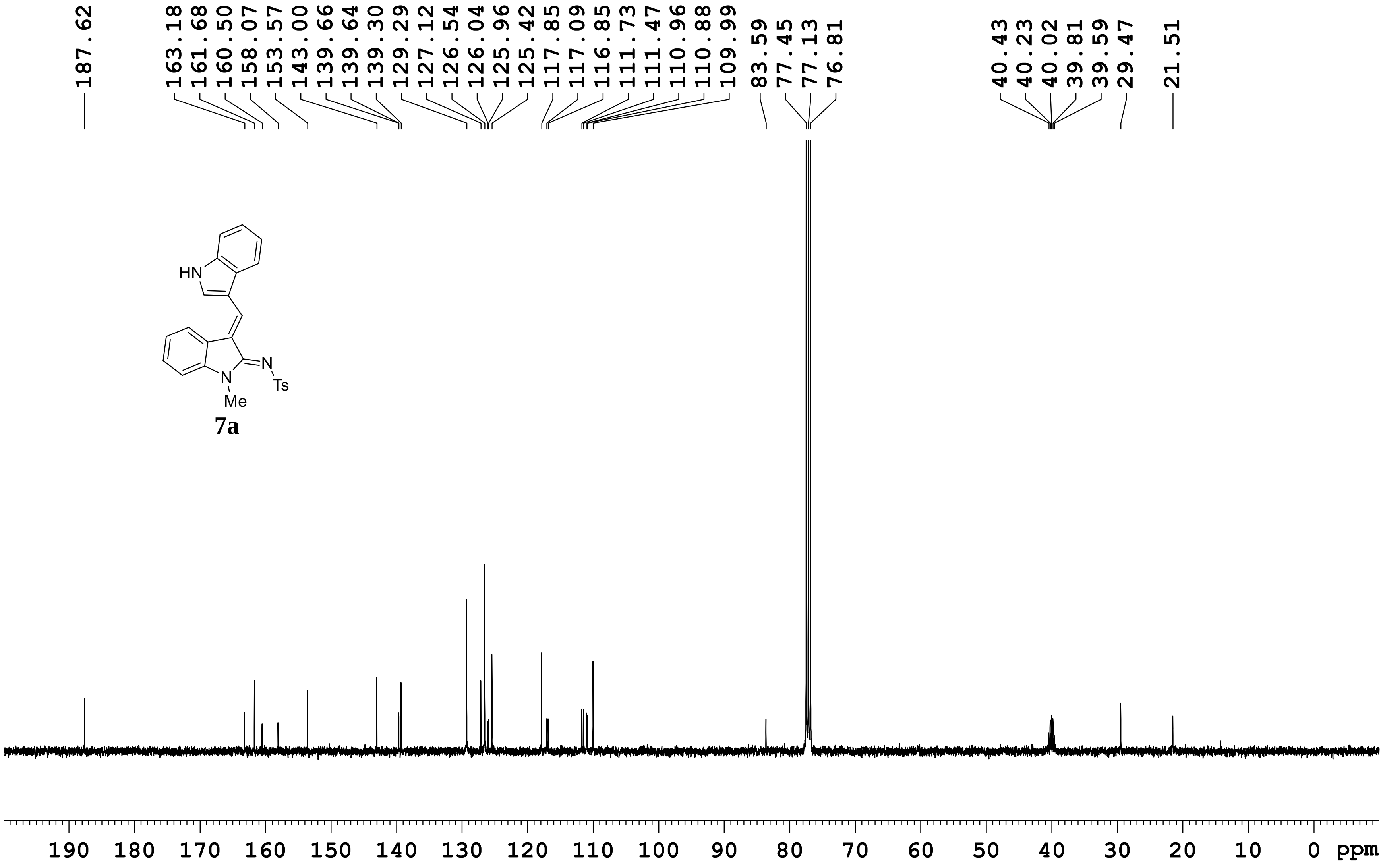

Spectra S37: ^{13}C NMR spectrum of **7a**

(3*E*)-N-Tosyl-3-[(naphthyl)methylidene]-2,3-dihydro-1-methylindol-2-amine

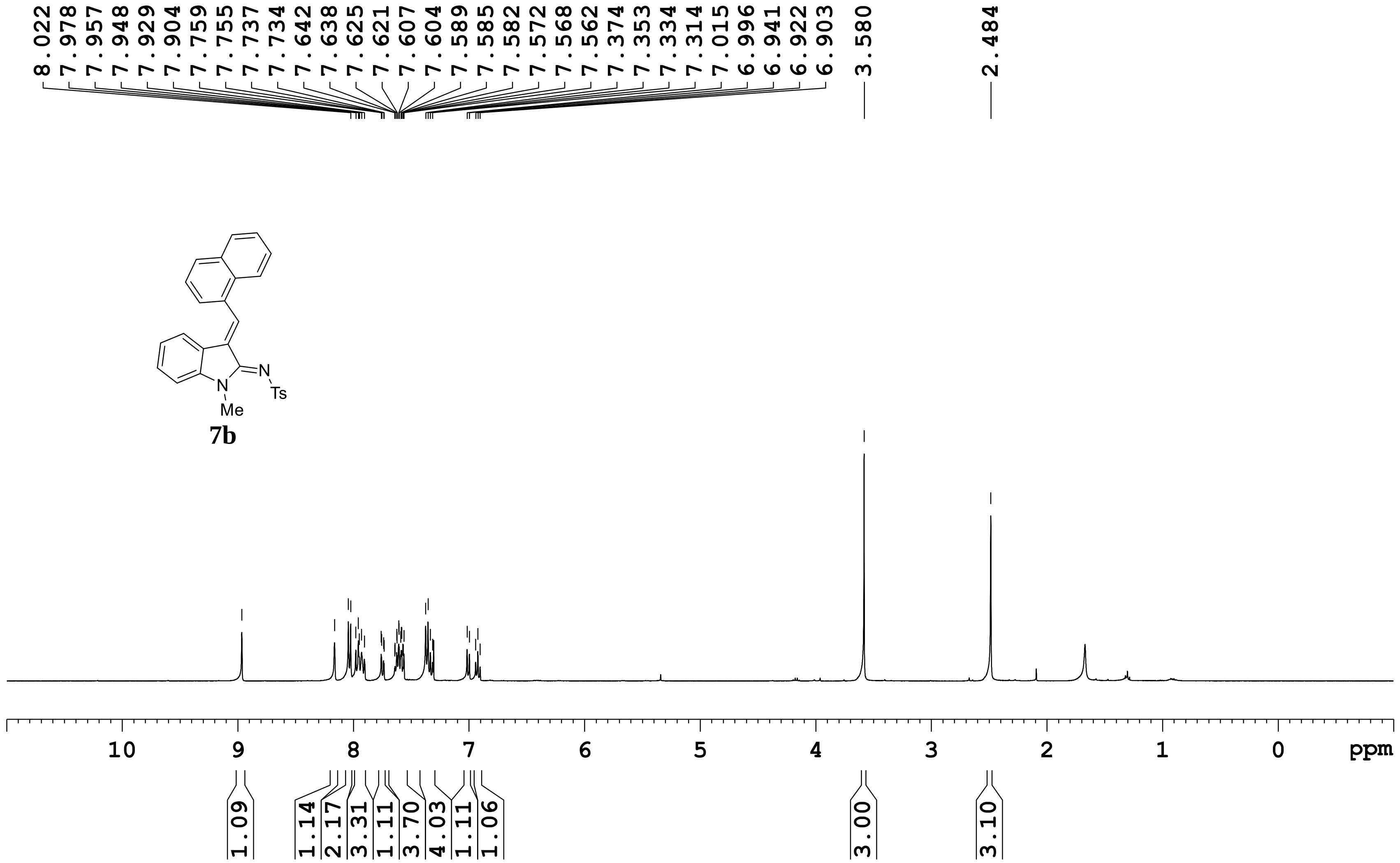

Spectra S38: ^{1}H NMR spectrum of **7b**

(3*E*)-N-Tosyl-3-[(naphthyl)methylidene]-2,3-dihydro-1-methylindol-2-amine

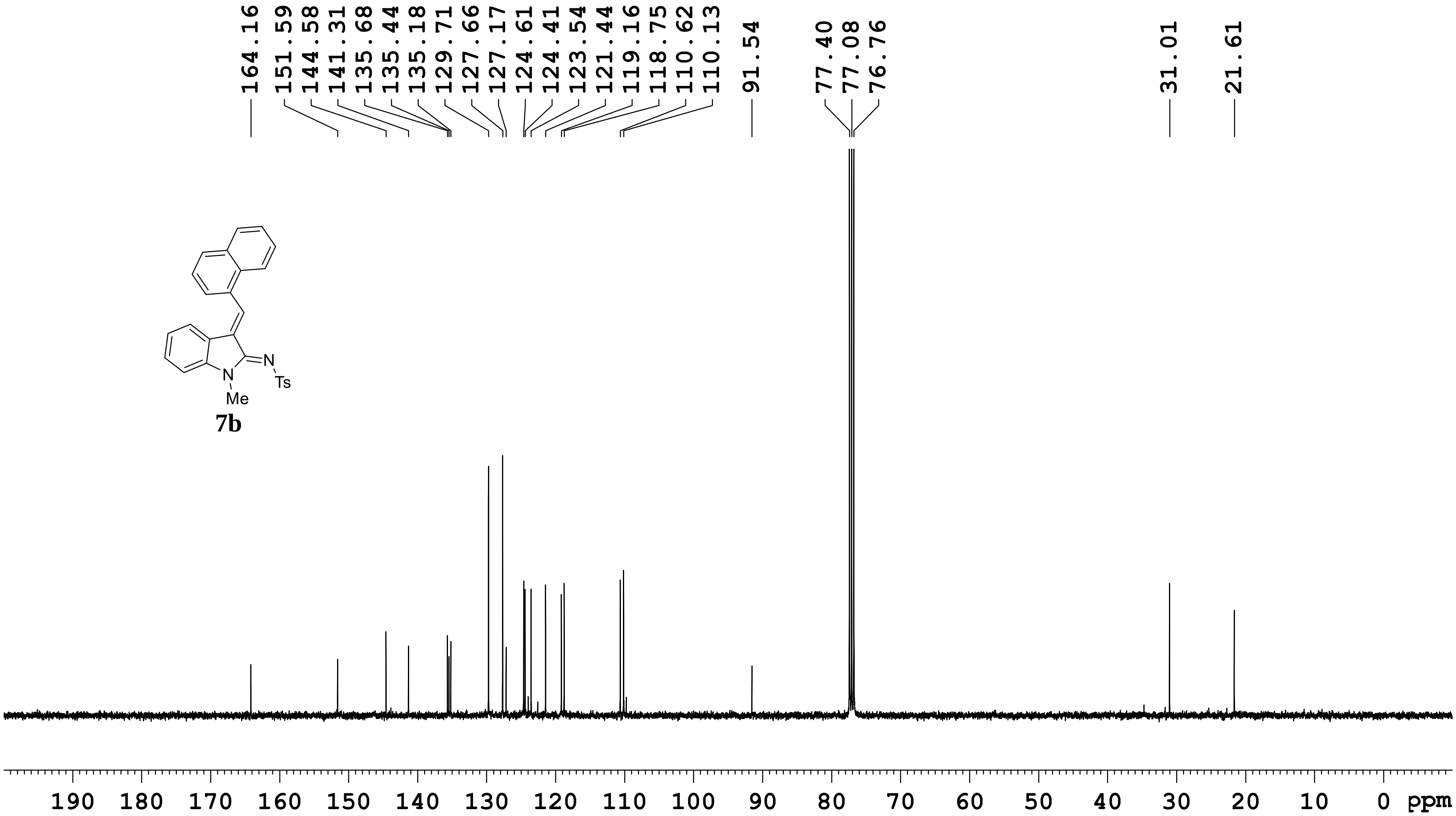

Spectra S39: ^{1}H NMR spectrum of **7b**

(3*E*)-N-Tosyl-3-[(anthryl)methylidene]-2,3-dihydro-1-methylindol-2-amine

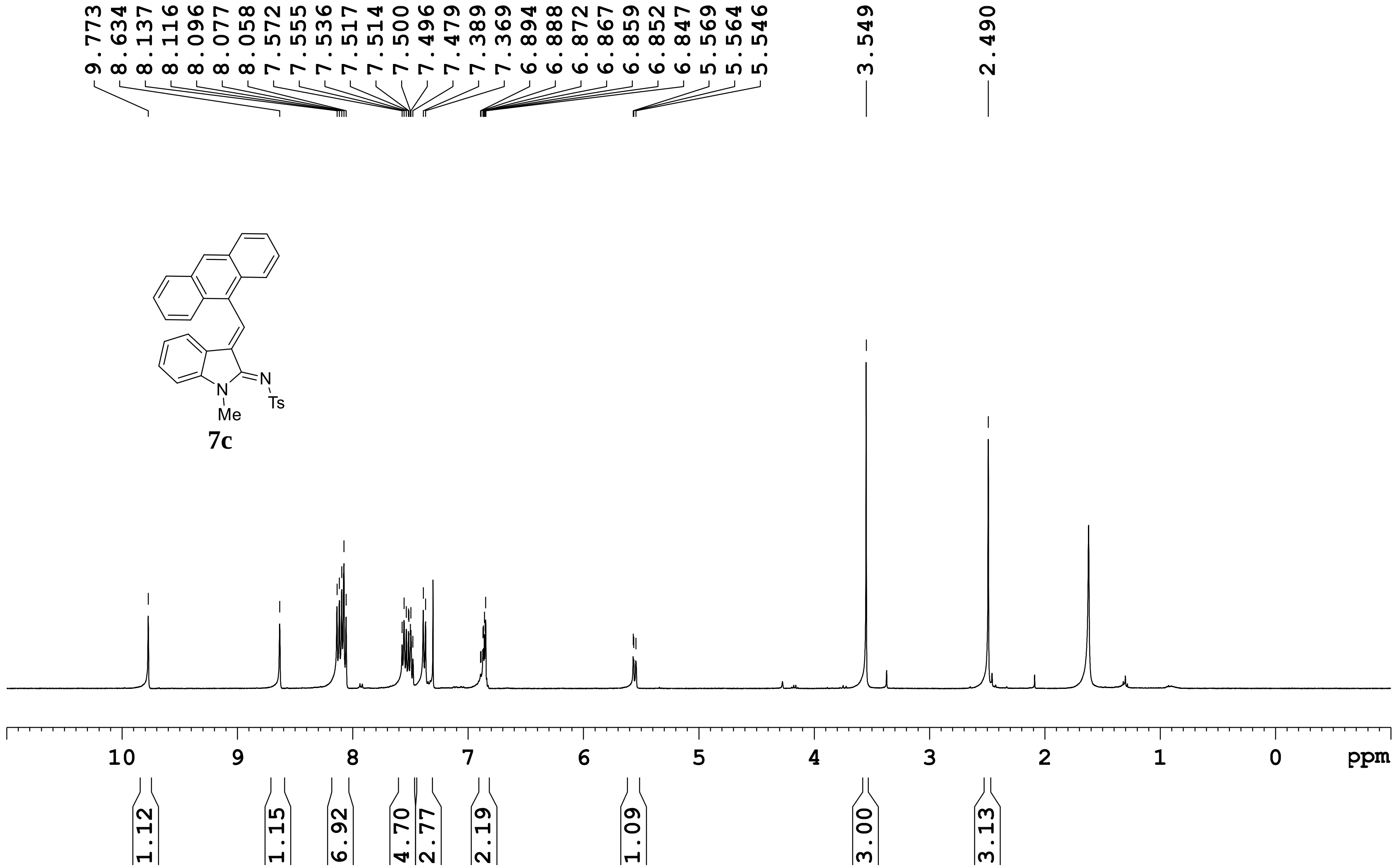

Spectra S40: ^{1}H NMR spectrum of **7c**

(3*E*)-N-Tosyl-3-[(anthryl)methylidene]-2,3-dihydro-1-methylindol-2-amine

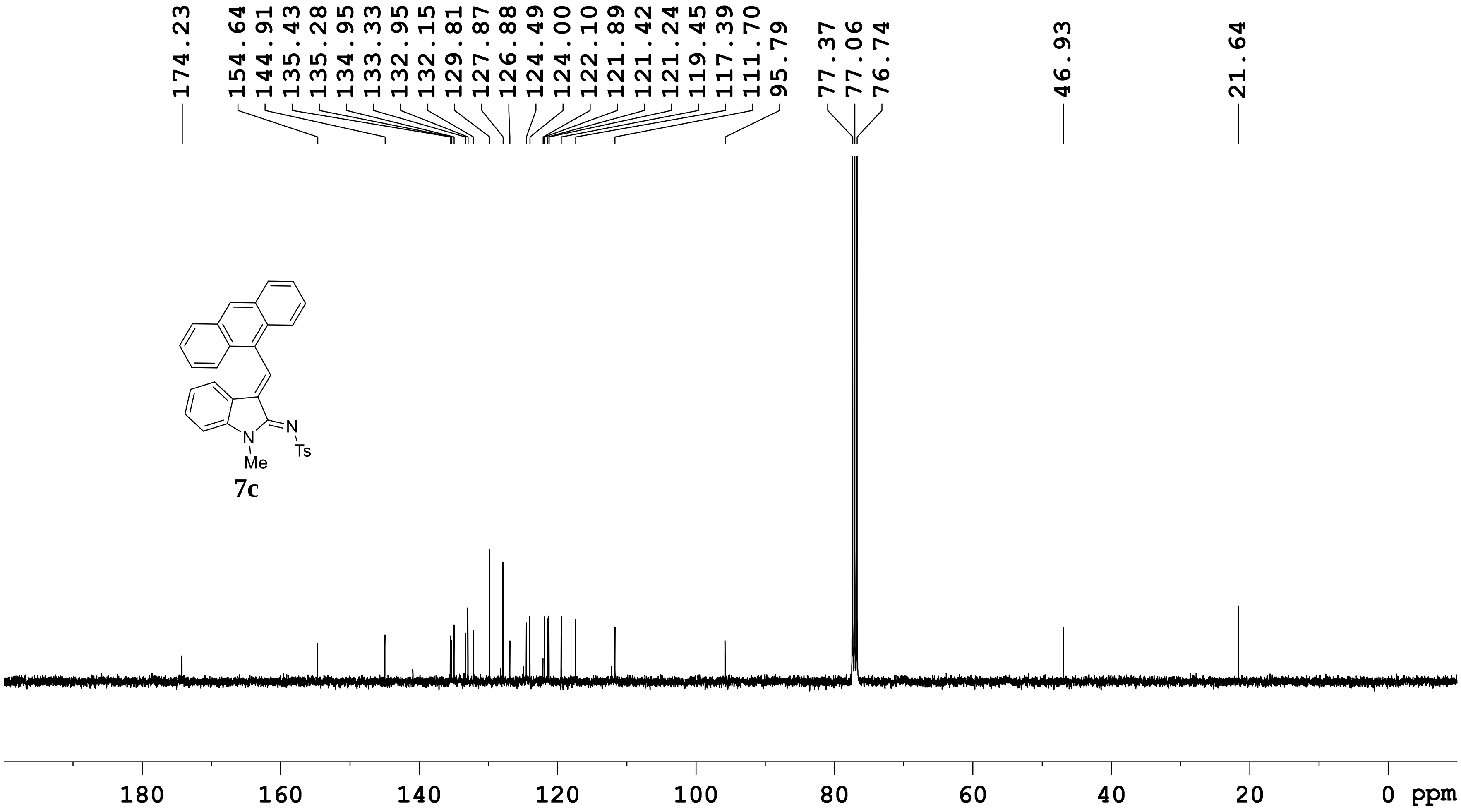

Spectra S41: ^{1}H NMR spectrum of **7c**

(3*E*)-N-Tosyl-3-[(4-methylphenyl)methylidene]-2,3-dihydro-1H-indol-2-amine

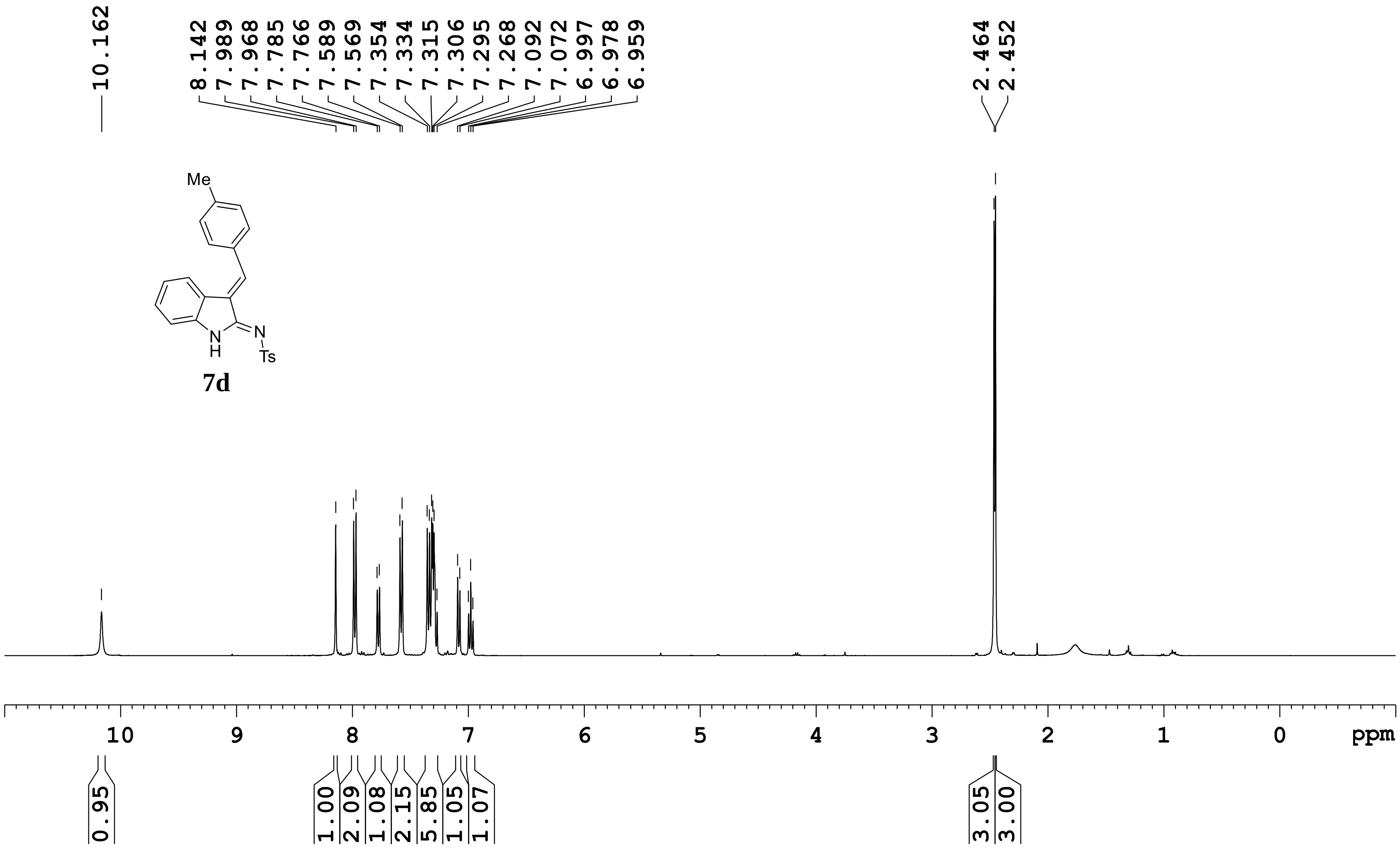

Spectra S42: ^{1}H NMR spectrum of **7d**

(3*E*)-N-Tosyl-3-[(4-methylphenyl)methylidene]-2,3-dihydro-1H-indol-2-amine

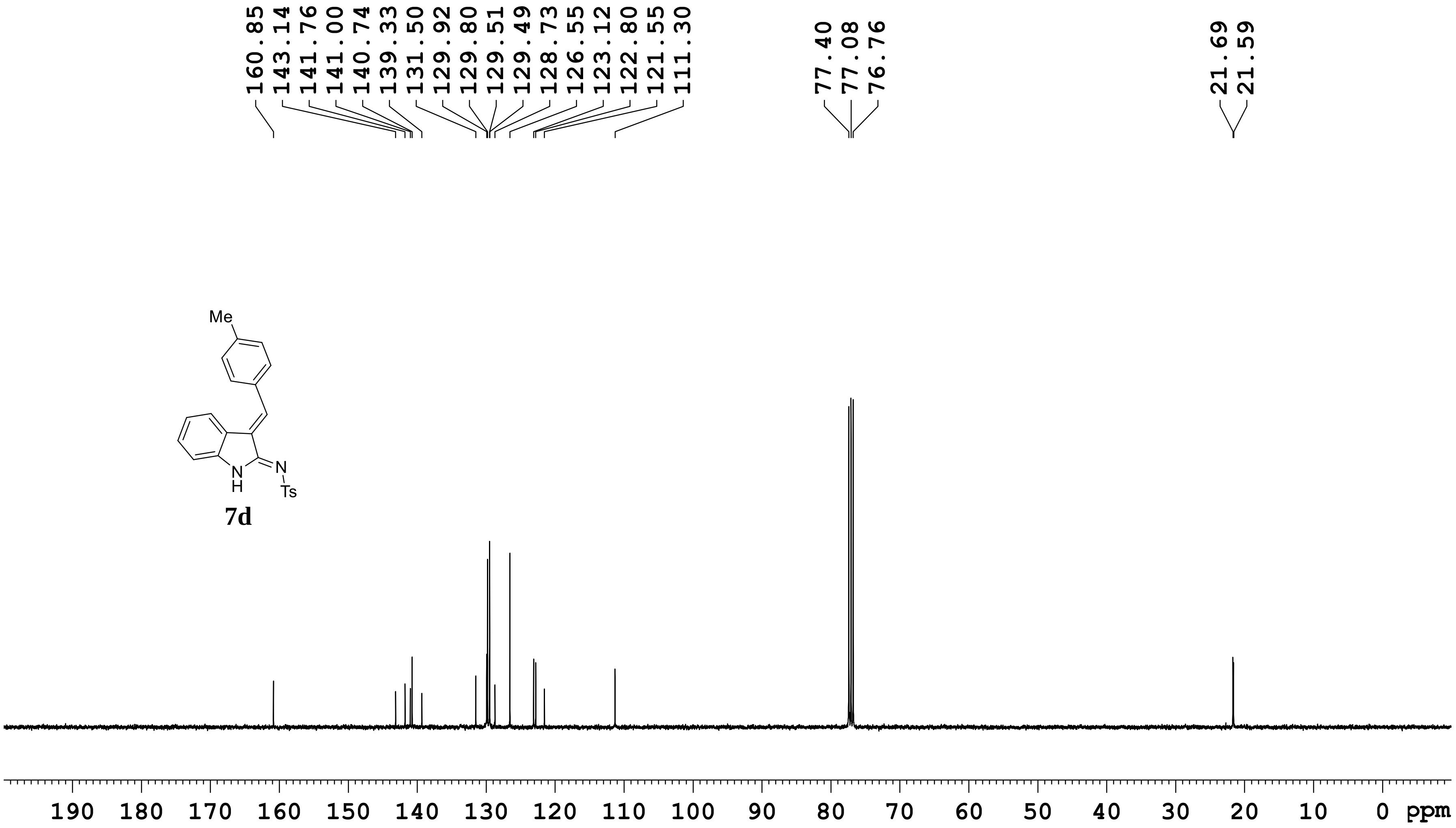

Spectra S43: ^{13}C NMR spectrum of **7d**

(3*E*)-N-Tosyl-3-[(4-bromophenyl)methylidene]-5-fluoro-2,3-dihydro-1-methylindol-2-amine

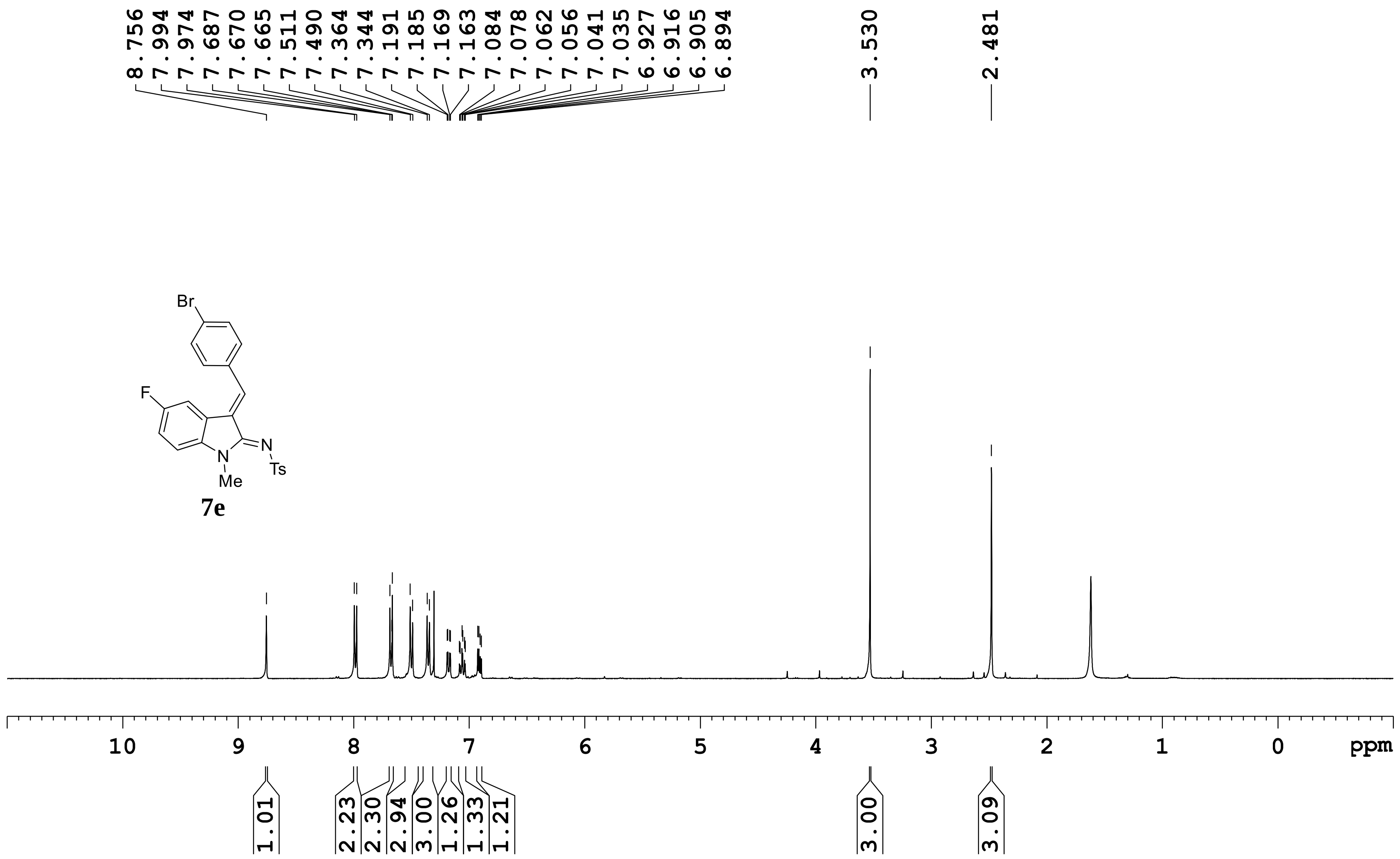

7e

Spectra S44: ^{1}H NMR spectrum of **7e**

(3*E*)-N-Tosyl-3-[(4-bromophenyl)methylidene]-5-fluoro-2,3-dihydro-1-methylindol-2-amine

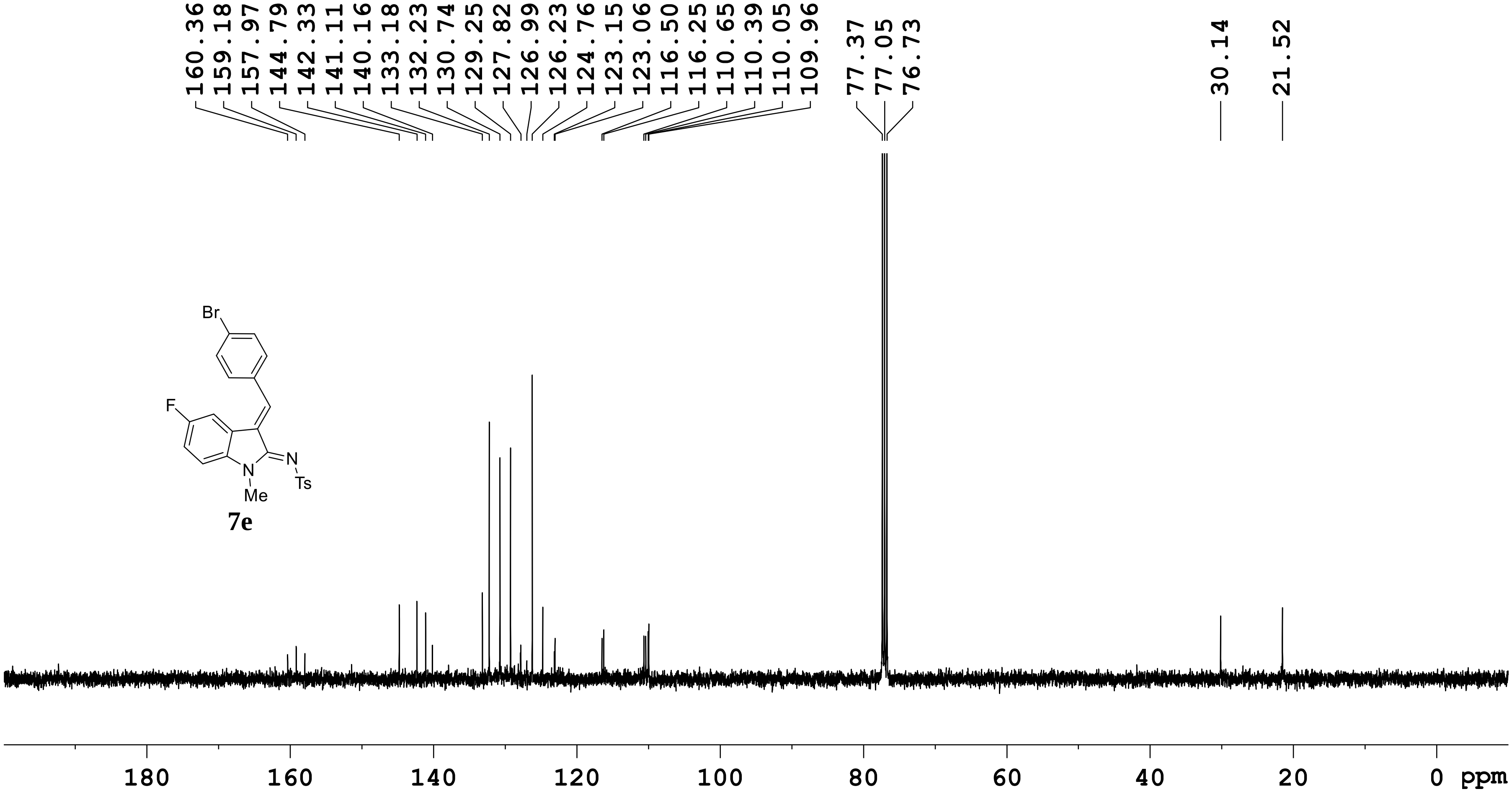

Spectra S45: ^{13}C NMR spectrum of **7e**

(3*E*)-N-Tosyl-3-[(3,5-dinitrophenyl)methylidene]-5-fluoro-2,3-dihydro-1-methylindol-2-amine

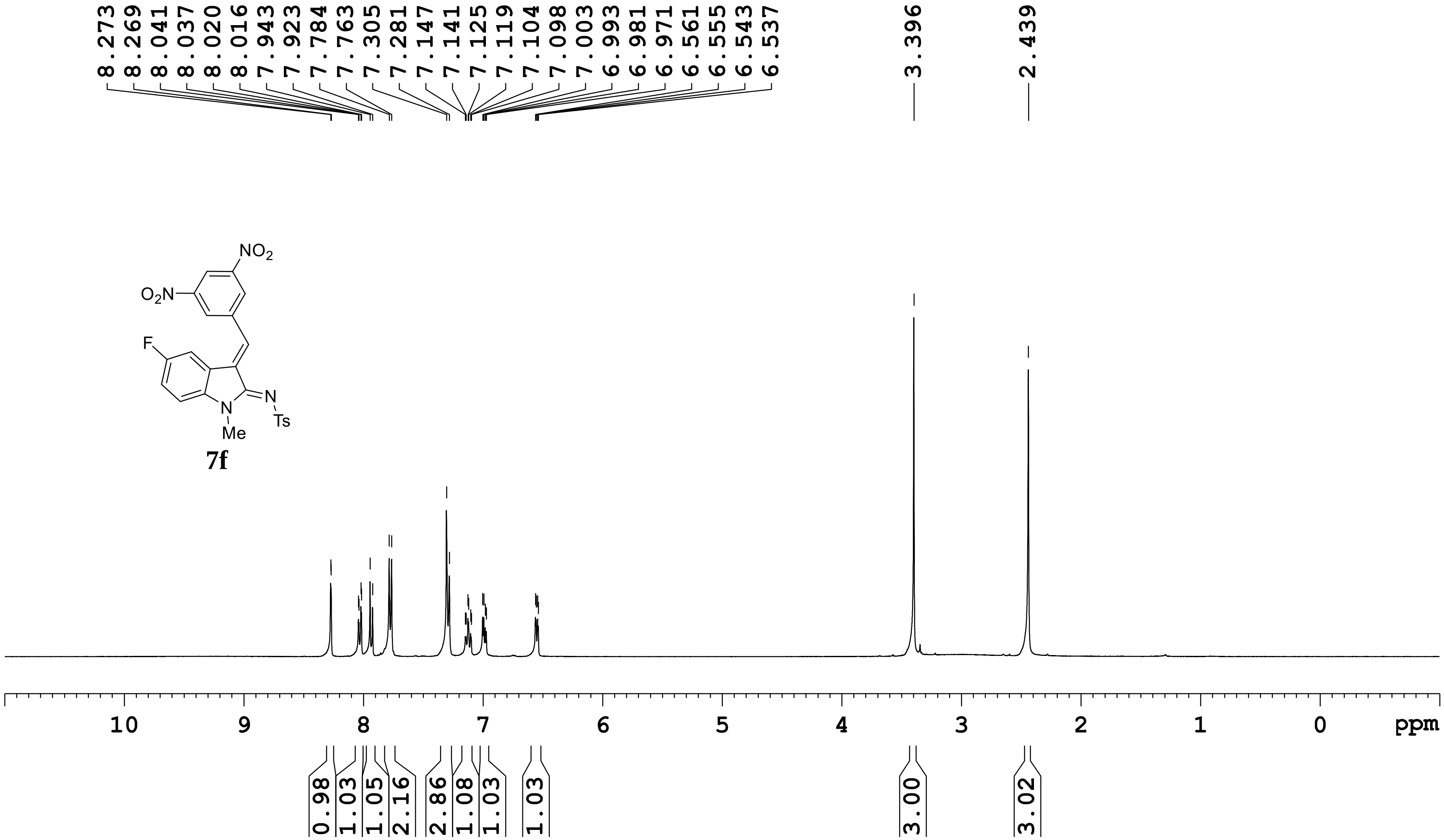

Spectra S46: ^{1}H NMR spectrum of **7f**

(3*E*)-N-Tosyl-3-[(3,5-dinitrophenyl)methylidene]-5-fluoro-2,3-dihydro-1-methylindol-2-amine

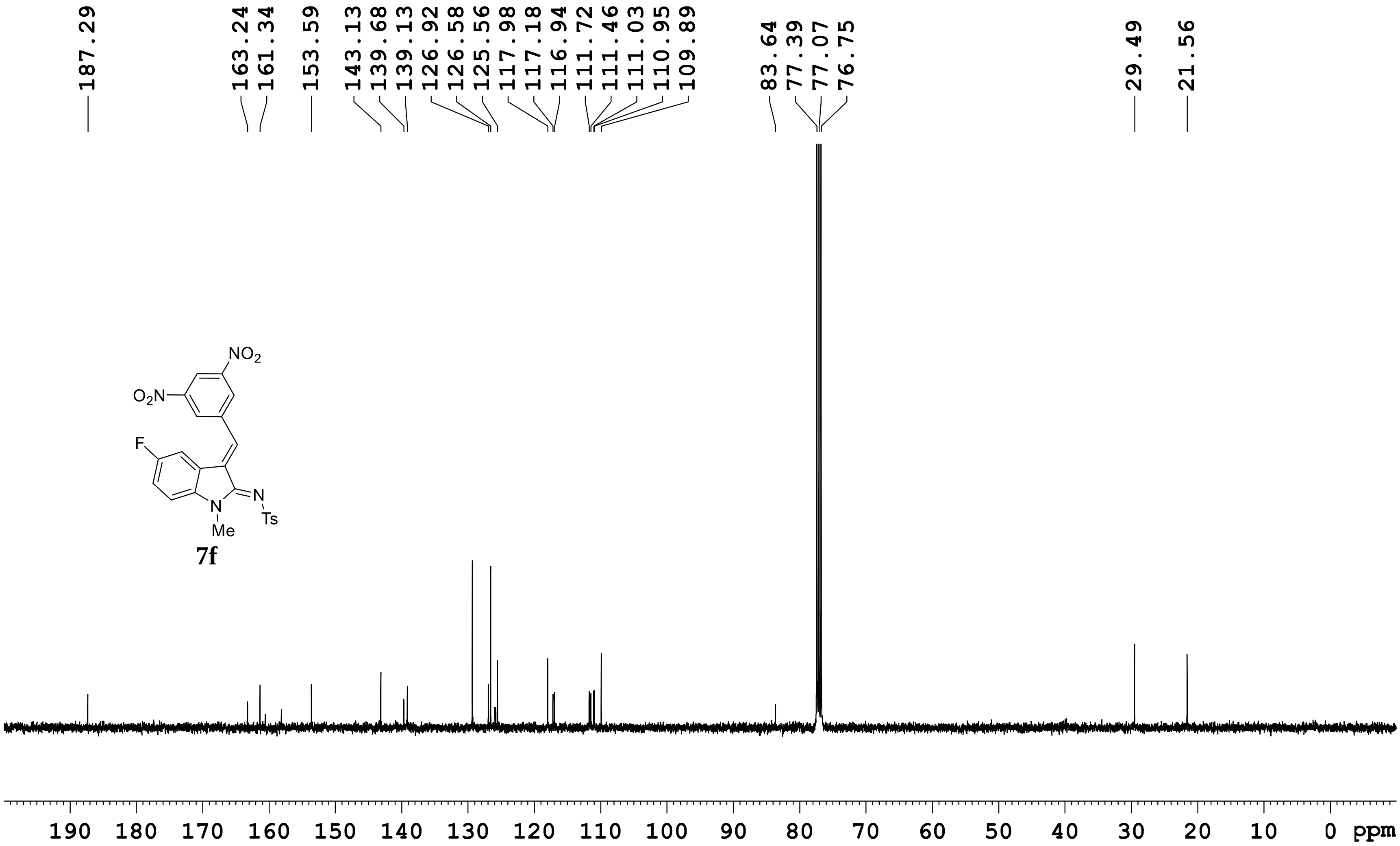

7f

Spectra S47: ^{13}C NMR spectrum of **7f**

(3*E*)-N-Tosyl-3-[(4-methylphenyl)methylidene]-2,3-dihydro-1-4-bromobenzyl-indol-2-amine

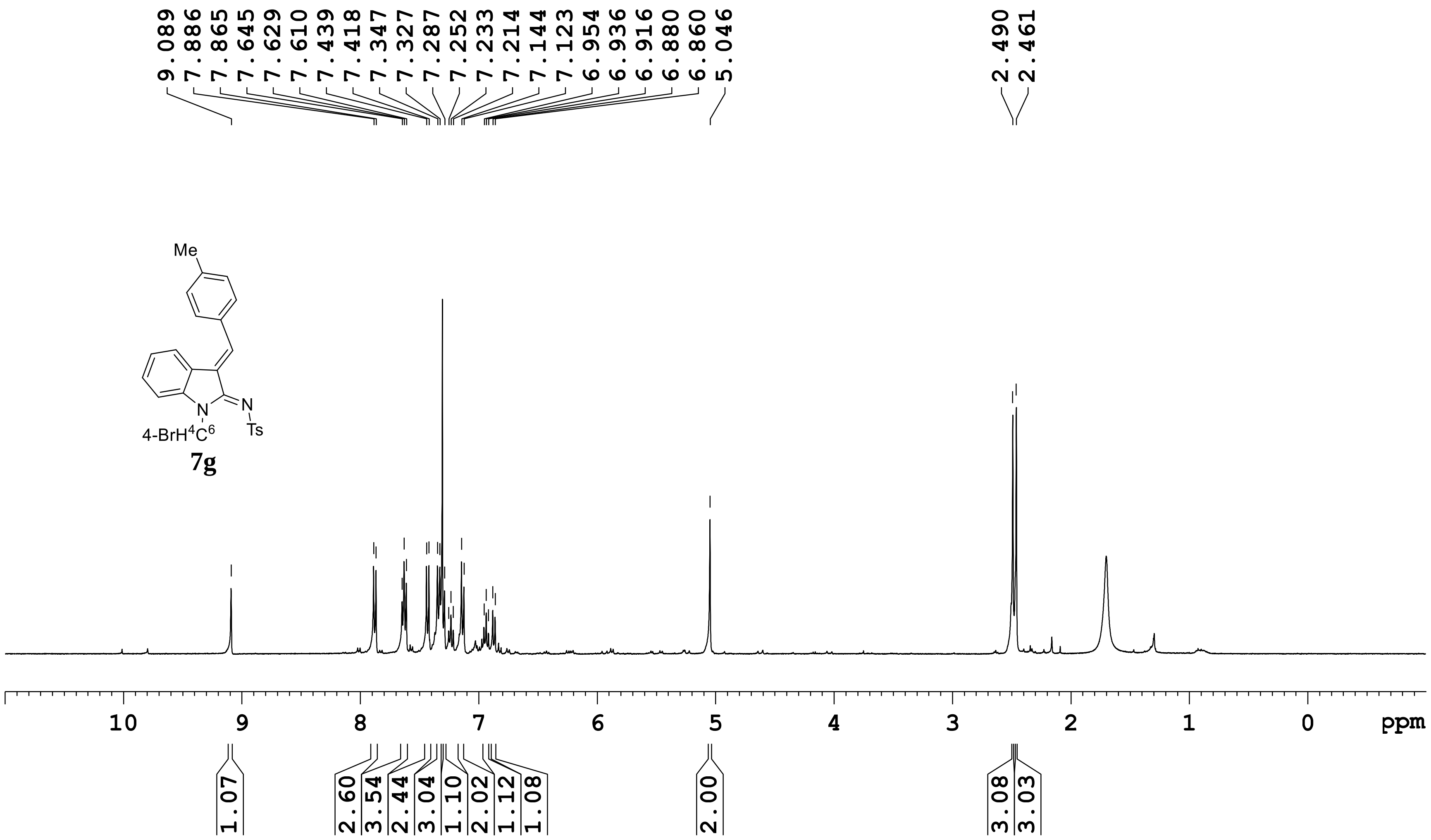

Spectra S48: ^{1}H NMR spectrum of **7g**

(3*E*)-N-Tosyl-3-[(4-methylphenyl)methylidene]-2,3-dihydro-1,4-bromobenzylindol-2-amine

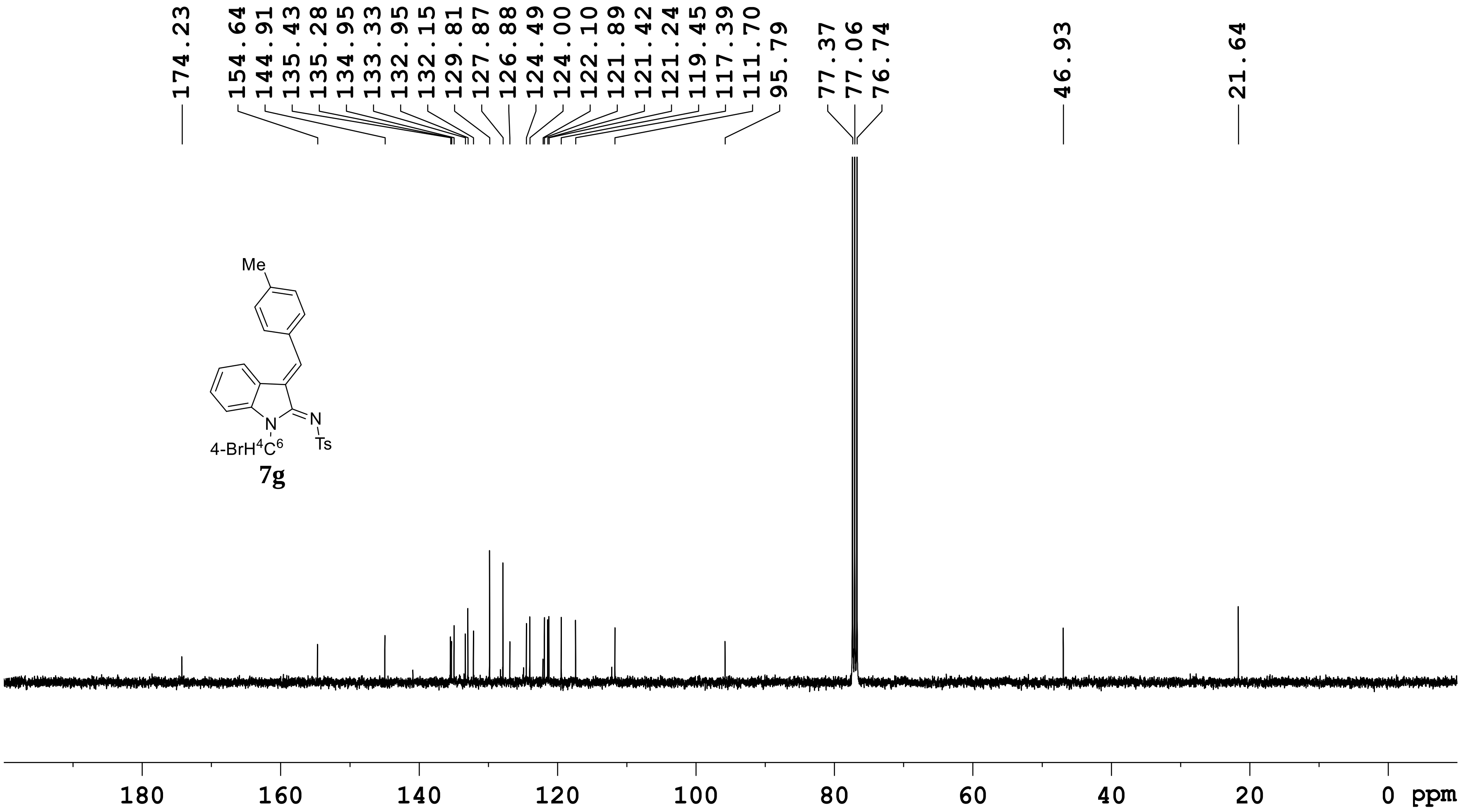

Spectra S49: ^{13}C NMR spectrum of **7g**

Scheme S5. Substrate scope for macrocycles (8a-h)

16-(Thiophen-3-yl)-5,11-dimethyl-6,10-ditosyl-5,6,7,8,9,10,11,16-octahydro-[1,5]diazecino[6,7-*b*:10,9-*b*']diindole

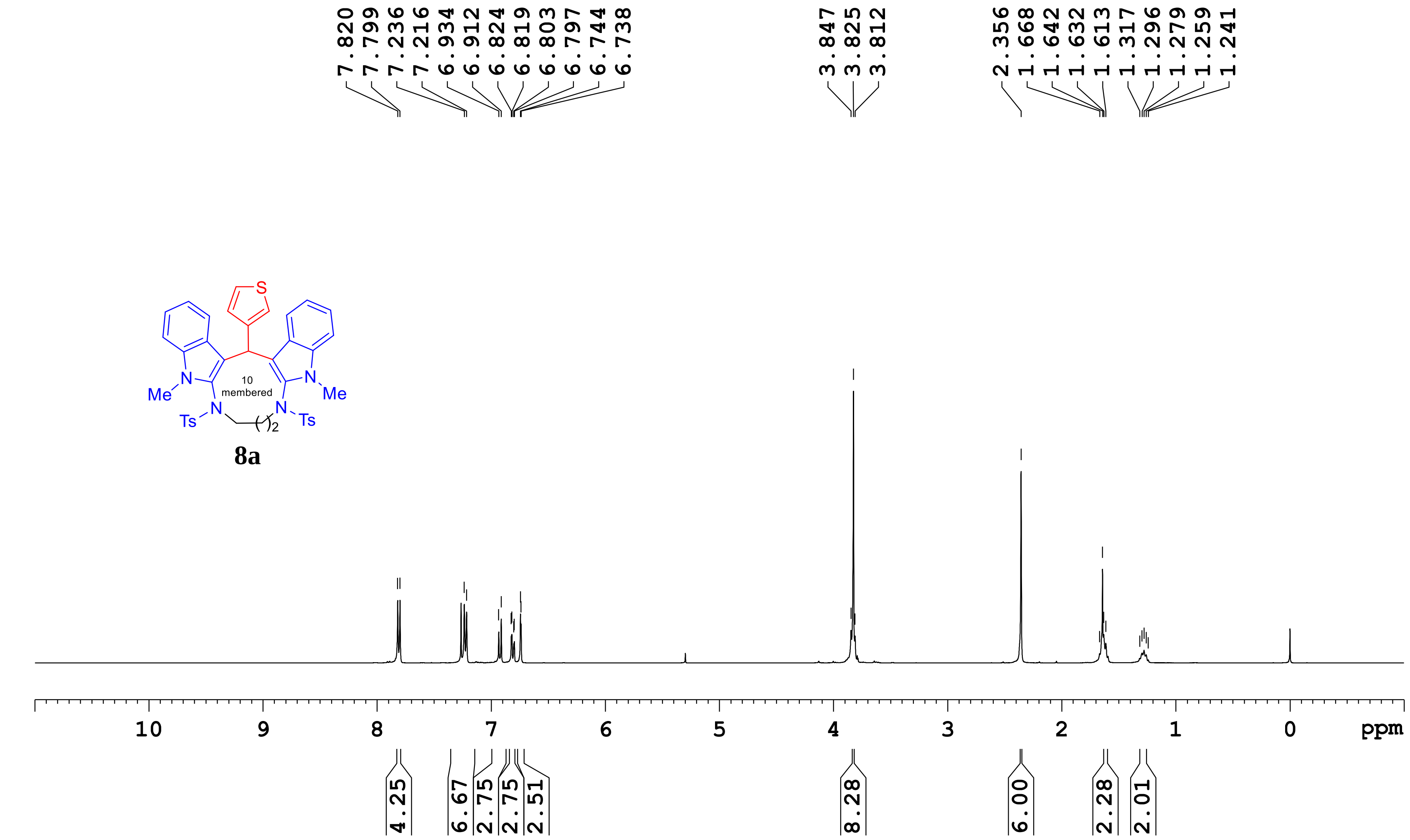

Spectra S50: ^{1}H NMR spectrum of **8a**

16-(Thiophen-3-yl)-5,11-dimethyl-6,10-ditosyl-5,6,7,8,9,10,11,16-octahydro-[1,5]diazecino[6,7-*b*:10,9-*b*']diindole

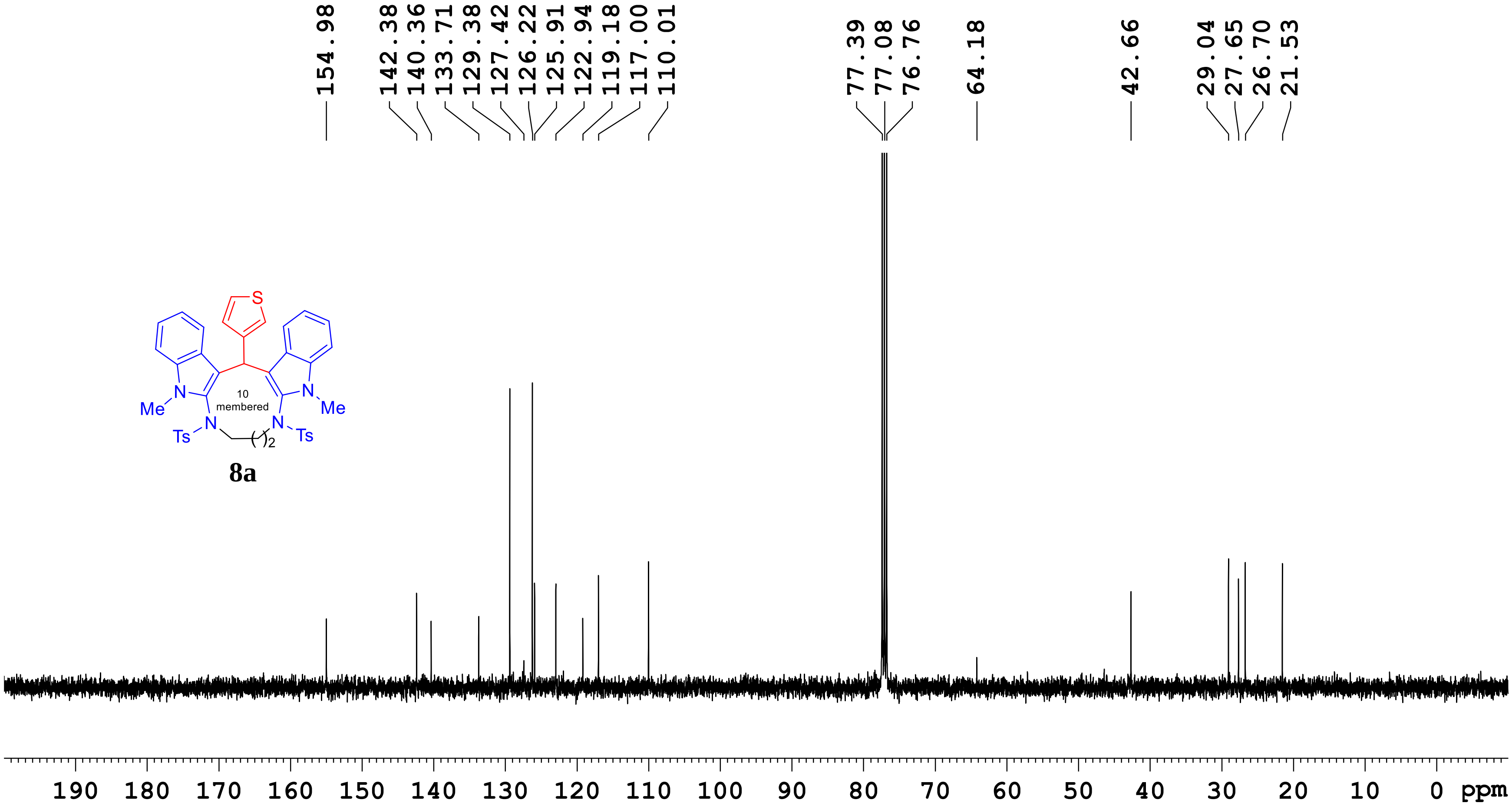

Spectra S51: ^{13}C NMR spectrum of **8a**

17-(3,4,5-Trimethoxyphenyl)-3,14-dibromo-5,12-dimethyl-6,11-ditosyl-6,7,8,9,10,11,12,17-octahydro-5H-[1,6]diazacycloundecino[7,8-*b*:11,10-*b'*]diindole

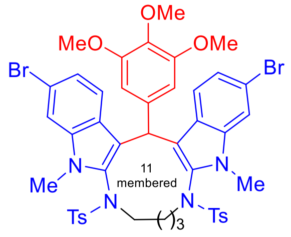

8b

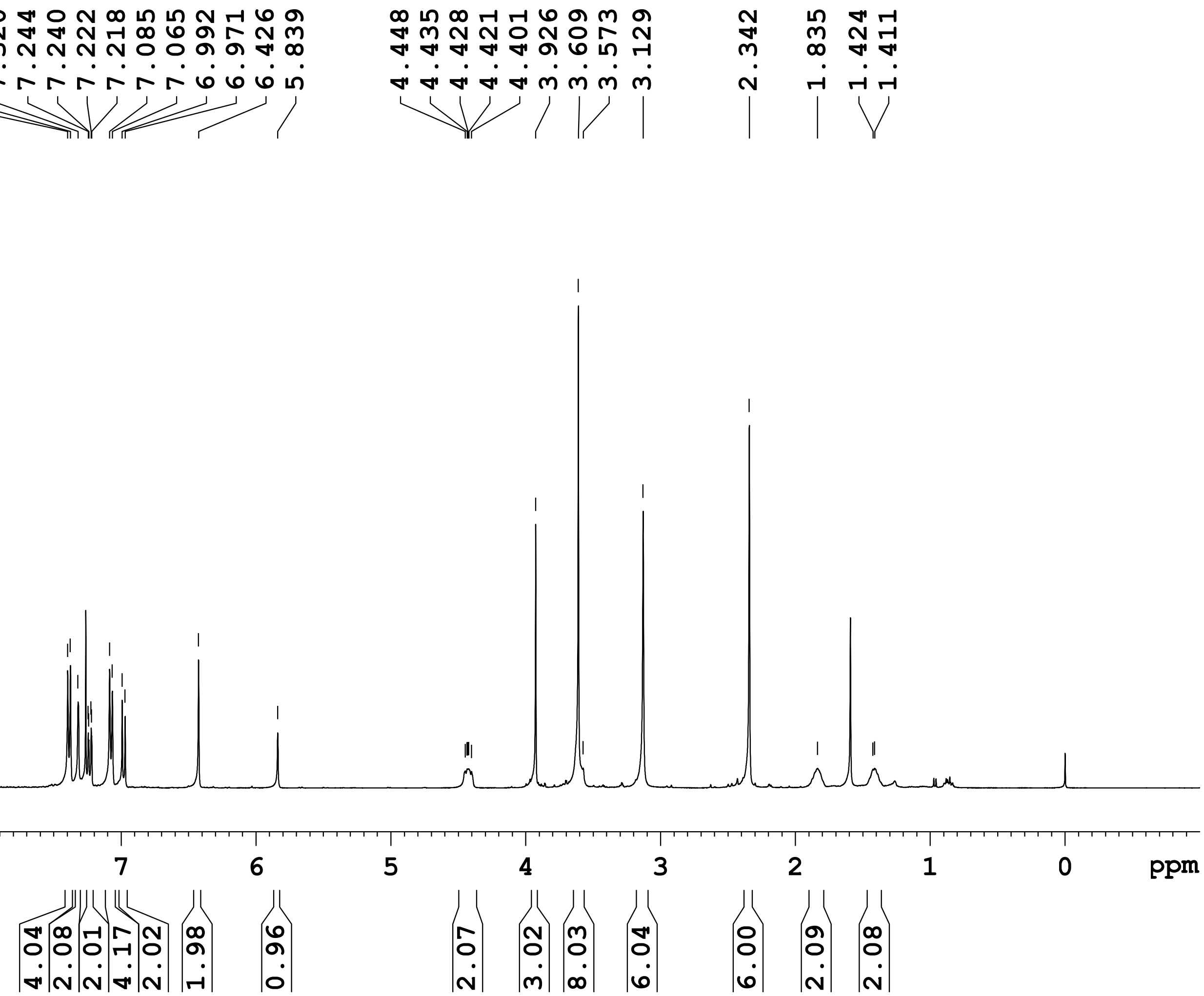

Spectra S52: ^{1}H NMR spectrum of **8b**

17-(3,4,5-Trimethoxyphenyl)-3,14-dibromo-5,12-dimethyl-6,11-ditosyl-6,7,8,9,10,11,12,17-octahydro-5H-[1,6]diazacycloundecino[7,8-*b*:11,10-*b*']diindole

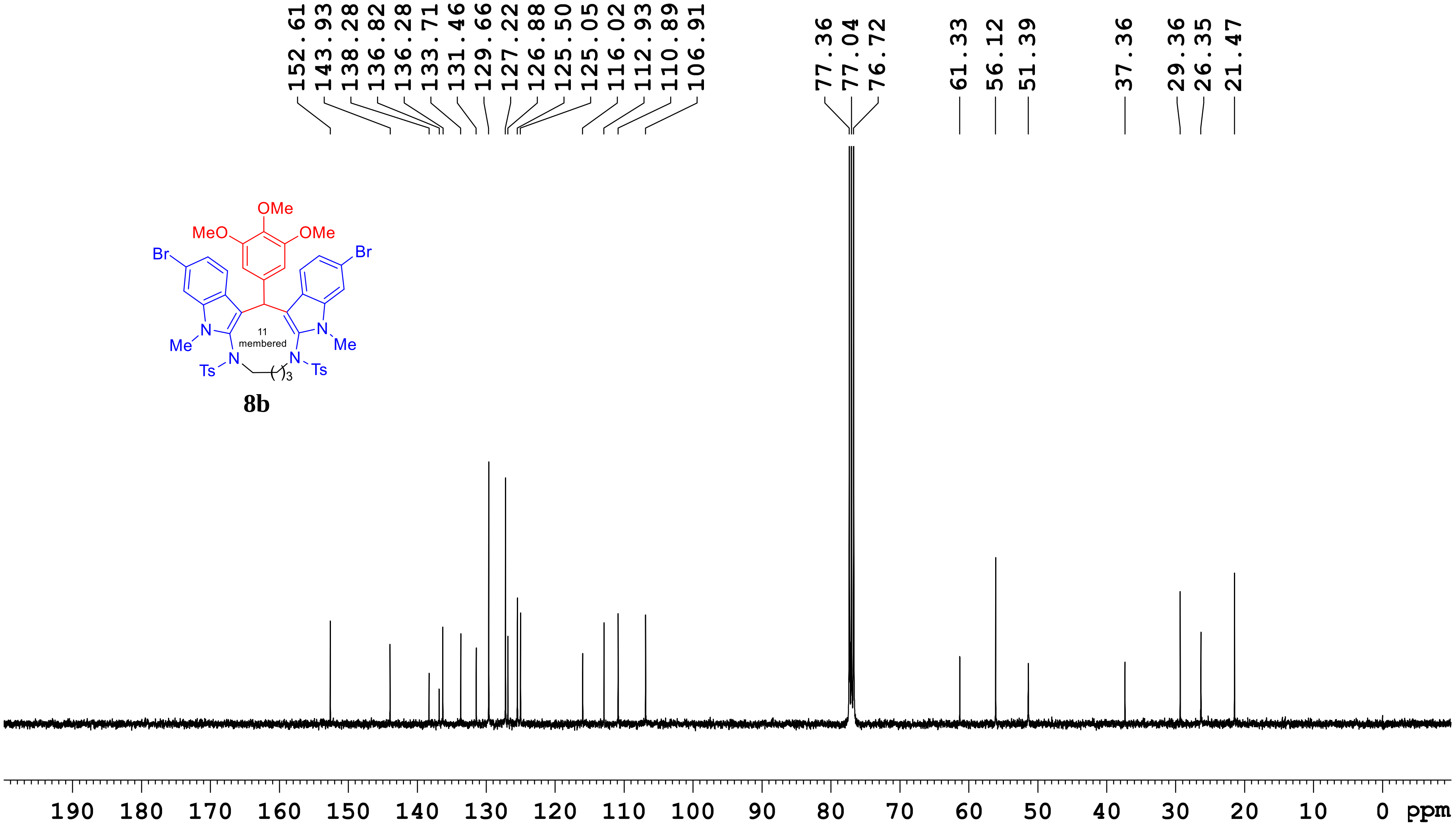

Spectra S53: ^{13}C NMR spectrum of **8b**

18-(*p*-Tolyl)-5,13-dimethyl-6,12-ditosyl-5,6,7,8,9,10,11,12,13,18-decahydro-[1,7]diazacyclododecino[2,3-*b*:6,5-*b*']diindole

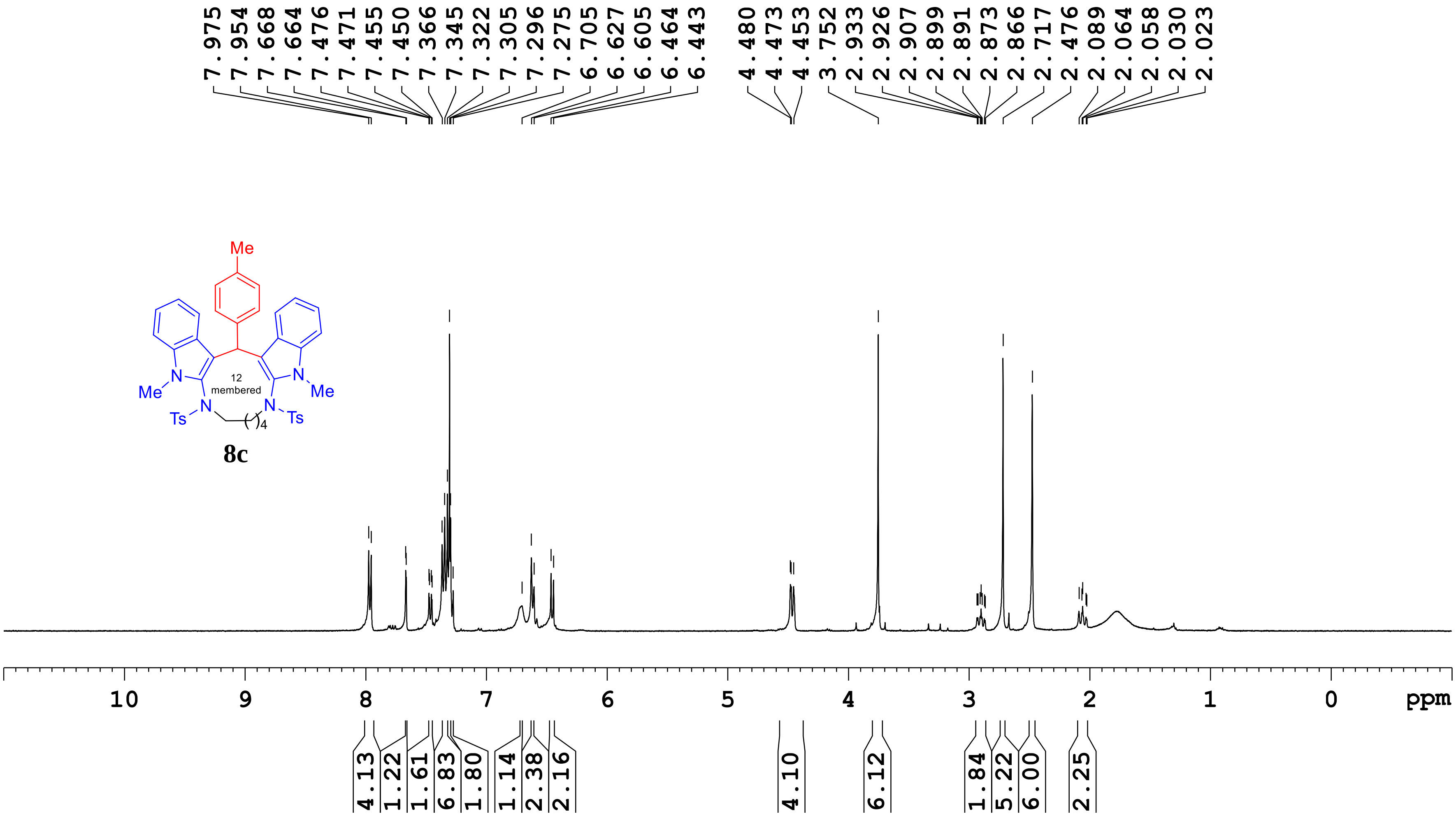

8c

Spectra S54: ^{1}H NMR spectrum of **8c**

18-(*p*-Tolyl)-5,13-dimethyl-6,12-ditosyl-5,6,7,8,9,10,11,12,13,18-decahydro-[1,7]diazacyclododecino[2,3-*b*:6,5-*b*']diindole

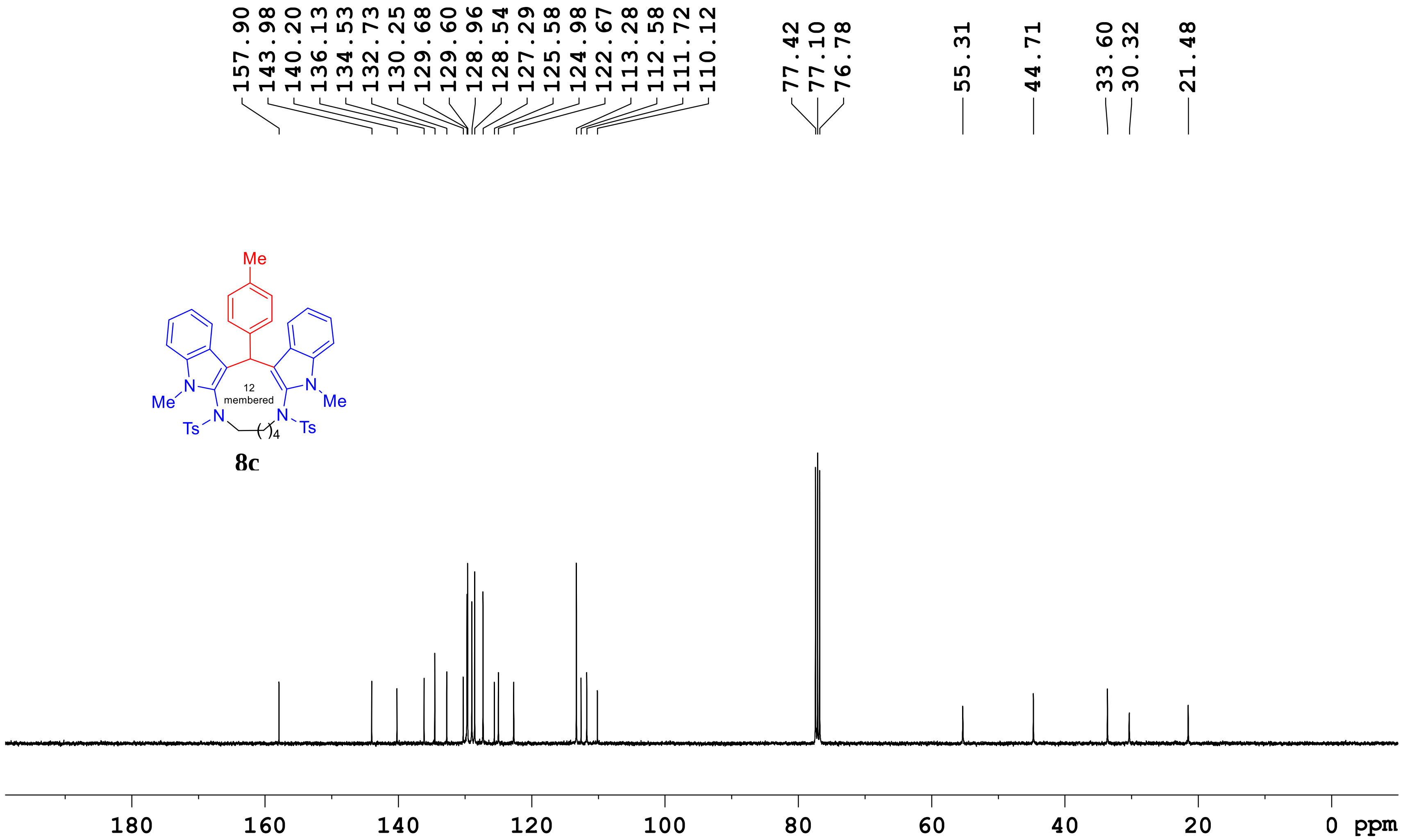

Spectra S55: ^{13}C NMR spectrum of **8c**

19-(4-Bromophenyl)-5,14-diethyl-6,13-ditosyl-6,7,8,9,10,11,12,13,14,19-decahydro-5H-[1,7]diazacyclotridecino[2,3-*b*:6,5-*b*']diindole

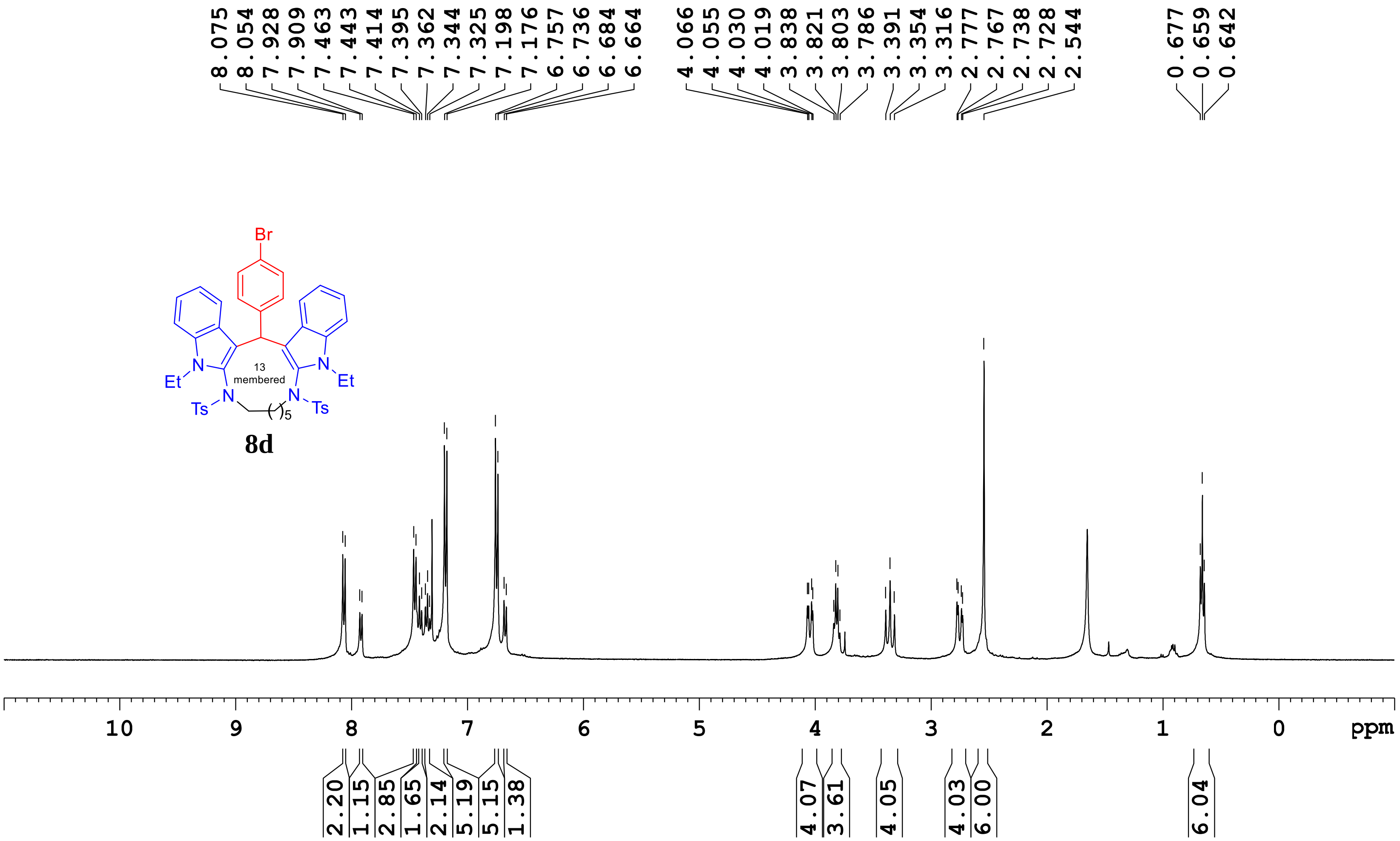

Spectra S56: ^{1}H NMR spectrum of **8d**

19-(4-Bromophenyl)-5,14-diethyl-6,13-ditosyl-6,7,8,9,10,11,12,13,14,19-decahydro-5H-[1,7]diazacyclotridecino[2,3-*b*:6,5-*b*']diindole

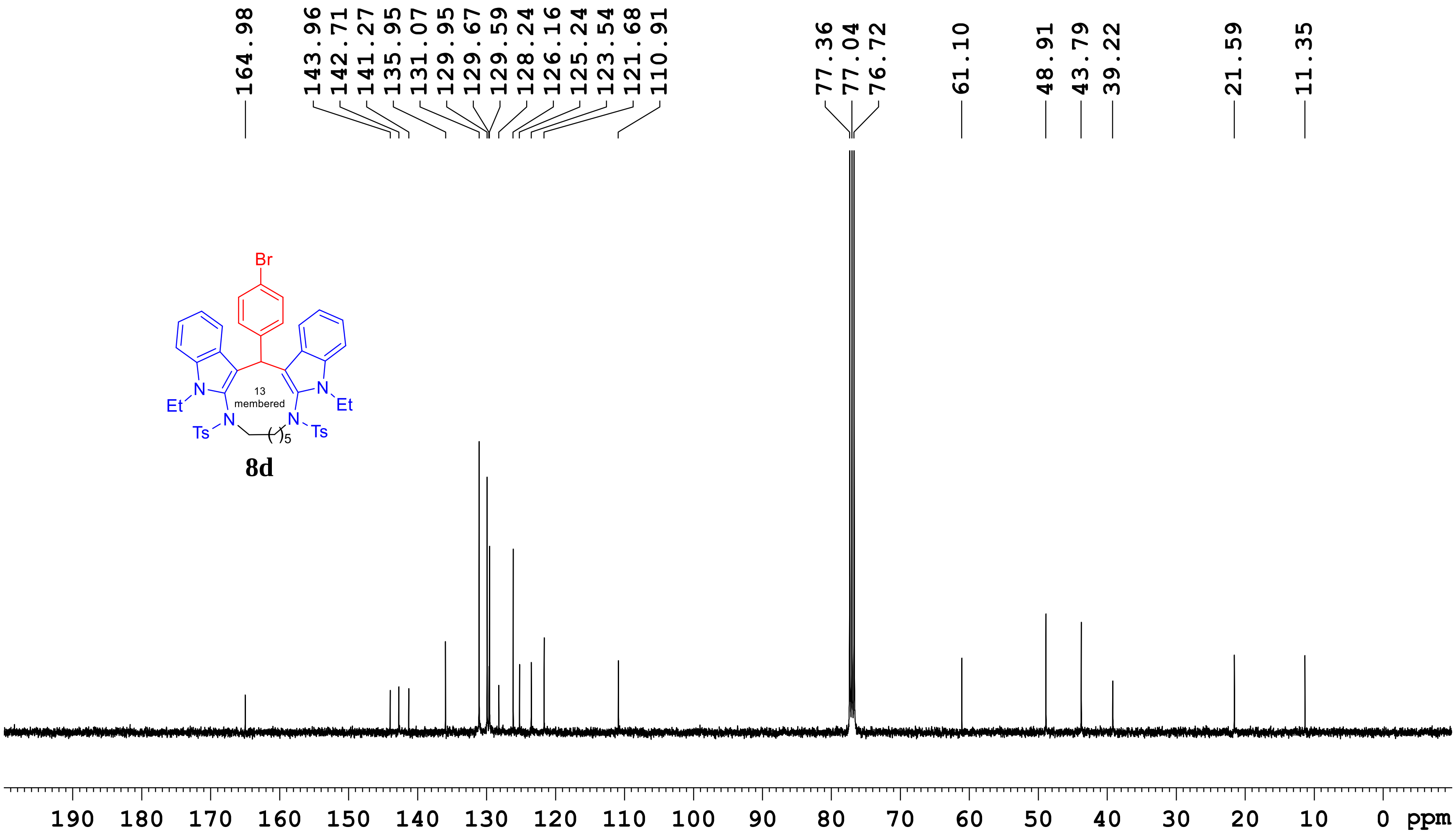

Spectra S57: ^{13}C NMR spectrum of **8d**

21-(4-Methoxyphenyl)-5,16-diethyl-6,15-ditosyl-6,7,8,9,10,11,12,13,14,15,16,21-dodecahydro-5H-[1,7]diazacyclopentadecino[2,3-b:6,5-b']diindole

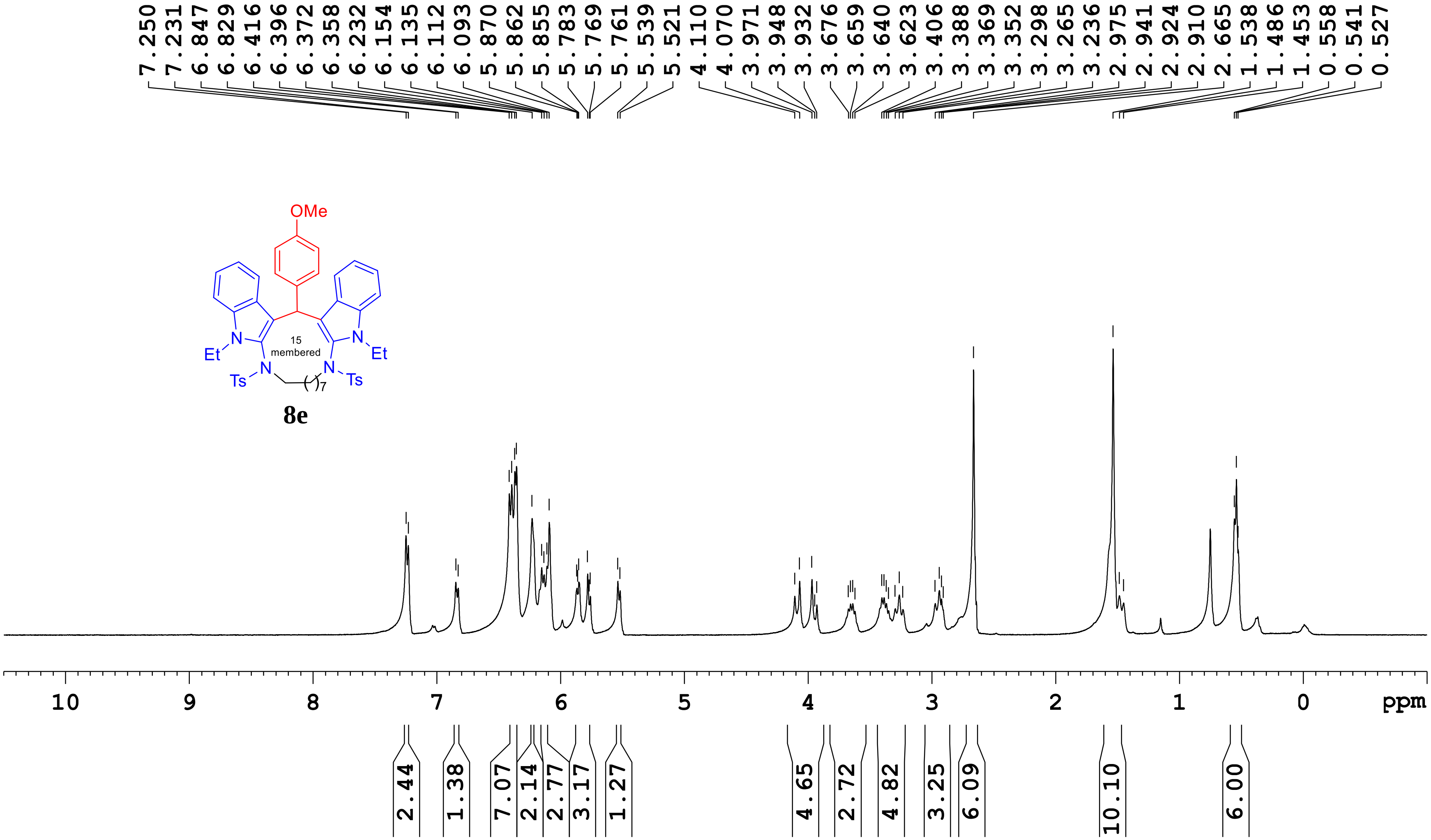

Spectra S58: ^{1}H NMR spectrum of **8e**

21-(4-Methoxyphenyl)-5,16-diethyl-6,15-ditosyl-6,7,8,9,10,11,12,13,14,15,16,21-dodecahydro-5H-[1,7]diazacyclopentadecino[2,3-b:6,5-b']diindole

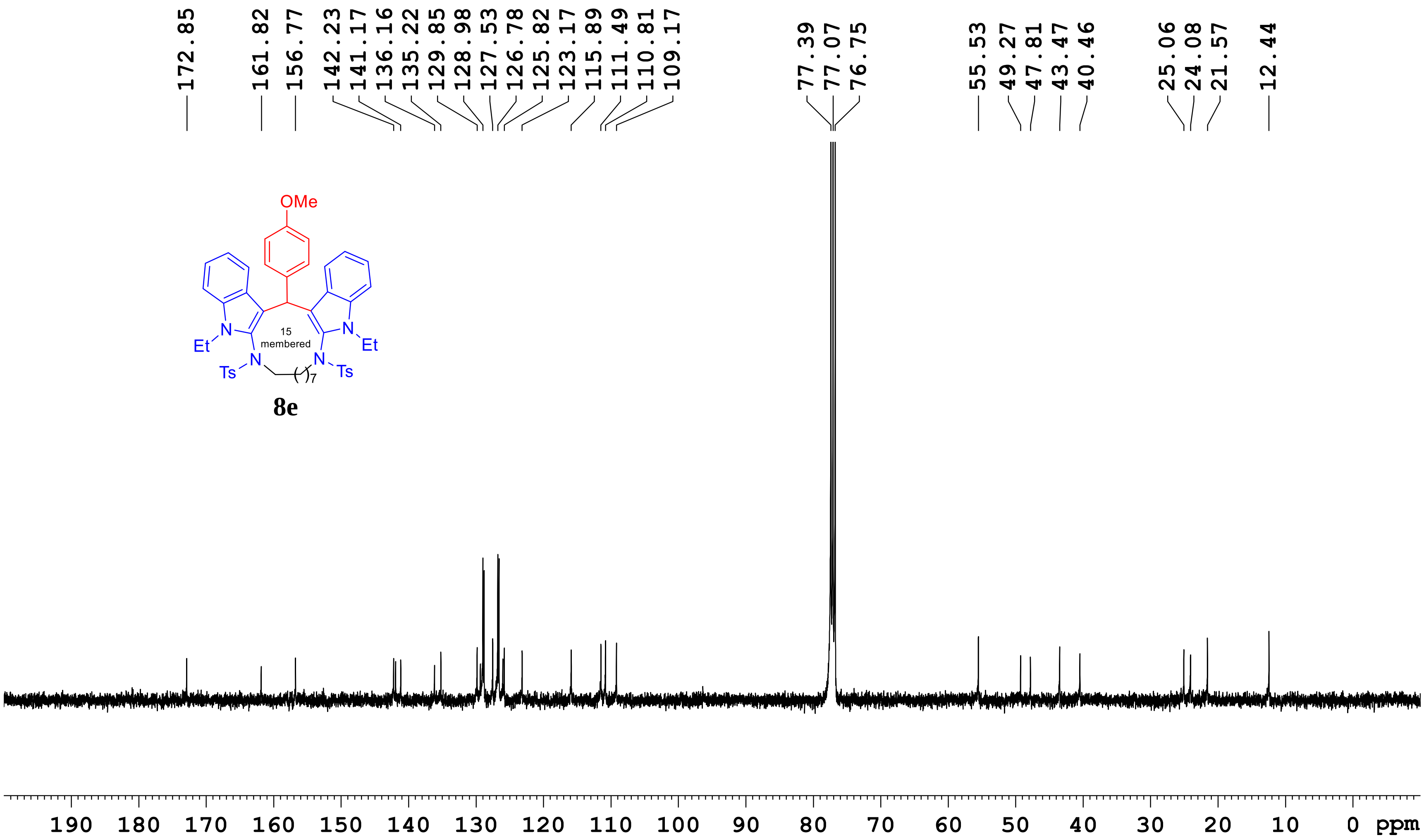

Spectra S59: ^{13}C NMR spectrum of **8e**

2-(4-Bromophenyl)-1^1,3^1-diethyl-4,8-ditosyl-1^{1}H,3^{1}H-4,8-diaza-1,3(3,2)-diindola-6(1,4)-benzenacyclooctaphane

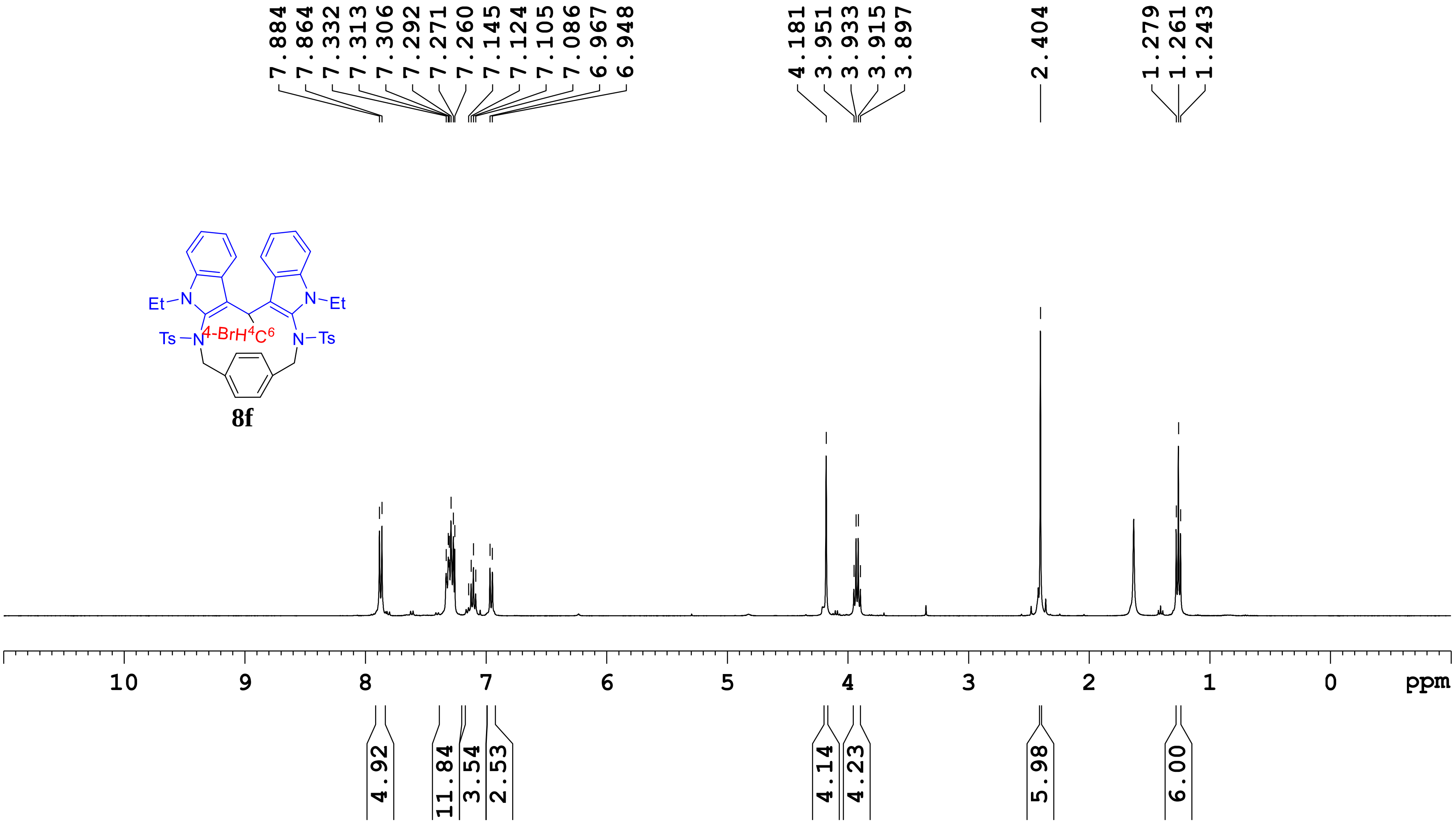

8f

Spectra S60: ^{1}H NMR spectrum of **8f**

2-(4-Bromophenyl)-1^{1},3^{1}-diethyl-4,8-ditosyl-1^{1}H,3^{1}H-4,8-diaza-1,3(3,2)-diindola-6(1,4)-benzenacyclooctaphane

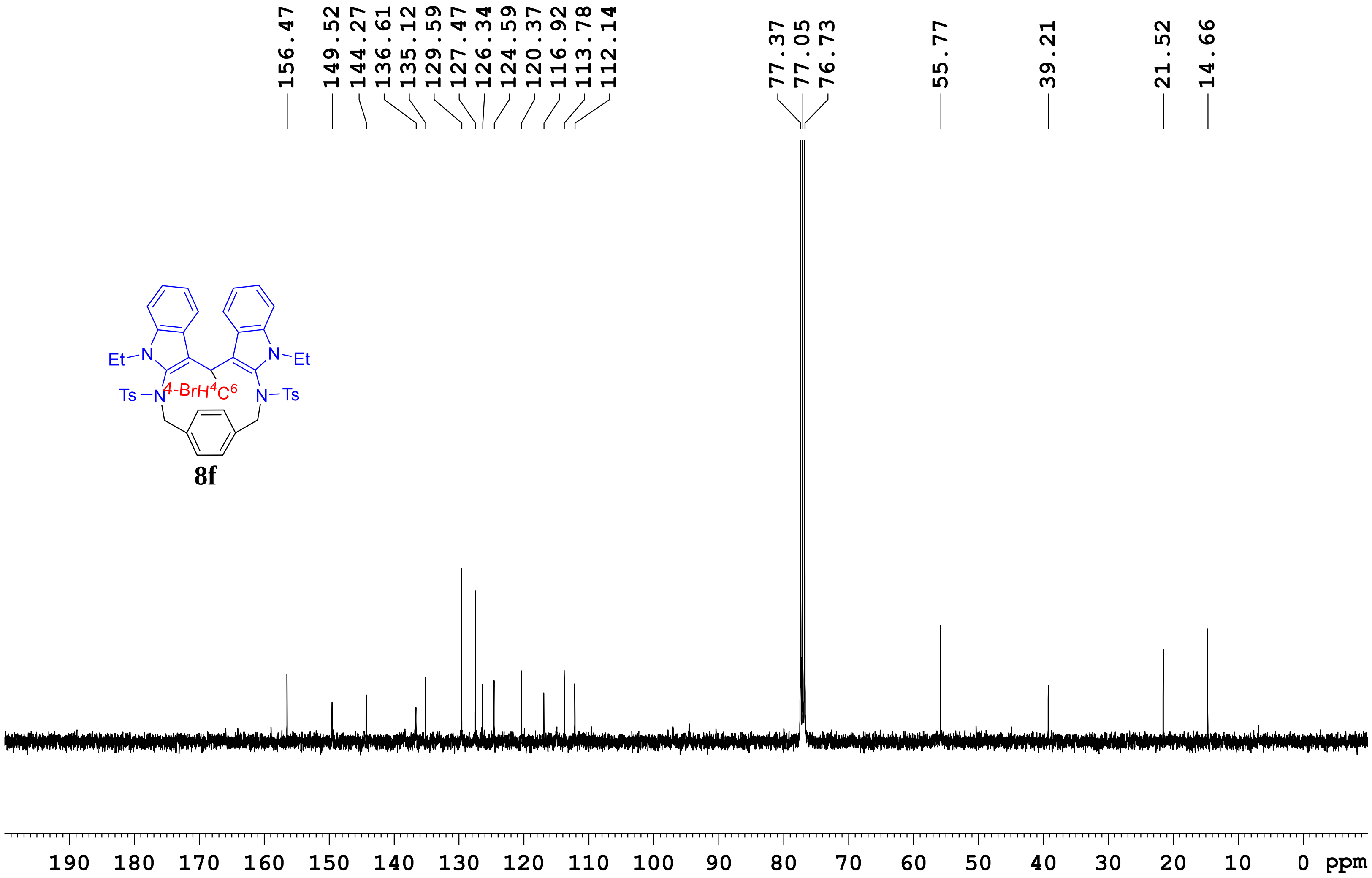

Spectra S61: ^{13}C NMR spectrum of **8f**

1[1],3[1]-dibenzyl-2-phenyl-4,8-ditosyl-1[1]H,3[1]H-4,8-diaza-1,3(3,2)-diindola-6(1,4)-benzenacyclooctaphane

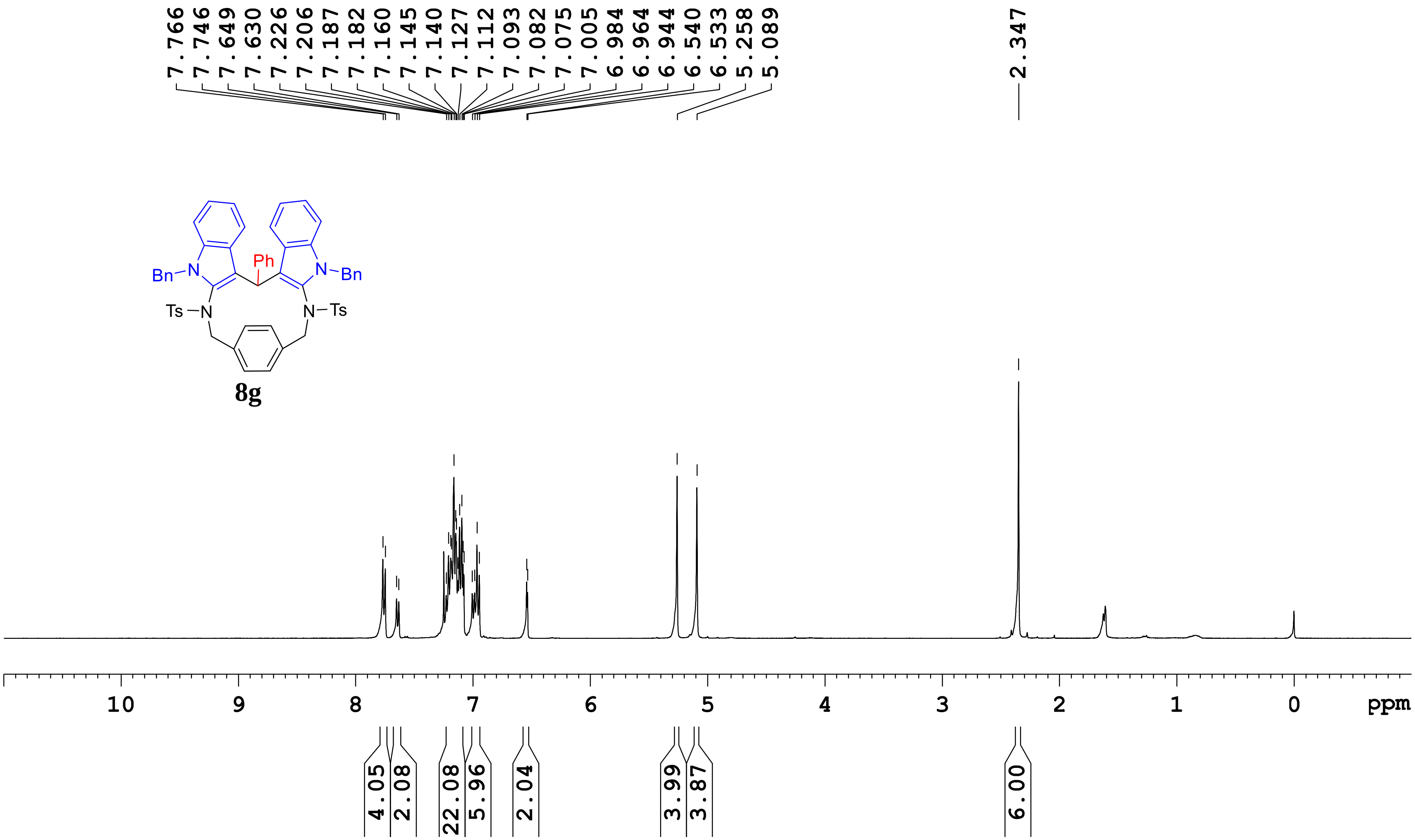

Spectra S62: ^{1}H NMR spectrum of **8g**

1^1,3^1-dibenzyl-2-phenyl-4,8-ditosyl-1^1H,3^1H-4,8-diaza-1,3(3,2)-diindola-6(1,4)-benzenacyclooctaphane

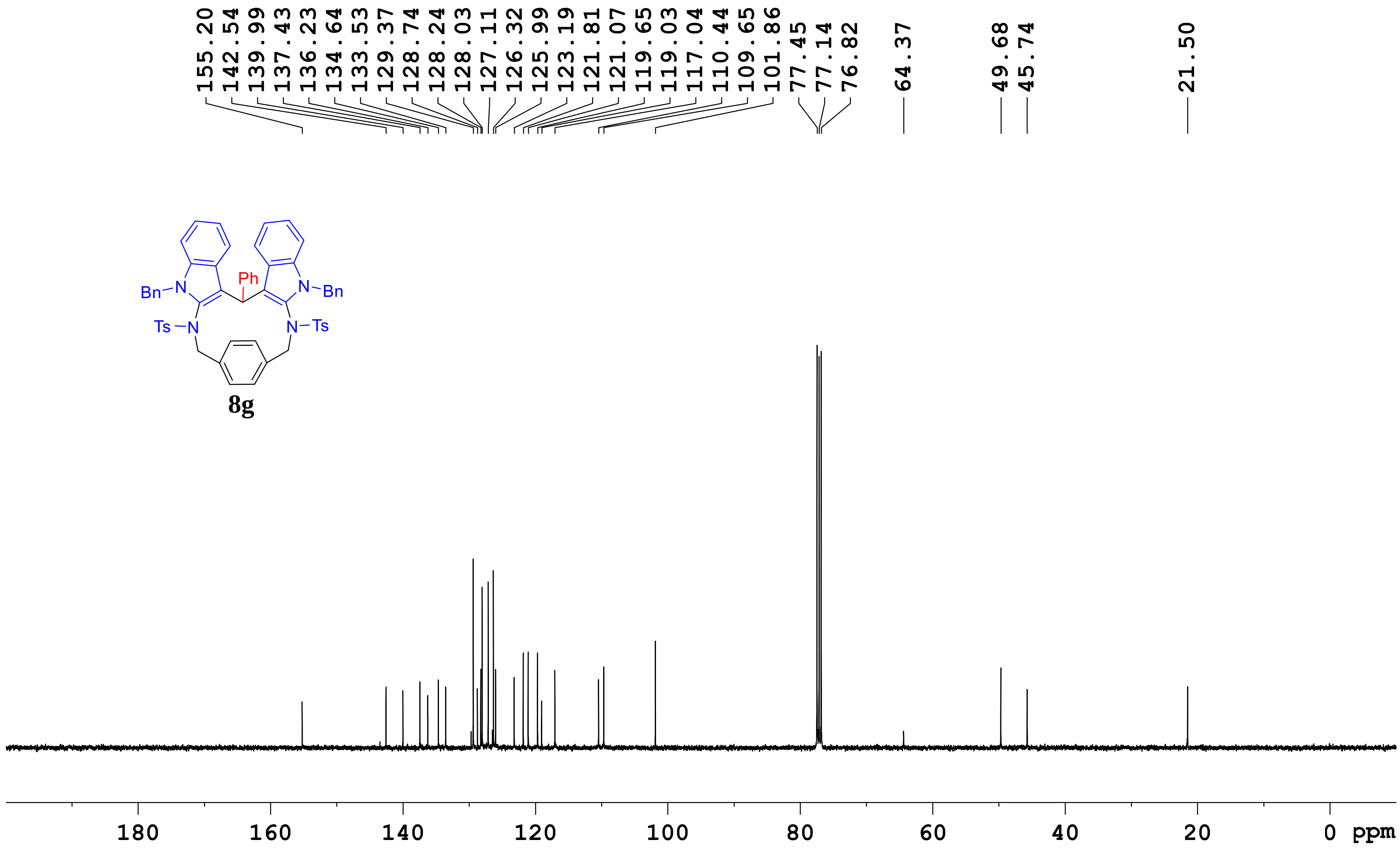

Spectra S63: ^{13}C NMR spectrum of **8g**

1[1],3[1]-dimethyl-2-(thiophen-3-yl)-4,8-ditosyl-1[1]H,3[1]H-4,8-diaza-1,3(3,2)-diindola-6(1,3)-benzenacyclooctaphane

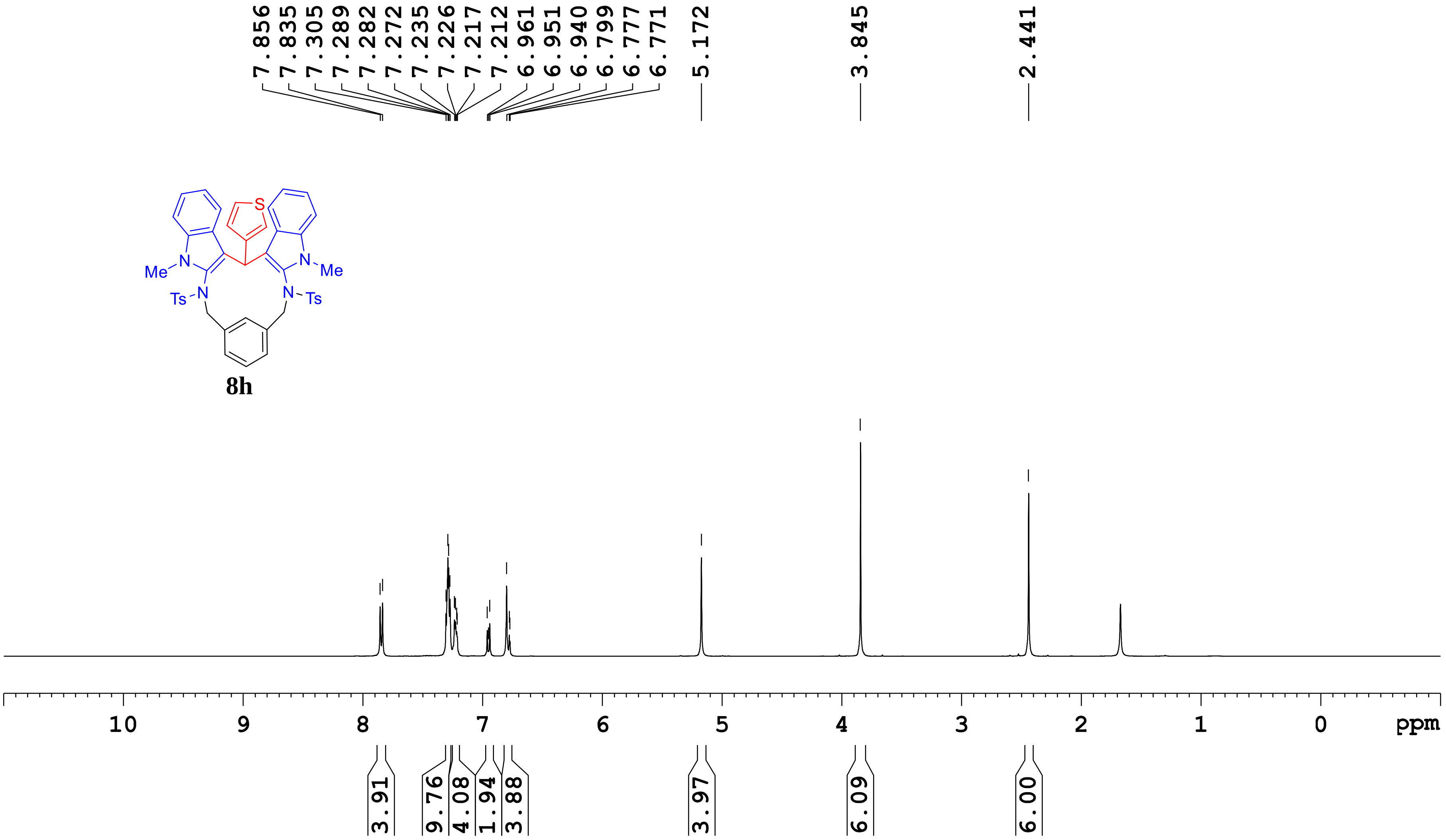

Spectra S64: ^{1}H NMR spectrum of **8h**

1[1],3[1]-dimethyl-2-(thiophen-3-yl)-4,8-ditosyl-1[1]H,3[1]H-4,8-diaza-1,3(3,2)-diindola-6(1,3)-benzenacyclooctaphane

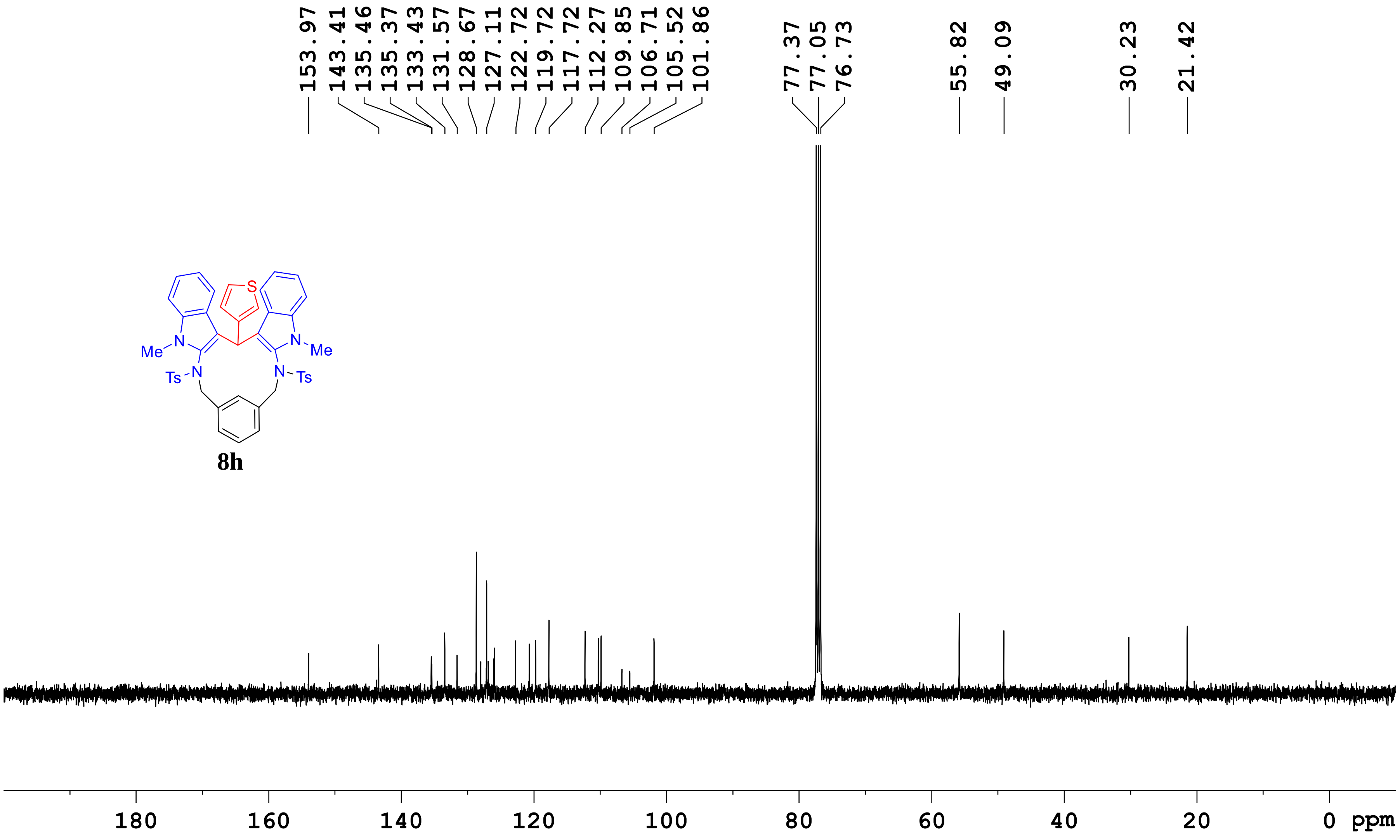

Spectra S65: ^{13}C NMR spectrum of **8h**
